# Application of Bulky NHC–Rhodium Complexes in Efficient S–Si and S–S Bond Forming Reactions

**DOI:** 10.1021/acs.inorgchem.1c02160

**Published:** 2021-11-05

**Authors:** Małgorzata Bołt, Patrycja Żak

**Affiliations:** Department of Organometallic Chemistry, Faculty of Chemistry, Adam Mickiewicz University in Poznań, Uniwersytetu Poznańskiego 8, 61-614 Poznań, Poland

## Abstract

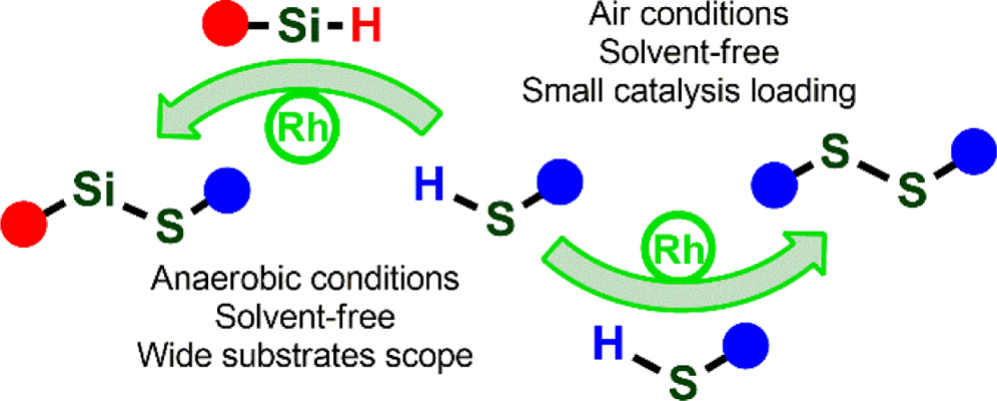

The efficient and straightforward syntheses of silylthioethers and disulfides are presented. The synthetic methodologies are based on new rhodium complexes containing bulky N-heterocyclic carbene (NHC) ligands that turned out to be efficient catalysts in thiol and thiol–silane coupling reactions. These green protocols, which use easily accessible reagents, allow obtaining compounds containing S–Si and S–S bonds in solvent-free conditions. Additionally, preliminary tests on coupling of mono- and octahydro-substituted spherosilicates with selected thiols have proved to be very promising and showed that these catalytic systems can be used for the synthesis of a novel class of functionalized silsesquioxane derivatives.

## Abstract 2

Two novel rhodium complexes bearing bulky N-heterocyclic carbene ligands have been synthesized and isolated. Both compounds enable efficient and selective formation of silylthioethers and disulfides in solvent-free conditions. A comparison between bulky NHC−rhodium complexes and their smaller derivatives shows that the steric hindrance around the metal center is crucial for effective formation of a product in both catalytic reactions. It has also been proved that the catalysts can be reused several times without loss of activity.

## Introduction

Although organosulfur compounds are associated primarily with unpleasant odors, they play an extremely important role in many areas of life and science. One type of these compounds is silylthioethers, which can be used as protecting groups for carbonyl compounds, a convenient source of sulfur atoms^1^ and substrates for Z-silyl enol ethers, which are useful synthons in organic synthesis.^2^ The Si–S bond can also be activated in a reaction with aryl chlorides giving diaryl thioethers.^3^ Recently, the use of silylthioethers in the process of creating redox-responsive poly(phenylene sulfide)-based gels has been described.^4^ Despite the great practical importance of silylthioethers, only a limited number of methods for their synthesis have been presented as yet.^5^ It is possible to prepare these compounds in reactions of chlorosilanes with metal sulfides.^6^ However, this method is characterized by a weak atom economy and the use of corrosive reagents. Recently, much attention has been focused on the catalytic methods employing Ru,^7,8^ Fe,^9^ and Ni^10^ complexes. However, these processes usually demand a large amount of a catalyst and the use of other additives. B(C_6_F_5_)_3_ has also been proved to be an efficient catalyst for preparation of silylthioethers, but this method has been described for a very narrow range of reagents, so it is difficult to define its universality.^11^

An equally important group of compounds is the disulfides, which are very significant species in biological systems.^12^ An example of the well-known compounds containing the S–S bond is cystine, which is part of numerous proteins and it plays a crucial role in folding processes and stabilization of the secondary structure.^13^ Disulfide bonds also occur in enzymes that act as reducers of cystine in living organisms and may play an important role in regulating the oxidative stress.^14^ In addition to the naturally occurring systems containing the S–S bond, the importance of this moiety in many drugs and therapeutics should be emphasized.^15^ Recently, it has been found that one of the key proteins of SARS-CoV-2019 binds to the receptor in the host cell by forming a disulfide bond with cysteine residues.^16^

Among the numerous methods of disulfide synthesis,^17^ we can distinguish catalytic oxidation of thiols catalyzed by Ru,^8^ Mn,^18^ Fe,^19^ and Cu.^20^ Although these methods efficiently lead to S–S bond formation, they are often characterized by the need to use a large amount of catalyst and other additives. Several photocatalytic processes for preparation of disulfides have also been described, usually using hazardous solvents.^21^ The use of rhodium catalysts in this reaction has been explored; however, the number of examples of their use is limited.^22−24^ Therefore, further research on the use of rhodium catalysts in the synthesis of organosulfur compounds is highly desirable.

Herein, we report the synthesis of rhodium catalysts containing bulky NHC ligands and their application in disulfide and silylthioether formation. We also describe the possibility of functionalization of spherosilicates with thiols.

## Results and Discussion

### Synthesis of NHC–Rhodium Complexes

**Scheme 1:**
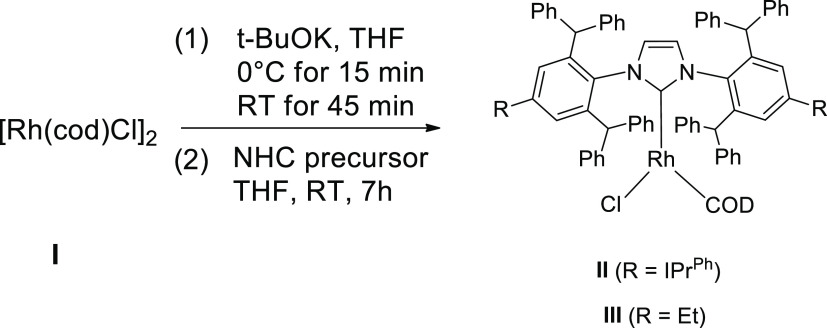
Synthesis of NHC–Rhodium Complexes (**II**, **III**)

We began our research with the synthesis of new types of NHC–rhodium complexes. They were isolated as yellow solids in yields of 91 (**II**) and 93% (**III**) according to the methodology described by Markó’s group with modifications^25^ (Scheme 1).

The resulting complexes are air stable, easy to handle, and can be synthesized on the gram scale. They were characterized by spectroscopic and mass spectrometric analyses (see the Supporting Information). Unfortunately, although we tried hard, we have been unable to obtain monocrystals of complexes **II** and **III**, suitable for the X-ray diffraction (XRD) analysis.

### Formation of the S–Si Bond

**Scheme 2:**
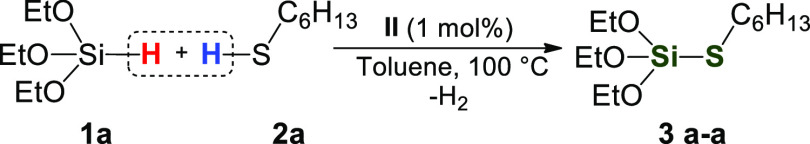
Coupling between **1a** and **2a**

In the next step, the synthesized complexes were tested as precatalysts in a thiol–silane coupling reaction. As model substrates to optimize the reaction conditions, triethoxysilane (**1a**) and 1-hexanethiol (**2a**) were selected. The treatment of an equimolar mixture of **1a** and **2a** in toluene at 100 °C with 1 mol % **II** led to clean formation of the expected product in quantitative yield, as revealed by the gas chromatography–mass spectrometry (GC–MS) analysis (Scheme 2).

A similar result was observed when the process was carried out in the presence of **III**. Therefore, henceforth, only the results obtained for **II** are reported. The results obtained using **III** are included in the Supporting Information. To evaluate the effect of the solvent, temperature, reaction time, and loading of the catalyst, a series of additional catalytic tests were performed. The results are summarized in Table 1.

**Table 1:** Optimization of the Type of Solvent and Temperature^a^

entry	solvent	*T* [°C]	*t* [h]	yield of 3aa [%]^e^
1	DCM	45	24	6
				
2	toluene	45	24	40
3		60	24	85
4		80	24	90
5		90	24	99
6		100	6	100
7		110	3	100
				
8	water	80	24	85
9		100	24	97
10^b^		100	8	7
				
11	without solvent	80	24	88
12		100	24	93
13		**110**	**4**	**100**
14^c^		110	24	75
				
15^d^	water/toluene	100	5	100

a Reaction conditions: [**1a**]:[**2a**] = 1:1, **II** = [RhCl(cod)(IPr*^Ph^)], and [**II**] = 1 mol %, argon.

b Air atmosphere, 90% yield of disulfide was detected.

c [**II**] = 0.5 mol %.

d Volume ratio: 3:1.

e Determined by GC and GC–MS analyses.

As presented in Table 1, the effectivity of the reaction depended on the temperature and type of solvent used. The obtained results indicate that lowering the temperature below 90 °C led to a reduction in the yield, even though the reaction time was increased to 24 h (entry 1–4, 8, 11). We discovered that the process can be carried out under solvent-free conditions, which is very attractive for economic and ecological reasons (entries 11–14). An alternative to solid-state substrates is degassed water, toluene, or their mixture as the reaction medium. The efficiency of the process was independent of the amount of solvent used. Therefore, in the case of both solid reagents, the minimum amount of solvents can be used. In comparison, when dichloromethane (DCM) was used as a solvent in this process, we did not observe satisfactory conversion (entry 1). The manner in which the reaction was conducted had a significant impact on the selectivity of the thiol–silane coupling reaction. This process must be carried out under dry argon, using standard Schlenk-line and vacuum techniques. Otherwise, the main product of the reaction is disulfide (entry 10). In the optimum reaction conditions (entry 13), the catalytic properties of **II** and **III** in the model reaction were compared with selected rhodium complexes such as [Rh(cod)Cl]_2_ (**I**), RhCl_3_·3H_2_O (**IV**), [RhCl(cod)(Mes)] (**V**), and [RhCl(cod)(IPr)] (**VI**) (Table 2).

**Table 2:** Optimization of the Type of Catalyst^a^

entry	catalyst	*t* [h]	yield of 3aa^c^ [%]
1	**IV**	48	0
2	**I**	48	0
3	**II**	4	100
4^b^	**II**	24	85
5	**III**	7	99
6	**V**	48	31
7	**VI**	48	44
8	without catalyst	72	0

a Reaction conditions: solvent-free, argon, 110 °C, [**1a**]:[**2a**] = 1:1, and [**Rh**] = 1 mol %.

b [**Rh**] = 0.5 mol %.

c Determined by GC and GC–MS analyses.

The complexes with simple NHC ligands (entries 6 and 7) were found to exhibit a significantly lower catalytic activity than that of **II** and **III** (entry 3-5). No conversion was observed for catalysts **I** (entry 2), **IV** (entry 1) and for reaction without catalys (entry 8). These results did not surprise us because the impact of steric crowding of the ligand on the activity of transition-metal complexes has been described in the literature. It has been proved that NHC ligands of supersterical properties are responsible for screening of the metal center, which hinder potential regroupings that can take place in transition states (higher selectivity) and facilitate the process of reductive elimination, which may result in higher catalytic activity.

**Scheme 3:**
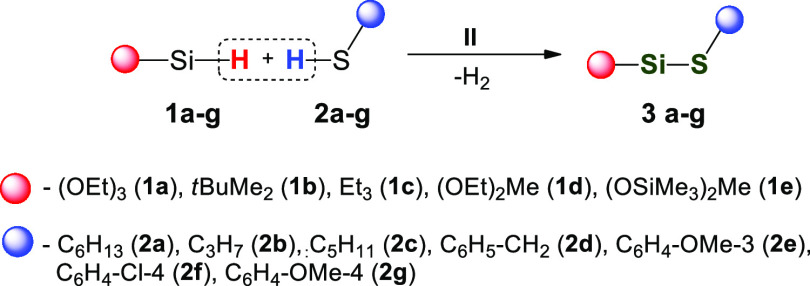
Formation of the Si–S Bond Catalyzed by **II**

With optimized reaction conditions in hand, we determined the reactivities of a series of silanes with selected thiols (Scheme 3).

Seven different thiols containing alkyl (**2a–c**), benzyl (**2d**), or substituted phenyl groups located at the sulfur atom (**2e–g**) were tested. Silanes bearing alkyl, alkoxy, and siloxy groups were the source of silicon atoms. The results are shown in Table 3. For all of the substrates tested, nearly quantitative yields and exclusive formation of desired products was achieved. For the variety of substrates used, we did not observe a meaningful difference in the reaction course. Disappointingly, tris-(trimethylsiloxy)silane as well as di- and triphenylsilane are not suitable for our catalytic system. When we used these organosilicon compounds, their conversion was below 15%. The low efficiency of the process is probably related to large steric crowding at the silicon atom in the substrates, which hinders the access to the metal center. Moreover, in crowded silanes, the problem is the presence of the bulky NHC ligand that significantly screens the central atom, limiting the access of the reagents to the metal from all sides. We did not isolate all products because most of them are known. We isolated only selected compounds to develop a universal method for their separation from a reaction mixture.

**Table 3:** Formation of the Si–S Bond Catalyzed by **II**^a^^,^^b^

entry	silane 1a–g	thiol 2a–g	*t* [h]	product 3a–g	yield^c^ [%] (isol.)
1	1a	2a	4	3a–a	100 (93)
2	1b		9	3b–a	100 (97)
3	1c		6	3c–a	98
4	1d		10	3d–a	100
5	1e		16	3e–a	98 (96)
6	1a	2b	5	3a–b	100
7	1b		10	3b–b	100
8	1c		6	3c–b	100
9	1d		9	3d–b	96
10	1e		24	3e–b	99
11	1a	2c	5	3a–c	100
12	1b		9	3b–c	100
13	1c		8	3c–c	99
14	1d		12	3d–c	96
15	1e		24	3e–c	99
16	1a	2d	8	3a–d	100
17	1b		12	3b–d	98
18	1c		16	3c–d	96 (97)
19	1d		16	3d–d	100
20	1e		24	3e–d	98 (97)
21	1a	2e	3	3a–e	95
22	1b		8	3b–e	98 (94)
23	1c		5	3c–e	97
24	1d		9	3d–e	96
25	1e		10	3e–e	97 (92)
26	1a	2f	4	3a–f	99
27	1b		6	3b–f	100 (98)
28	1c		7	3c–f	100 (96)
29	1d		6	3d–f	98
30	1e		9	3e–f	97
31	1a	2g	5	3a–g	100
32	1b		7	3b–g	100
33	1c		9	3c–g	99
34	1d		12	3d–g	97
35	1e		24	3e–g	98

a Substrate scope.

b Reaction conditions: solvent-free, argon, 110 °C, **II** = [RhCl(cod)(IPr*^Ph^)], [**II**] = 1 mol %, and [silane]:[thiol] = 1:1.

c Determined by GC analyses.

**Scheme 4:**
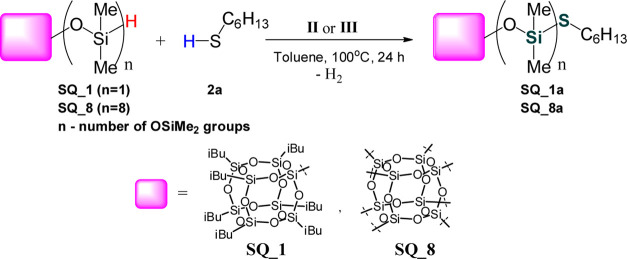
Coupling of 1-Hexanethiol (**2a**) with Monohydro-Substituted Spherosilicate (**SQ_1**) and Octahydro-Substituted Spherosilicate (**SQ_8**)

To highlight the utility of this procedure for the coupling between thiols and organosilicon compounds, we conducted experiments with the use of mono- and octahydro-substituted spherosilicates (Scheme 4). We turned our attention to functionalization of this kind of compounds because, according to our knowledge, there are no literature reports on the functionalization of silsesquioxanes (SQs) with thiols. Having in mind the unique properties of the materials based on SQs affecting the directions of their versatile applications,^26^ it seems desirable to broaden the coupling of thiols with SQ derivatives.

**Figure 1:**
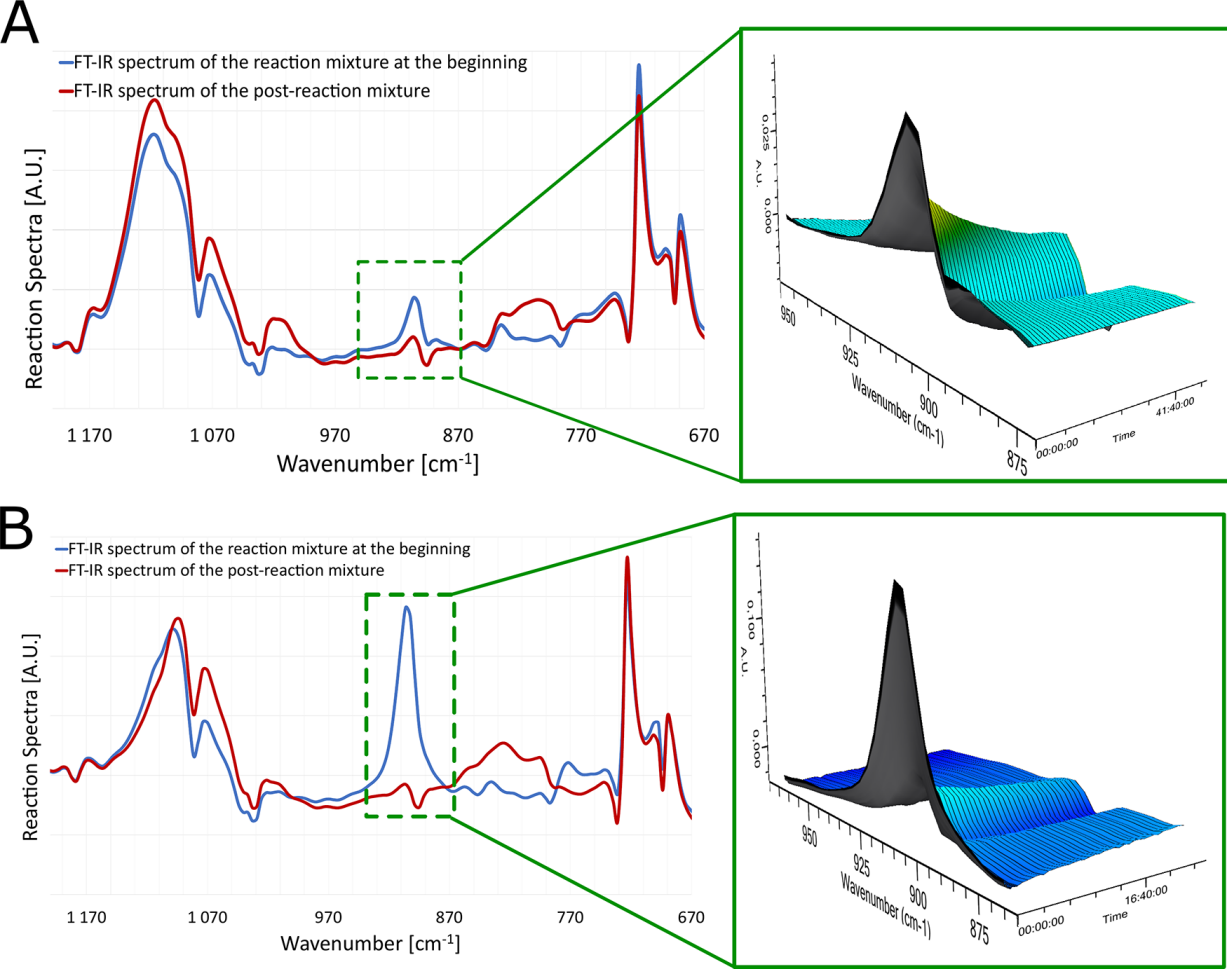
Disappearance of the Si–H bond recorded by real-time FT-IR during coupling of monospherosilicate (**SQ_1**) (A) and octahydro-spherosilicate (**SQ_8**) (B) with **2a**.

Catalytic tests were carried out according to the procedure described in the Supporting Information. Due to the large molecular weight of reactants, the reaction progress could not have been monitored by GC. We applied in situ Fourier transform infrared spectroscopy (FT-IR) in real time to monitor the consumption of the Si–H bond in SQs (Figure 1).

It was necessary to use a solvent to record the spectrum by the IR probe used in the experiment. In both cases, we observed complete disappearance of the Si–H bond after 24 h of the reaction, which proves the possibility of effective modification of SQs with thiols. Additionally, formation of expected products was confirmed by ^1^H nuclear magnetic resonance (NMR) analyses of the post-reaction mixtures.

### Formation of the S–S Bond

In the next step, complex **II** was tested in the oxidation of thiols to disulfides. The choice of this reaction resulted from our observations made during the study of thiol–silane coupling processes. In the tests conducted with the access of air, we observed the formation of significant amounts of disulfides and trace amounts of silylthioethers (Table 1, entry 10). In the reactions carried out under dry argon, the main products were silylthioethers, while no disulfide formation was observed. The results suggest that formation of the S–S bond requires the access of oxygen as then this process is favored over that of Si–S formation. It is in agreement with the mechanism of this process described earlier by Arisawa’s group, which assumes that the presence of oxygen is of key importance in the synthesis of disulfides.^24^ Taking this into account, it is highly probable that oxygen is necessary to provide a hydroperoxy moiety (OOH), which in the next step reacts with thiol to form the expected product and water.

The addition of 1 mol % complex **II** to **2a** at room temperature led to the formation of a single product in quantitative yield, which was identified by GC–MS and ^1^H NMR analysis as the expected di-*n*-hexyl disulfide. Moreover, the catalytic activities of **II** and **III** were compared to those of the selected rhodium complexes, including **I** and **IV**–**VI** (Table S2). Among all of the complexes tested, catalysts **II** and **III** were found to be the best ones in terms of activity. Using these catalysts, the quantitative yields of products were achieved even in the presence of 0.25 mol % complex **II** or **III**. The transformation of the substrate was also observed when the reaction was catalyzed by rhodium analogues bearing less-bulky NHC ligands but then the conversion of the substrate was incomplete. Complexes **I** and **IV** were found to be inactive as insignificant conversion of **2a** was observed even though the reaction time and amount of the catalysts were considerably increased. Furthermore, no conversion was observed under the conditions tested for the process without the rhodium catalyst. Increasing catalytic activity of the complexes containing bulky ligands stays in agreement with our earlier studies.^27^ We assumed that formation of the Rh–S bond occurs in all rhodium catalysts tested because the addition of thiols to the rhodium complexes is well known and relatively easy.^28^ However, application of bulky NHC carbene as an ancillary ligand seems pivotal for the effective transformation. It has been proved that the presence of bulky substituents localized at the nitrogen atoms in the NHC ring allows efficient product elimination and growing catalytic activity of such systems relative to the analogous ones containing simple NHC ligands (**V** and **VI**) or without NHC ligands (**I** and **VI**).

**Scheme 5:**
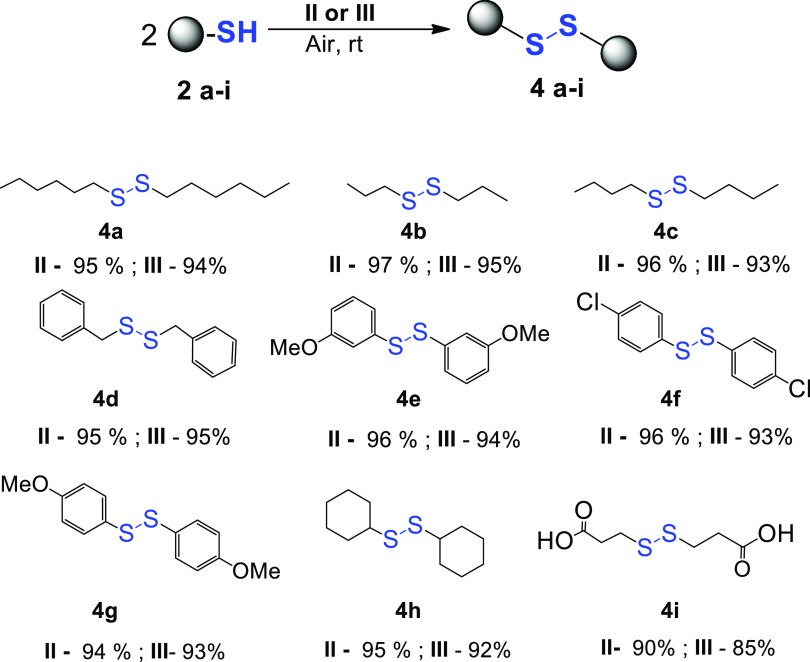
Oxidation of Thiols Catalyzed by **II** and **III** Reaction conditions: solvent-free, RT, air, and [**Rh**] = 0.25 mol %. For the reaction of **2d**, the catalyst loading was 1 mol %. For the process of **2f**, 100 μL of toluene was used. Yields of isolated products are given except **4b** and **4h**. Yields of **4b** and **4h** were determined by GC–MS analyses.

Having an active and selective catalyst in hand, the range of substrates was extended to determine the versatility of the method (Scheme 5).

For all thiols tested (**2a–i**), nearly quantitative yields of expected disulfides (**4a–i**) were observed. The course of reactions was independent of the type of catalyst used because complexes **II** and **III** showed similar catalytic activities. For the variety of substrates used, we did not observe meaningful differences in the reaction course. Only for the thiols containing carboxyl groups (**2i**), catalysts **II** and **III** had to be used in a higher concentration to achieve high yields.

**Scheme 6:**
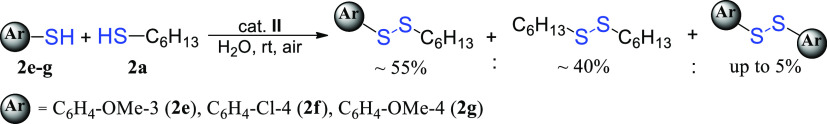
Formation of Unsymmetric Disulfides Catalyzed by **II**

In the optimized reaction systems, we have also checked the possibility of formation of asymmetric disulfides. Relevant tests were performed using two different thiols. Unfortunately, we were not successful in getting the expected product with full selectivity. Irrespective of the reagents used, we always obtained a mixture of symmetric and asymmetric disulfides. The best results were observed for the processes with aryl- and alkyl-substituted thiols used simultaneously. This choice of reagents significantly restricted the formation of a symmetric diaryl-substituted product (Scheme 6).

### Reusability of Catalysts **II** and **III** in S–Si and S–S Bond Forming Reactions

**Figure 2:**
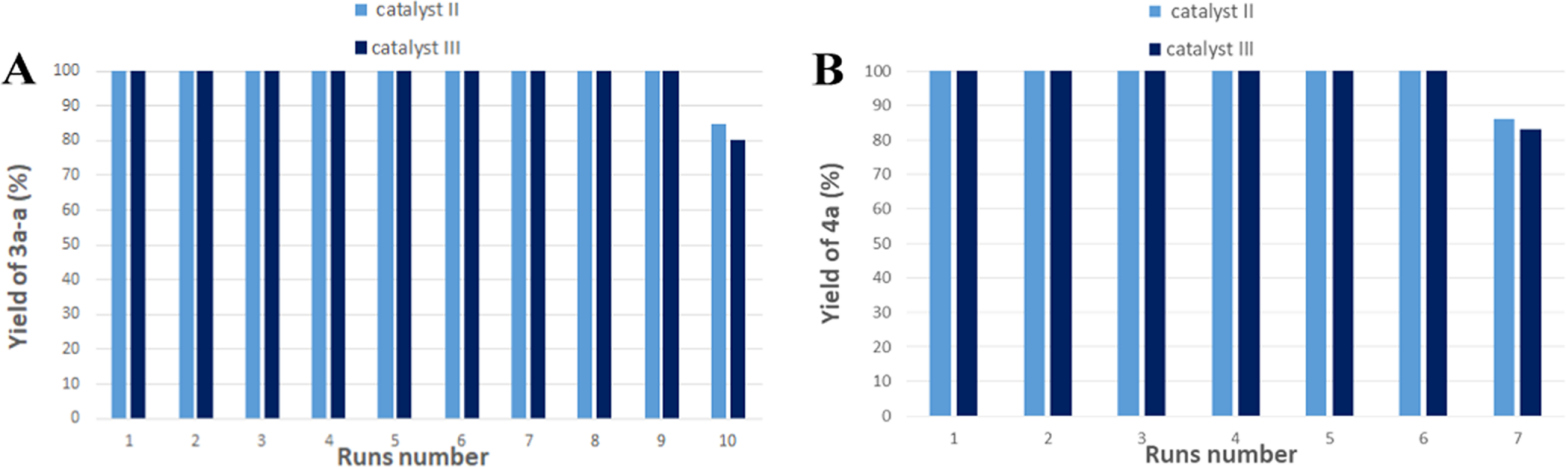
Recycling of catalysts **II** and **III** for coupling between **1a** and **2a** (A) and oxidation of **2a** (B).

Lastly, we examined the reusability of catalysts **II** and **III** in both processes tested. We performed separate recycling experiments involving the synthesis of triethoxy(hexylthio)silane (**3a–a**) and di-*n*-hexyl disulfide (**4a**). Catalytic tests were carried out under previously optimized reaction conditions and the progress of the reactions was monitored by GC analyses. After full conversion of substrates was detected, the next portion of thiol or thiol and silane was added to the reaction mixture. This procedure was repeated until a decrease of the catalyst activity was observed. In both processes for which reproducibility was investigated, catalysts **II** and **III** could be reused at least six or nine times, respectively, without loss of their catalytic activity (Figure 2). The high values of conversion in subsequent cycles testify to a very small progressive deactivation of the catalysts.

For both reactions catalyzed by **II** (i.e., oxidation of thiols and coupling between thiols and silanes), we also performed additional studies in which after each reaction, the catalyst was isolated from the reaction mixtures and the recovered catalysts were used in subsequent catalytic cycles. Recycling was made by removing the product with trap-to-trap distillation and recharging the residue. For both processes, we practically obtained the same results as for the tests carried out in the same pot but without recovering the catalyst.

## Conclusions

In summary, new NHC–rhodium complexes were synthesized, characterized, and proven to be catalytically active in the fully selective S–S and Si–S bond forming reactions. The results show many advantages from the point of view of the utility of the presented protocols in organic synthesis. These strategies feature high atom economy (hydrogen or water as the only byproduct, equimolar ratios of substrates, ease of product isolation), excellent yields, and wide substrate scope as well as the simplicity of the experimental techniques. The proposed methods allow obtaining both types of products in solvent-free conditions. However, these processes can also be carried out in water, toluene, or their mixture, which is an alternative when solid-state substrates are used. Moreover, the synthesis of disulfides is carried out in air, at room temperature. The design complexes exhibited good recyclability because we proved that they could be reused six or nine times without loss of their catalytic activity. In light of the results described above, the presented strategies are less harmful to the environment than those described so far in the literature. It is worth emphasizing that the methodology presented in this article opens up a possibility to obtain a new class of SQs with potential for practical applications.

## Supporting Information Available

Experimental details, NMR spectra, and other results (PDF)
