# Supplementary material for: Application of Bulky NHC–Rhodium Complexes in Efficient S–Si and S–S Bond Forming Reactions
Source: Inorg Chem. 2021 Nov 5;60(23):17579–85. doi: 10.1021/acs.inorgchem.1c02160 (PMC8653157; doi:10.1021/acs.inorgchem.1c02160)
Supplement: Supplementary file 1 — ic1c02160_si_001.pdf [file ic1c02160_si_001.pdf]

Application of bulky NHC-rhodium complexes in efficient S-Si and S-S bonds forming reactions

Małgorzata Bołt and Patrycja Żak*

Adam Mickiewicz University in Poznań, Faculty of Chemistry, Uniwersytetu Poznańskiego 8, 61-614 Poznań, Poland

1. General methods and chemicals S2
2. General procedure for the synthesis of rhodium complexes S2
3. General procedure for catalytic tests S3
4. General procedure for the synthesis of silylthioethers S3
5. General procedures for the synthesis of disulphides S3
6. The study of catalytic performance of catalyst **III** in a thiol-silane coupling reaction S4
7. Oxidation of thiols. Optimisation of the reaction conditions S5
8. The study of the reusability of catalysts **II** and **III** S5
9. Analytical data of NHC rhodium complexes S6
10. Analytical data of isolated products S7
    10.1. Silylthioethers S7
    10.2. Disulphides S10
11. NMR spectra of rhodium complexes S12
12. NMR spectra of isolated products S16
    12.1. NMR spectra of silylthioethers S16
    12.2. NMR spectra of disulphides S29
13. References S36

1. General methods and chemicals

All syntheses and catalytic tests (except for the oxidation of thiols to disulphides) were carried out under dry argon, using standard Schlenk-line and vacuum techniques. ^{1}H NMR and ^{13}C NMR spectra were recorded in $CDCl_3$ on a Varian 400 operating at 402.6 or 300.41 and 101.2 MHz, respectively. ^{29}Si NMR spectra were recorded on a Bruker Ascend 400 Nanobay operating at 79.50 MHz. GC analyses were carried out on an Agilent 7890B (column: DB-530 m I.D. 0.53 mm) equipped with TCD. The GC-MS analyses were performed on a Varian Saturn 2100T equipped with (DB-1, 30 m capillary column, I.D. 0.25 mm) and Ion Trap Detector. Thin layer chromatography (TLC) was conducted on plates coated with a 250 mm thick silica gel layer and column chromatography was performed on silica gel 60 (70–230 mesh). High resolution mass spectroscopic analyses were performed using Synapt G2-S HDMS (Waters) mass spectrometer equipped with the Electrospray ion source and a quadrupole-Time-of-flight mass analyzer with the resolving power FWHM 38000 using methanol as a solvent. The Capillary Voltage was set to 4.5 kV, the sampling was set 40 and the source temperature was 120°C. In the ESI-MS spectra the sodiated ion was the most abundant. Real-time FT-IR measurements were performed on a Mettler Toledo ReactIR 15 equipped with a DS 6.3 mm AgXDiComp Fiber Probe with a diamond sensor, and a Mercury Cadmium Telluride detector. For all the spectra 256 scans were recorded with the resolution of 1 cm-1 in 1, 5 and 10 min intervals.

All reagents were commercially available and used as received. 1,3-bis{2,6-bis(diphenylmethyl)-4-ethylphenyl}imidazolium chloride, 1,3-bis{2,4,6-tris(diphenyl-methyl)phenyl}imidazolium chloride, 1,3-bis(2,4,6-trimethylphenyl)imidazolium tetra-fluoroborate and 1,3-bis(2,6-diisopropylphenyl)imidazolium tetrafluoroborate were prepared according to the literature procedures.[1] All solvents (except THF) were dried prior to use over CaH_2 and stored under argon over 4Å molecular sieves. DCM was additionally passed through a column with alumina and after that it was degassed by repeated freeze-pump-thaw cycle. THF was dried over sodium benzophenone ketyl and freshly distilled prior to use.

2. General procedure for the synthesis of rhodium complexes

A 25 mL high-pressure Schlenk vessel equipped with a magnetic stirring bar and connected to gas and vacuum line was charged with potassium *tert*-butoxide (23.6 mg, 2.10×10^{-4} mol, 2.1 eq.) and 8 mL of dry THF. The reaction mixture was cooled in an ice bath and chloro(1,5-cyclooctadiene)rhodium(I) dimer (49.3 mg, 10^{-4} mol, 1eq.) was introduced. The resulting orange solution was stirred for 15 min in an ice bath and then for 45 min at room temperature. After this time, imidazolium salt (2.10×10^{-4} mol, 2.1 eq.) was added and the reaction mixture evolved progressively from orange to yellow. After 7h, the solution was filtered on a pad of Celite and eluted with dichloromethane. The solvents were evaporated under vacuum and the yellow residue was purified using

column chromatography (silica gel 60/DCM:EtOAc = 1:1). Evaporation of the solvent gave the analytically pure rhodium(I) complex as yellow solid.

3. General procedure for catalytic tests

Products 3a-g: The oven-dried 5 mL glass reactor equipped with a reflux condenser and a magnetic stirring bar was charged under argon with thiol ($2.10{\times}10^{-4}$ mol), silane ($2.10{\times}10^{-4}$ mol) and internal standard (decane or dodecane, 20 µL). The reaction mixture was warmed up in an oil bath to 110 °C and rhodium complex **II** or **III** ($2.10{\times}10^{-6}$ mol) was added to the mixture. The mixture was heated for 24 h. Conversion of the substrate was monitored by gas chromatography (GC).

Products 4a-i: A 2 ml glass vial equipped with a magnetic stirring bar was charged with thiol ($3.5{\times}10^{-4}$ mol) and internal standard (decane or dodecane, 20 µL). Then, rhodium complex **II** or **III** ($8.8{\times}10^{-7}$ mol) was added and the reaction mixture was stirred at RT for 24 h or until full conversion of S-H was detected. Reaction course was monitored by gas chromatography.

Products SQ_1a, SQ_8a: The oven-dried 5 mL glass reactor was charged under argon with dry toluene (0.5 mL), thiol ($2.10{\times}10^{-4}$ mol) and mono- (**SQ-1**, $2.10{\times}10^{-4}$ mol) or octa-spherosilicates (**SQ_8**, $2.62{\times}10^{-5}$ mol). Then the system was equipped with an *in situ* FT-IR probe. The reaction mixture was warmed up in an oil bath to 100 °C and rhodium complex **II** ($2.10{\times}10^{-6}$ mol) was added. The mixture was heated for 24 h. Conversion of the substrate was monitored by *in situ* FT-IR in real-time.

4. General procedure for the synthesis of silylthioethers (3a-g)

A flame-dried glass reactor, equipped with a reflux condenser and a magnetic stirring bar, was charged with argon, thiol (2 mmol) and silane (2 mmol). Additionally, when using 4-chlorothiophenol, dry toluene (100 µL) was added. Then, the reaction mixture was heated to 110 °C in an oil bath and rhodium complex **II** or **III** ($2{\times}10^{-5}$ mol) was added. After 24 h, analytically pure product was isolated by trap-to-trap distillation of the reaction mixture.

5. General procedures for the synthesis of disulphides (4a-g)

A 2 ml glass vial equipped with a magnetic stirring bar was charged with thiol (2 mmol) and rhodium complex **II** or ($2.5{\times}10^{-6}$ mol). Additionally, in case of 4-chlorothiophenol dry toluene (100 µL) was added. The reaction mixture was stirred at RT for 24 h or until full conversion of S-H was detected (GC). After this time, the resulting product was isolated by trap-to-trap distillation of the reaction mixture or by column chromatography (silica gel 60, *n*-hexane or *n*-hexane/DCM = 10:1). Evaporation of the solvent gave an analytically pure sample.

6. The study of catalytic performance of catalyst III in a thiol-silane coupling reaction

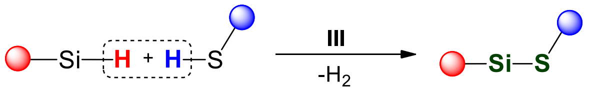

1a-g **2a-g** **3 a-g**

- (OEt_3) (**1a**), *t*$BuMe_2$ (**1b**), Et_3 (**1c**), $(OEt)_2Me$ (**1d**), $(OSiMe_3)_2Me$ (**1e**)

- C_6H_{13} (**2a**), C_3H_7 (**2b**), C_5H_{11} (**2c**), C_6H_5-CH_2 (**2d**), C_6H_4-OMe-3 (**2e**), C_6H_4-Cl-4 (**2f**), C_6H_4-OMe-4 (**2g**)

Entry	Silane 1 a-g	Thiol 2 a-g	t [h]	Product 3 a-g	Yield[a] [%] (Isolated yield)
1	1a		7	3 a-a	99 (94)
2	1b		8	3 b-a	98 (92)
3	1c	2a	6	3 c-a	97
4	1d		12	3 d-a	98
5	1e		12	3 e-a	96 (93)
6	1a		4	3 a-b	96
7	1b		9	3 b-b	99
8	1c	2b	5	3 c-b	98
9	1d		9	3 d-b	96
10	1e		24	3 e-b	99
6	1a		5	3 a-c	99
7	1b		10	3 b-c	98
8	1c	2c	10	3 c-c	99
9	1d		12	3 d-c	96
10	1e		24	3 e-c	99
11	1a		7	3 a-d	100
12	1b		10	3 b-d	99
13	1c	2d	16	3 c-d	94 (90)
14	1d		16	3 d-d	100
15	1e		24	3 e-d	98 (95)
16	1a		3	3 a-e	99
17	1b		8	3 b-e	97 (95)
18	1c	2e	5	3 c-e	99
19	1d		9	3 d-e	97
20	1e		10	3 e-e	97 (93)
21	1a		4	3 a-f	99
22	1b		6	3 b-f	99 (94)
23	1c	2f	7	3 c-f	98 (93)
24	1d		6	3 d-f	98
25	1e		9	3 e-f	97
26	1a		5	3 a-g	100
27	1b		7	3 b-g	98
28	1c		9	3 c-g	99
29	1d		12	3 d-g	98
30	1e	2g	24	3 e-g	95

Reaction conditions: [silane]:[thiol] = 1:1, **III** = [RhCl(cod)(IPr^{*Et})], [**III**] = 1 mol%, argon; [a] Determined by GC and GC-MS analyses.

7. Oxidation of thiols. Optimisation of reaction conditions.

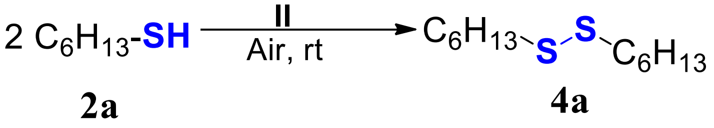

Optimization of the type and loading of catalyst.

Entry	Cat.	Cat. [mol %]	t [h]	Yield[a] [%]
1	$[Rh(cod)Cl]_2$	3	72	11
2	[RhCl(cod)(IPr*^{Ph})]	1	2	100
3	[RhCl(cod)(IPr*^{Ph})]	1	2	100
**4**	[RhCl(cod)(IPr*^{Ph})]	**0.25**	**8**	**100**
5	[RhCl(cod)(IPr*^{Ph})]	0.1	24	98
6	[RhCl(cod)(IPr*^{Ph})]	0.01	24	72
7	[RhCl(cod)(IPr*^{Et})]	1	2	100
**8**	[RhCl(cod)(IPr*^{Et})]	**0.25**	**12**	**100**
9	$RhCl_3 \times 3H_2O$	3	72	10
10	[RhCl(cod)(IMes)]	1	24	54
11	[RhCl(cod)(IPr)]	1	24	55
12	-	-	72	0

Solvent-free, RT, air, [a] Determined by GC and GC-MS analyses.

8. The study of the reusability of catalyst II and III

8.1. Coupling between thiol and silane

A flame-dried glass reactor equipped with a reflux condenser and magnetic stirring bar was charged under argon with 1-hexanethiol **2a** (40 µL, 2.81×10^{-4} mol), triethoxysilane **1a** (52 µL, 2.81×10^{-4} mol) and dodecane (20 µL). The reaction mixture was warmed up in an oil bath to 110°C and rhodium complex **II** or **III** (2.81×10^{-6} mol) was added. The progress of the reaction was monitored by gas chromatography (GC) and the reaction mixture was heated until complete conversion of substrates was detected. Then the next portion of substrates (**1a** and **2a**) was added and the process was carried out to achieve a total conversion of substrate again. The above procedure was repeated seven times.

Based on the catalyst reusability tests the overall TON of catalysts is 9×10^2. TON in one cycle is equal to 1×10^2. Turnover frequency (TOF) in one cycle is 6.00×10^{-2} s^{-1}, which stays in agreement with results obtained in optimization reactions.

8.2. Oxidation of 1-hexanethiol

A 2 ml glass vial equipped with a magnetic stirring bar was charged with 1-hexanethiol **2a** (40 µL, 2.81×10^{-4} mol), decane (20 µL) and rhodium complex **II** or **III** (2.81×10^{-6} mol). The reaction mixture was stirred at RT until complete conversion of substrates was detected. The progress of process was monitored by gas chromatography (GC). Then the next portion of thiol (**2a**) was added and the reaction was carried out to achieve a total conversion of substrate again. The above procedure was repeated seven times.
Based on the catalyst reusability tests the overall TON of catalysts is 6×10^2. TON in one cycle is equal to 1×10^2. Turnover frequency (TOF) in one cycle is 1.38×10^{-2} s^{-1}, which stays in agreement with results obtained in optimization reactions.

9. Analytical data of NHC rhodium complexes

Chloro(η^4-1,5-cyclooctadiene)(1,3-bis(2,4,6-tris(diphenylmethyl)phenylimidazol-2-ylidene)-rhodium(I), [RhCl(cod)(IPr*^{Ph2})] (**II**): Yellow solid, isolated yield: 91%, 266.4 mg (1.82×10^{-4} mmol); ^{1}H NMR ($CDCl_3$, δ, ppm): 1.44-1.56 (m, 2H, H_{COD}), 1.62 – 1.78 (m, 4H, H_{COD}), 1.94 – 2.10 (m, 2H, H_{COD}), 3.55 (br, 2H, H_{COD}), 4.84 (s, 2H, $CH^{4.5}$Im), 4.96 – 5.03 (br, 2H, H_{COD}), 5.19 (s, 2H, $CHAr_3$), 5.46 (s, 2H, $CHAr_3$), 6.62 – 6.73 (m, 6H, CH_{Ar}), 6.87 – 7.13 (m, 46H, CH_{Ar}), 7.15 – 7.21 (m, 10H, CH_{Ar}), 7.36 – 7.44 (m, 4H, CH_{Ar}); ^{13}C NMR ($CDCl_3$, δ, ppm): 28.44, 32.36, 50.84, 51.53, 56.33, 68.64 (d, $J_{Rh\text{-}C}$ = 14.4 Hz), 96.65 (d, $J_{Rh\text{-}C}$ = 8.0 Hz), 123.54, 125.67, 126.18, 126.33, 127.50, 127.88, 128.17, 129.09, 129.63, 129.90, 130.62, 131.33, 136.56, 143.45, 143.78, 144.38, 144.76, 184.20 (d, $J_{Rh\text{-}C}$ = 51.9 Hz); MS (ESI): calcd. for $C_{101}H_{85}N_2Rh$: 1428.5768; found: 1428.5724.

Chloro(η^4-1,5-cyclooctadiene)(1,3-bis(4-ethyl-2,6-bis(diphenylmethyl)phenylimidazol-2-ylidene)rhodium(I), [RhCl(cod)(IPr*^{Et})] (**III**): Yellow solid, isolated yield: 89%, 211.3 mg (1.78×10^{-4} mmol); ^{1}H NMR ($CDCl_3$, δ, ppm): 1.09 (t, 6H, J_{HH} = 7.6 Hz, CH_2CH_3), 1.44-1.55 (m, 2H, H_{COD}), 1.64 – 1.79 (m, 4H, H_{COD}), 1.97 – 2.08 (m, 2H, H_{COD}), 2.56 (q, 4H, J_{HH} = 7.5 Hz, CH_2CH_3), 3.58 (d, 2H, J_{HH} = 2.4 Hz, H_{COD}), 4.74 (s, 2H, $CH^{4.5}$Im), 4.92 – 5.01 (br, 2H, H_{COD}), 5.27 (s, 2H, $CHAr_3$), 6.69 (d, 4H, J_{HH} = 16.0 Hz, CH_{Ar}), 6.84 (d, 4H, J_{HH} = 16.1 Hz, CH_{Ar}), 6.91 – 7.30 (m, 27H, CH_{Ar}), 7.47 – 7.57 (m, 4H, CH_{Ar}); ^{13}C NMR ($CDCl_3$, δ, ppm): 15.57, 28.38, 28.77, 30.86, 32.44, 50.82, 51.53, 68.91 (d, $J_{Rh\text{-}C}$ = 14.0 Hz), 78.70, 96.52 (d, $J_{Rh\text{-}C}$ = 7.3 Hz), 123.54, 125.77, 126.40, 127.59, 127.86, 128.01, 129.16, 129.67, 130.15, 130.89, 136.33, 140.50, 143.20, 144.16, 144.31, 144.88, 183.84 (d, $J_{Rh\text{-}C}$ = 51.6 Hz); MS (ESI): calcd. for $C_{79}H_{73}N_2Rh$: 1152.4829; found: 1152.4778.

Chloro(η^4-1,5-cyclooctadiene)(1,3-dimesitylimidazol-2-ylidene)rhodium(I), [RhCl(cod)(IMes)] (**IV**): Yellow solid, isolated yield: 90%, 99.2 mg (1.80×10^{-4} mmol); ^{1}H NMR ($CDCl_3$, δ, ppm): 1.49-1.56 (m, 4H, H_{COD}), 1.78 – 1.91 (m, 4H, H_{COD}), 2.11 (s, 6H, CH_3), 2.30 – 2.45 (m, 12H, CH_3), 3.26 – 3.32 (br, 2H, H_{COD}), 4.48 – 4.56 (br, 2H, H_{COD}), 6.96 (s, 2H, $CH^{4.5}$Im), 6.98 – 7.06 (m, 4H, CH_{Ar}); ^{13}C NMR ($CDCl_3$, δ, ppm): 18.12, 19.79, 21.13, 28.37, 32.69, 67.80 (d, $J_{Rh\text{-}C}$ = 14.5 Hz), 96.08 (d,

J_{Rh-C} = 7.5 Hz), 123.48, 128.08, 129.66, 134.32, 136.21, 137.56, 138.65, 183.46 (d, J_{Rh-C} = 52.4 Hz); MS (ESI): calcd. for $C_{29}H_{36}ClN_2Rh$: 515.1928; found: 515.1928

Chloro(η⁴-1,5-cyclooctadiene)(1,3-bis(2,6-diisopropylphenylimidazol)-2-ylidene)rhodium(I), [RhCl(cod)(IPr)] (**V**)**:** Yellow solid, isolated yield: 92%, 116.9 mg (1.84×10^{-4} mmol); ^{1}H NMR (CDCl$_3$, δ, ppm): 1.09 (d, 12H, J_{HH} = 6.8 Hz, CH(CH_3)$_2$), 1.23 – 1.86 (m, 20H, H_{COD} and CH(CH_3)$_2$), 2.40 – 2.59 (br, 2H, CH(CH$_3$)$_2$), 3.20 – 3.28 (m, 2H, H_{COD}), 3.49 – 3.70 (br, 2H, CH(CH$_3$)$_2$), 4.51 – 4.61 (br, 2H, H_{COD}), 7.02 (s, 2H, C$H^{4.5}$Im), 7.26 – 7.46 (br, 4H, CH_{Ar}), 7.50 (t, 2H, J_{HH} = 7.7 Hz, CH_{Ar}); ^{13}C NMR (CDCl$_3$, δ, ppm): 26.54, 28.22, 28.79, 32.61, 67.76 (d, J_{Rh-C} = 14.3 Hz), 95.89 (d, J_{Rh-C} = 7.6 Hz), 122.84, 124.53, 129.74, 136.30, 185.97 (d, J_{Rh-C} = 52.0 Hz); MS (ESI): calcd. for $C_{35}H_{48}ClN_2Rh$: 599.2867; found: 599.2868

10. Analytical data of isolated products

10.1. Silylthioethers

3 a-a: Triethoxy(hexylthio)silane

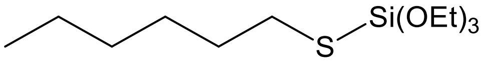

Colourless liquid, isolated yield: 93%, 521 mg; ^{1}H NMR (CDCl$_3$, δ, ppm): 0.88 (t, 3H, J_{HH} = 6.9 Hz, CH_3), 1.20-1.42 (m, 15H, CH_2), 1.57-1.66 (m, 2H, CH_2), 2.55-2.61 (m, 2H, CH_2), 3.88 (q, 6H, J_{HH} = 7.0 Hz, CH_3CH$_2$); ^{13}C NMR (CDCl$_3$, δ, ppm): 13.99 (CH$_3$), 17.97 (CH$_3$), 22.51 (CH$_2$), 26.12 (CH$_2$), 28.33 (CH$_2$), 31.28 (CH$_2$), 32.67 (CH$_2$), 59.32 (CH$_2$); ^{29}Si NMR (CDCl$_3$, δ, ppm): -52.46; MS m/z (rel. intensity): 55.00 (21), 56.90 (24), 83.00 (13), 85.00 (31), 117.00 (100), 149.90 (28), 234.10 (84), 235.10 (23), 281.20 (1).

3 b-a: (*Tert*-buthyl)(dimethyl)(hexylthio)silane

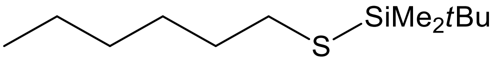

Colourless liquid, isolated yield: 97%, 451 mg; ^{1}H NMR (CDCl$_3$, δ, ppm): 0.24 (s, 6H, Si(CH_3)$_2$), 0.87 (t, 3H, J_{HH} = 7.0 Hz, -CH_3) 0.94 (s, 9H, C(CH_3)$_3$), 1.22-1.41 (m, 6H, CH_2), 1.54-1.62 (m, 2H, CH_2), 2.50-2.52 (m, 2H, CH_2); ^{13}C NMR (CDCl$_3$, δ, ppm): -3.64 (C(CH$_3$)$_3$), 14.01, 18.90 (Si(CH$_3$)$_2$), 22.54, 26.32, 26.53, 28.49, 31.38, 33.13; ^{29}Si NMR (CDCl$_3$, δ, ppm): 23.11; MS m/z (rel. intensity): 55.00 (14), 57.10 (16), 85.10 (15), 117.00 (72), 150.10 (12), 234.20 (100).

3 e-a: (Hexylthio)(methyl)bis(trimethylsiloxy)silane

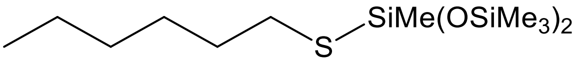

Colourless liquid, isolated yield: 96%, 650 mg; ^{1}H NMR ($CDCl_3$, δ, ppm): 0.13 (s, 18H, ($\mathrm{OSiC}H_3$), 0.29 (s, 3H $\mathrm{SiC}H_3$), 0.88 (t, 3H, J_{HH} = 7.0 Hz, -$\mathrm{C}H_3$), 1.23-1.41 (m, 6H, $\mathrm{C}H_2$), 1.54-1.63 (m, 2H, $\mathrm{C}H_2$), 2.46-2.53 (m, 2H, $\mathrm{C}H_2$);^{13}C NMR ($CDCl_3$, δ, ppm): 1.67 ($\mathrm{OSi}C\mathrm{H_3}$), 1.95, 14.01, 22.56, 26.25, 28.51, 31.39, 32.85; ^{29}Si NMR ($CDCl_3$, δ, ppm): 9.66 ($\mathrm{O}Si\mathrm{Me_3}$), -30.96; MS m/z (rel. intensity): 73.30 (15), 21.80 (93), 220.80 (31), 222.70 (48), 223.70 (30), 323.550 (100), 324.50 (27), 325.50 (19).

3 c-d: (Benzylthio)triethylsilane

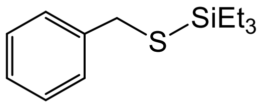

Colourless liquid, isolated yield: 97%, 462 mg; ^{1}H NMR ($CDCl_3$, δ, ppm): 0.65 (q, 6H, J_{HH} = 7.9 Hz, $\mathrm{C}H_3\mathrm{CH_2}$), 0.91 (t, 9H, J_{HH} = 7.9 Hz, $\mathrm{CH_3C}H_2$), 3.60 (s, 2H, $\mathrm{C_6H_5\text{-}C}H_2$-), 7.09-7.15 (m, 1H, $\mathrm{C_6}H_5$-), 7.16-7.25 (m, 4H, $\mathrm{C_6}H_5$-); ^{13}C NMR ($CDCl_3$, δ, ppm): 5.21 ($C\mathrm{H_3}$), 7.18 ($C\mathrm{H_2}$), 29.66 ($C\mathrm{H_2}$), 126.67, 128.32, 140.39; ^{29}Si NMR ($CDCl_3$, δ, ppm): 23.47; MS m/z (rel. intensity): 65.10 (12), 91.10 (92), 179.30 (22), 181.20 (13), 209.20 (100), 210.20 (17), 239.10 (16).

3 e-d: (Benzylthio)(methyl)bis(trimetylosiloxy)silane

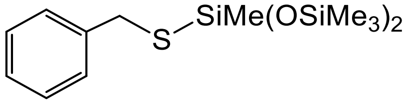

Colourless liquid, isolated yield: 97%, 668 mg; ^{1}H NMR ($CDCl_3$, δ, ppm): 0.17 (s, 18H, $\mathrm{OSi(C}H_3)_3$), 0.30 (s, 3H, $\mathrm{SiC}H_3$), 3.78 (s, 2H, -$\mathrm{C}H_2$-), 7.21-7.25 (m, 1H, $\mathrm{C_6}H_5$-), 7.28-7.37 (m, 4H, $\mathrm{C_6}H_4$-); ^{13}C NMR ($CDCl_3$, δ, ppm): 1.68 ($\mathrm{OSi}C\mathrm{H_3}$), 2.08 ($C\mathrm{H_3}$), 30.34 ($C\mathrm{H_2}$), 126.71, 128.41 (d, J = 9.1 Hz), 140.47; ^{29}Si NMR ($CDCl_3$, δ, ppm): 10.21 ($\mathrm{O}Si\mathrm{Me_3}$), -31.52; MS m/z (rel. intensity): 73.20 (11), 91.20 (35), 221.50 (100), 223.20 (18), 329.10 (7), 343.80 (7, M^+).

3 b-e: (*Tert*-buthyl)(dimethyl)(3-methoxyphenylthio)silane

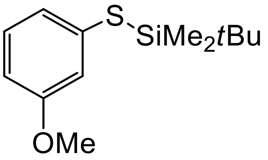

Colourless liquid, isolated yield: 94%, 478 mg; ^{1}H NMR ($CDCl_3$, δ, ppm): 0.20 (s, 6H $\mathrm{Si(C}H_3)_2$), 0.98 (s, 9H, $\mathrm{C(C}H_3)_3$), 3.39 (s, 3H, $\mathrm{OC}H_3$), 6.76-6.80 (s, 1H, -$\mathrm{C_6}H_4\mathrm{\text{-}OCH_3}$), 6.98-7.04 (m, 2H, -$\mathrm{C_6}H_4\mathrm{\text{-}OCH_3}$), 7.12-7.18 (m, 1H, -$\mathrm{C_6}H_4\mathrm{\text{-}OCH_3}$); ^{13}C NMR ($CDCl_3$, δ, ppm): -3.24 ($C\mathrm{(CH_3)_3}$), 18.93 ($\mathrm{Si(CH_3)_2}$), 26.40 ($\mathrm{C(C}H_3)_3$), 55.20 ($\mathrm{OC}H_3$), 112.80, 120.69, 127.88, 129.26, 132.48, 159.36;

^{29}Si NMR ($CDCl_3$, δ, ppm): 25.15; MS m/z (rel. intensity): 73.10 (21), 166.90 (20), 182.00 (75), 183.00 (21), 197.20 (100), 198.10 (19), 239.00 (11), 253.70 (24, M^+).

3 e-e: (3-methoxyphenylthio)(methyl)bis(trimethylsiloxy)silane

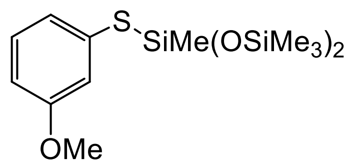

Colourless liquid, isolated yield: 92%, 664 mg; ^{1}H NMR ($CDCl_3$, δ, ppm): 0.11 (s, 18H, OSiCH_3), 0.29 (s, 3H, SiCH_3), 3.79 (s, 3H, OCH_3), 6.75-6.79 (m, 1H, -C_6H_4-OCH_3), 7.02-7.06 (m, 2H, -C_6H_4-OCH_3), 7.12-7.17 (m, 1H, -C_6H_4-OCH_3); ^{13}C NMR ($CDCl_3$, δ, ppm): 1.58 (OSiCH_3), 55.18 (OCH_3), 112.62, 119.71, 126.78, 129.29, 131.98, 159.33; ^{29}Si NMR ($CDCl_3$, δ, ppm): 10.50 (O*Si*Me_3), -34.52; MS m/z (rel. intensity): 45.10 (11), 73.30 (45), 133.10 (7), 221.60 (100), 222.50 (40), 223.30 (23), 345.60 (48), 346.40 (15), 360.20 (70, M^+).

3 b-f: (*Tert*-buthyl)(dimethyl) (4-chlorophenylthio)silane

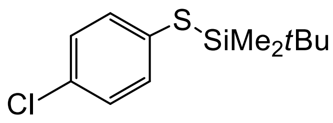

Colourless liquid, isolated yield: 98%, 507 mg; ^{1}H NMR ($CDCl_3$, δ, ppm): 0.17 (s, 6H, SiCH_3), 0.97 (s, 9H, C(CH_3)$_3$), 7.19-7.21 (m, 2H, -C_6H_4-Cl), 7.34-7.37 (m, 2H, -C_6H_4-Cl); ^{13}C NMR ($CDCl_3$, δ, ppm): -3.33 ($C(CH_3)_3$), 18.94 ($Si(CH_3)_2$), 26.37 ($C(CH_3)_3$), 128.76, 129.13, 130.13, 130.75, 133.07; ^{29}Si NMR ($CDCl_3$, δ, ppm): 25.51; MS m/z (rel. intensity): 45.00 (11), 73.20 (70), 91.20 (12), 93.00 (16), 136.90 (11), 165.20 (85), 201.20 (100), 203.10 (45), 205.10 (10), 243.20 (24), 257.80 (48), 258.80 (12, M^+).

3 c-f: (4-chlorobenzylthiol)triethylsilane

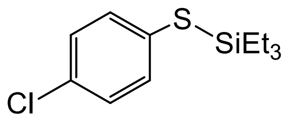

Colourless liquid, isolated yield: 96%, 497 mg; ^{1}H NMR ($CDCl_3$, δ, ppm): 1.18 (t, 9H, J_{HH} = 7.0 Hz, CH_3CH_2), 3.85 (t, 6H, J_{HH} = 7.0 Hz, CH$_3$CH_2), 7.20 (d, 2H, J_{HH} = 8.8 Hz, -C_6H_4-), 7.42 (d, 2H, J_{HH} = 8.4 Hz, -C_6H_4-); ^{13}C NMR ($CDCl_3$, δ, ppm): 17.80 (CH_3), 59.72 (CH_2), 127.85, 128.85, 129.08, 130.70, 132.88, 134.87; ^{29}Si NMR ($CDCl_3$, δ, ppm): -58.81; MS m/z (rel. intensity): 119.10 (10), 163.30 (12), 253.30 (14), 297.50 (20), 306.20 (100, M^+).

10.2.Disulphides

4a: Di-*n*-hexyl disulphide

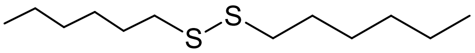

Colourless liquid, isolated yield: 95%, 445 mg; ^{1}H NMR ($CDCl_3$, δ, ppm): 0.89 (t, 6H, J_{HH} = 6.9 Hz, CH_3), 1.24 – 1.43 (m, 12H, CH_2), 1.63 – 1.71 (m, 4H, CH_2), 2.53 – 2.86 (m, 4H, CH_2); ^{13}C NMR ($CDCl_3$, δ, ppm): 14.03 (CH_3), 22.53 (CH_2), 28.19 (CH_2), 29.17 (CH_2), 31.42 (CH_2), 39.18 (CH_2); MS m/z (rel. intensity): 117.20 (49), 233.50 (14), 234.50 (100, M^+).

4b: Dipropyl disulphide

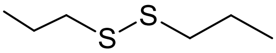

Colourless liquid, isolated yield: 97%, 291 mg; ^{1}H NMR ($CDCl_3$, δ, ppm): 0.98 (t, 6H, J_{HH} = 7.3 Hz, $CH_3CH_2CH_2$), 1.64 – 1.74 (m, 4H, $CH_3CH_2CH_2$), 2.63 – 2.68 (m, 4H, $CH_3CH_2CH_2$); ^{13}C NMR ($CDCl_3$, δ, ppm): 13.07 (CH_3), 22.44 (CH_2), 41.07 (CH_2); MS m/z (rel. intensity): 149 (16), 150 (100, M^+).

4d: Dibenzyl disulphide

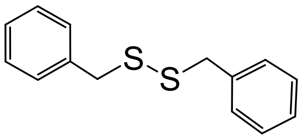

Colourless liquid, isolated yield: 95%, 474 mg; ^{1}H NMR ($CDCl_3$, δ, ppm): 3.60 (s, 4H, CH_2), 7.21 – 7.26 (m, 4H, C_6H_5-), 7.26 – 7.36 (m, 6H, C_6H_5-); ^{13}C NMR ($CDCl_3$, δ, ppm): 43.21 (CH_2), 127.39, 128.44, 129.38, 137.31; MS m/z (rel. intensity): 45.20 (10), 65.00 (13), 91.20 (100), 92.20 (11), 181.20 (35), 245.90 (22), 246.80 (16, M^+).

4e: Bis(3-methoxyphenyl) disulphide

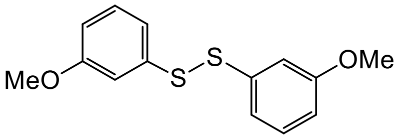

White solid, isolated yield: 96%, 534 mg; ^{1}H NMR ($CDCl_3$, δ, ppm): 3.77(s, 6H, OCH_3), 6.72 – 6.78 (m, 2H, -C_6H_4-OCH_3), 7.05 – 7.11 (m, 4H, -C_6H_4-OCH_3), 7.17 – 7.24 (m, 2H, -C_6H_4-OCH_3); ^{13}C NMR ($CDCl_3$, δ, ppm): 55.27 (OCH_3), 112.48, 113.07, 119.51, 129.87, 138.23, 159.99;

MS m/z (rel. intensity): 95.20 (15), 124.20 (10), 139.20 (7), 214.50 (13), 230.30 (16), 245.50 (19), 278.20 (100, M^+).

4f: Bis(4-chlorophenyl) disulphide

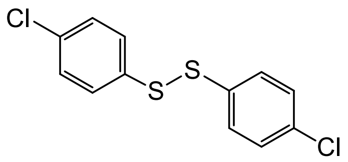

White solid, isolated yield: 96%, 552 mg; ^{1}H NMR ($CDCl_3$, δ, ppm): 7.18 (m, 4H, -C_6H_4-Cl), 7.29 – 7.34 (m, 4H, -C_6H_4-Cl); ^{13}C NMR ($CDCl_3$, δ, ppm): 129.25, 133.57, 135.07; MS m/z (rel. intensity): 108.20 (44), 143.10 (64), 144.00 (14), 145.00 (26), 286 (100, M^+).

4h: Dicyclohexyl disulphide

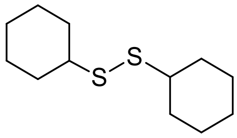

Colourless liquid, isolated yield: 95%, 438 mg; ^{1}H NMR ($CDCl_3$, δ, ppm): 1.07 – 1.42 (m, 11H, Cy), 1.54 – 1.64 (m, 2H, Cy), 1.71 – 1.83 (m, 4H, Cy), 1.96 – 2.09 (m, 4H, Cy), 2.56 – 2.77 (m, 2H, Cy); ^{13}C NMR ($CDCl_3$, δ, ppm): 25.65 (CH_2), 26.04 (CH_2), 32.79 (CH_2), 49.87 (CH); MS m/z (rel. intensity): 55.00 (5), 113.00 (7), 230 (100, M^+).

4i: Bis(2-carboxylethyl) disulphide

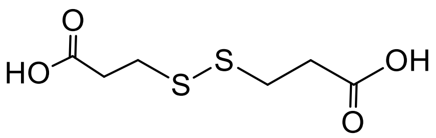

White solid, isolated yield: 90%, 404 mg; ^{1}H NMR (DMSO-d_6, δ, ppm): 2.62 (t, 2H, J_{HH} = 6.9 Hz, CH_2), 2.88 (t, 2H, J_{HH} = 6.9 Hz, CH_2), 12.41 (br s, 1H, OH); ^{13}C NMR (DMSO-d_6, δ, ppm): 33.01, 33.60, 172.76; MS m/z (rel. intensity): 51.10 (9) 65.00 (9), 77.10 (10), 91.90 (100), 105.90 (54), 210.90 (2).

11. NMR spectra of rhodium complexes

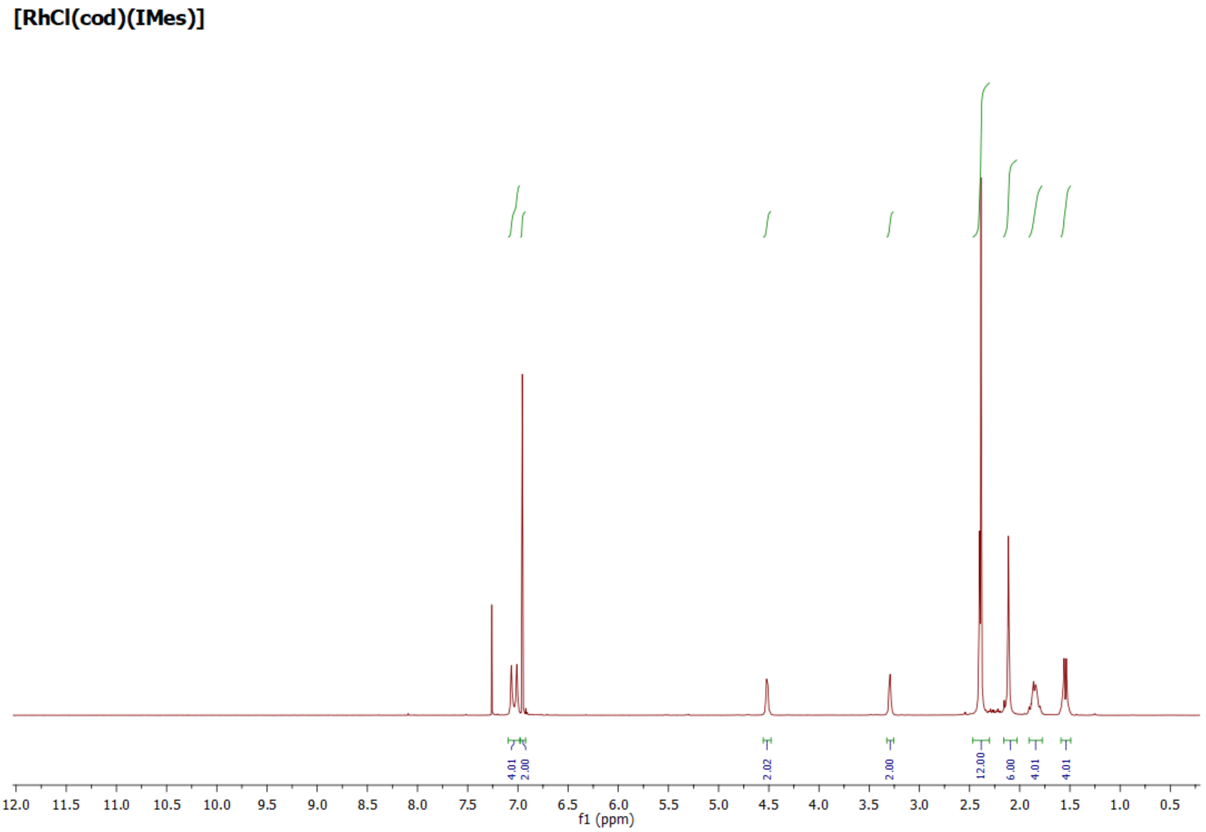

Figure S1. ^{1}H NMR (400 MHz, $CDCl_3$) of [RhCl(cod)(IMes)]

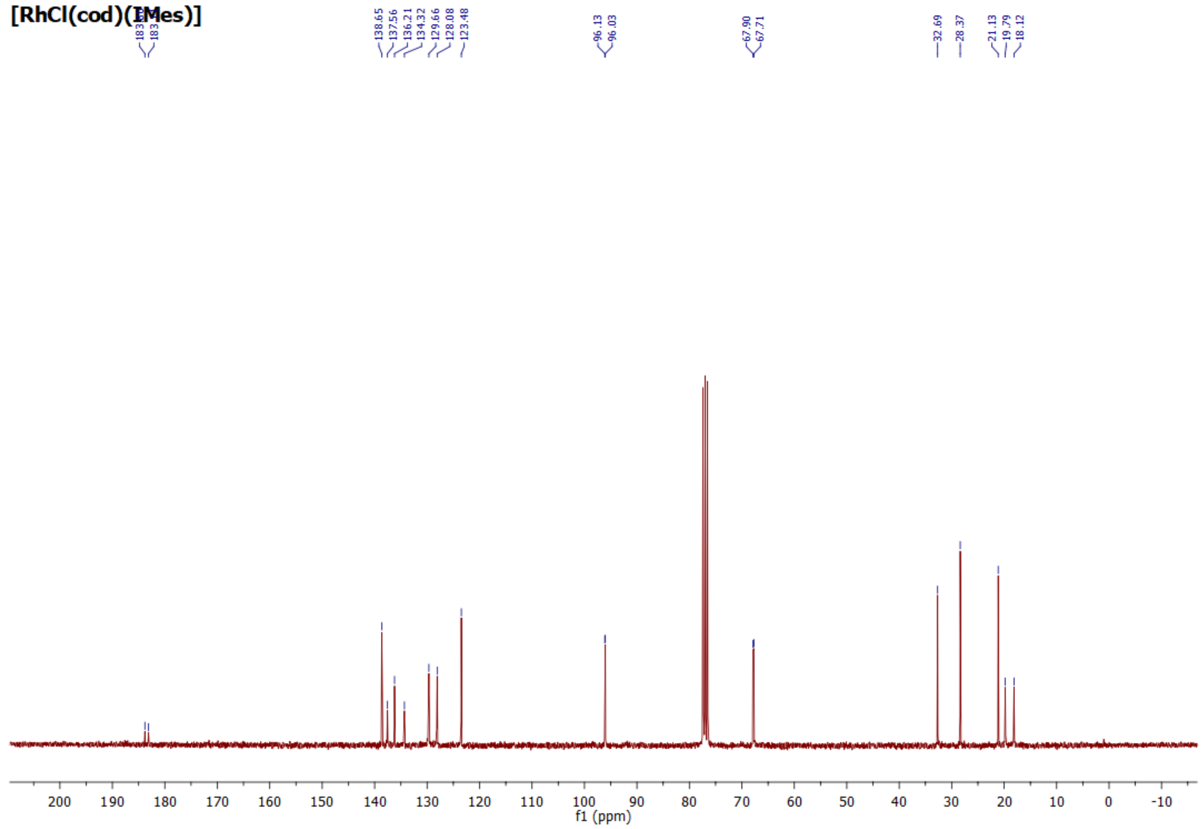

Figure S2. ^{13}C NMR (101 MHz, $CDCl_3$) of [RhCl(cod)(IMes)]

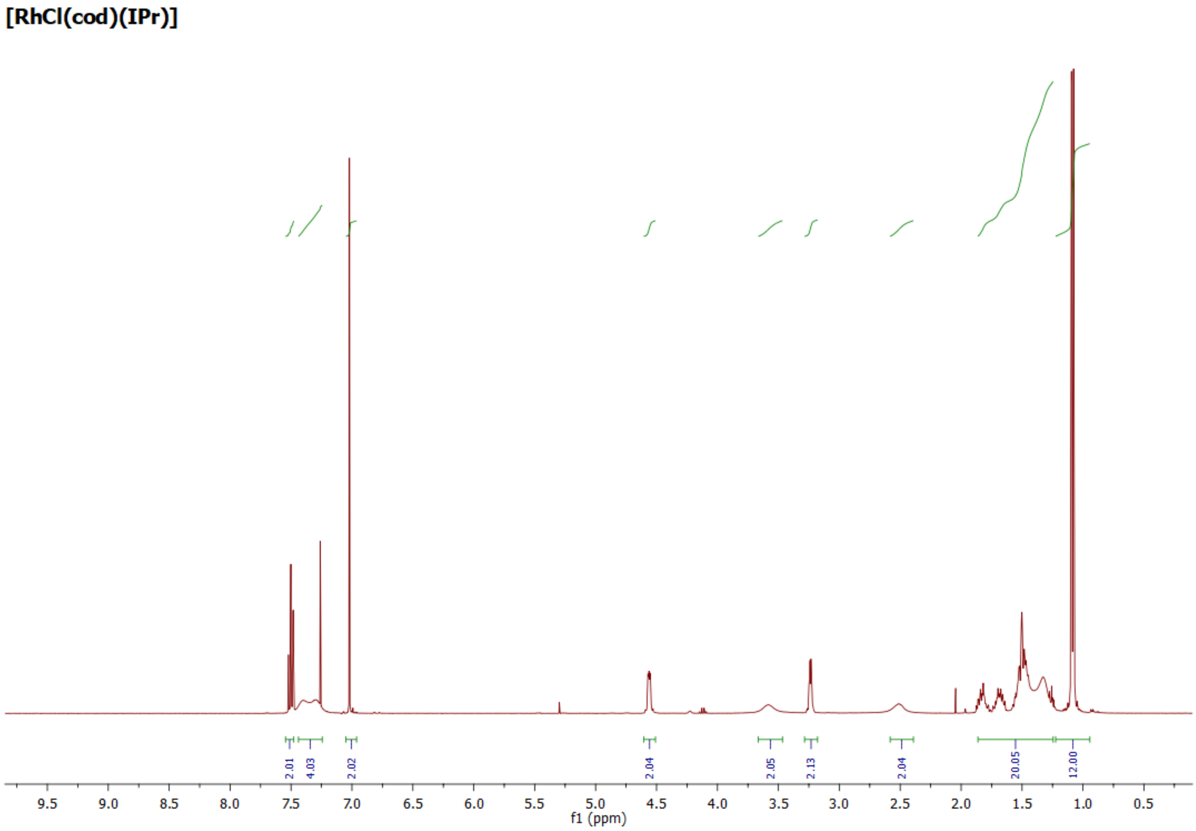

Figure S3. ^{1}H NMR (400 MHz, $CDCl_3$) of [RhCl(cod)(IPr)]

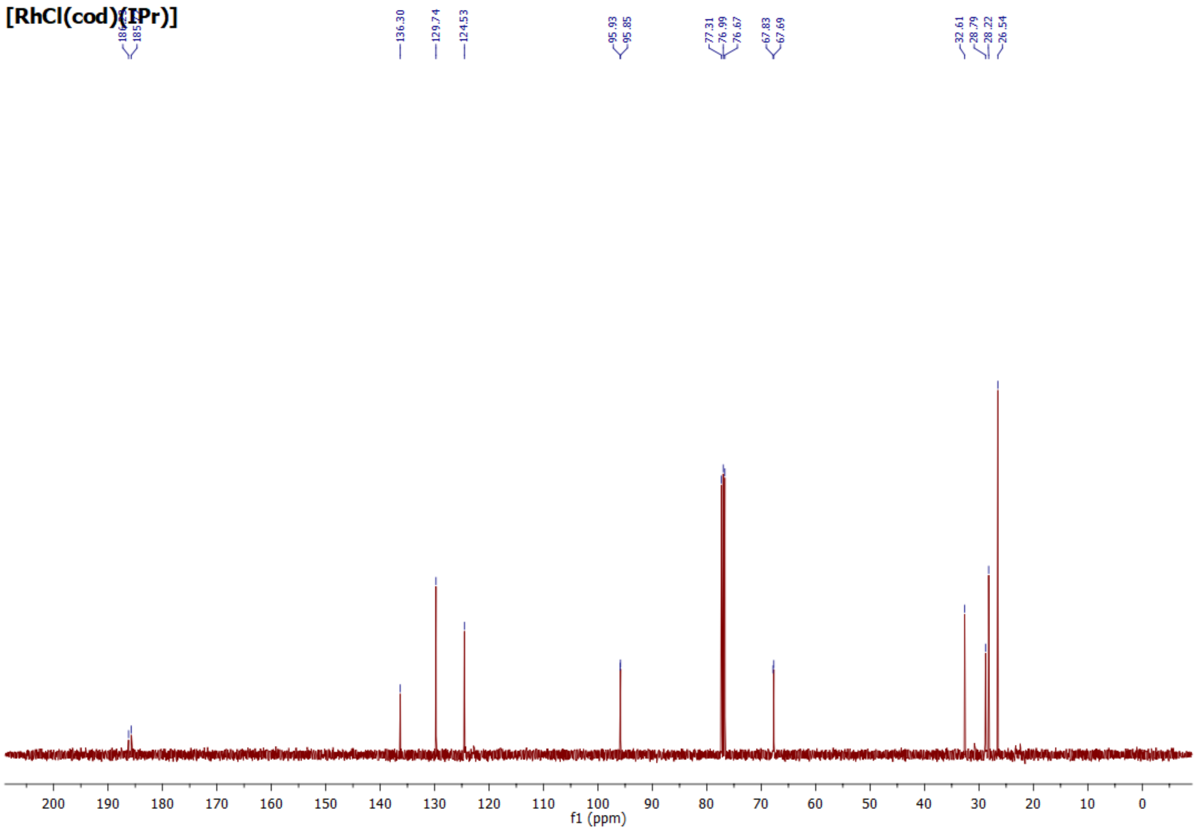

Figure S4. ^{13}C NMR (101 MHz, $CDCl_3$) of [RhCl(cod)(IPr)]

[RhCl(cod)(IPr*Et)]

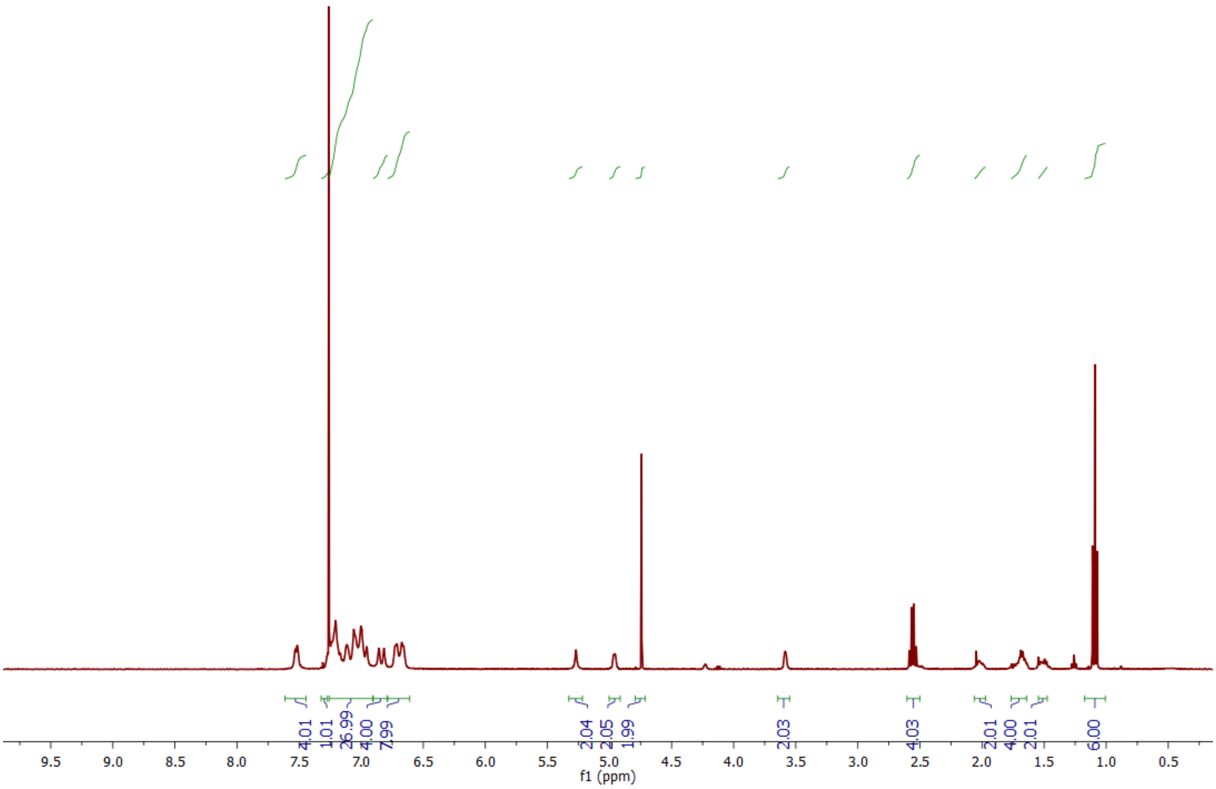

Figure S5. ^{1}H NMR (400 MHz, $CDCl_3$) of [RhCl(cod)(IPr*^{Et})]

[RhCl(cod)(IPr*Et)]

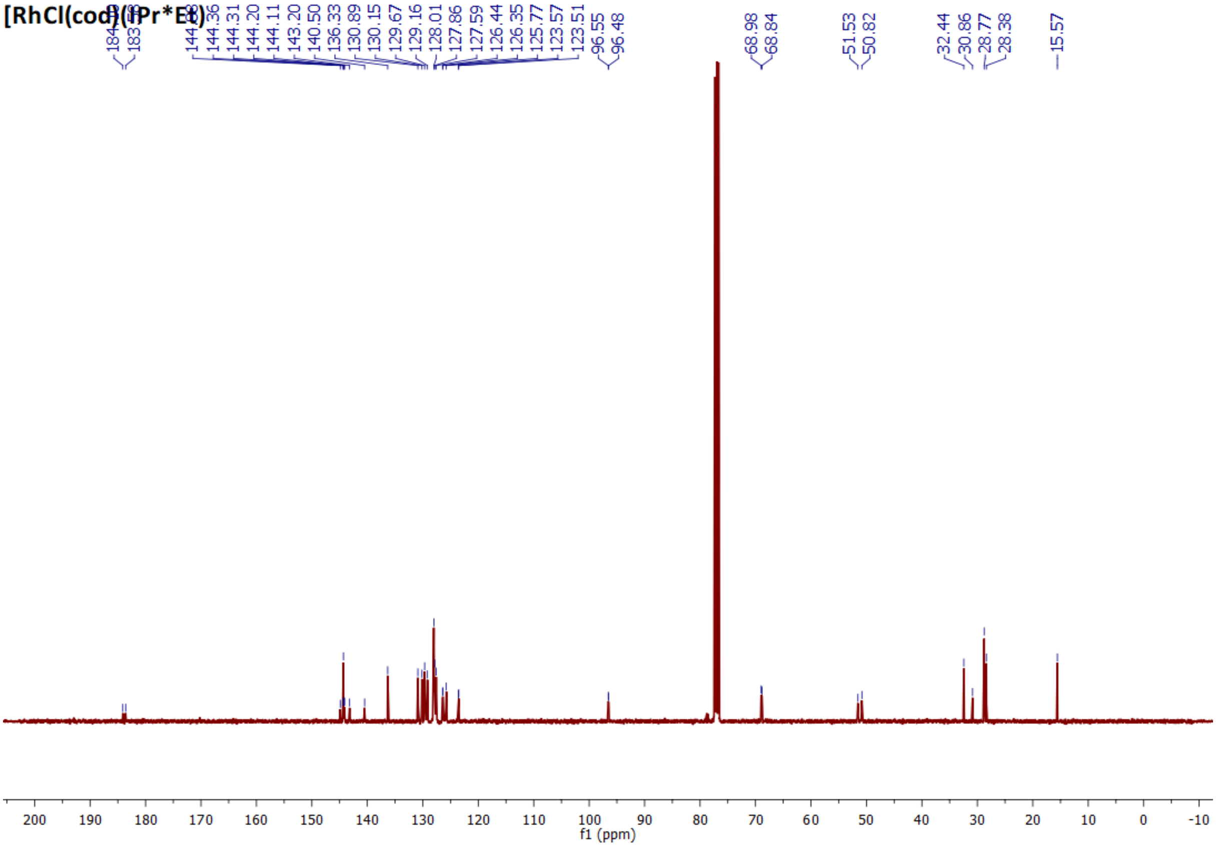

Figure S6. ^{13}C NMR (101 MHz, $CDCl_3$) of [RhCl(cod)(IPr*^{Et})]

[RhCl(cod)(IPr*Ph2)]

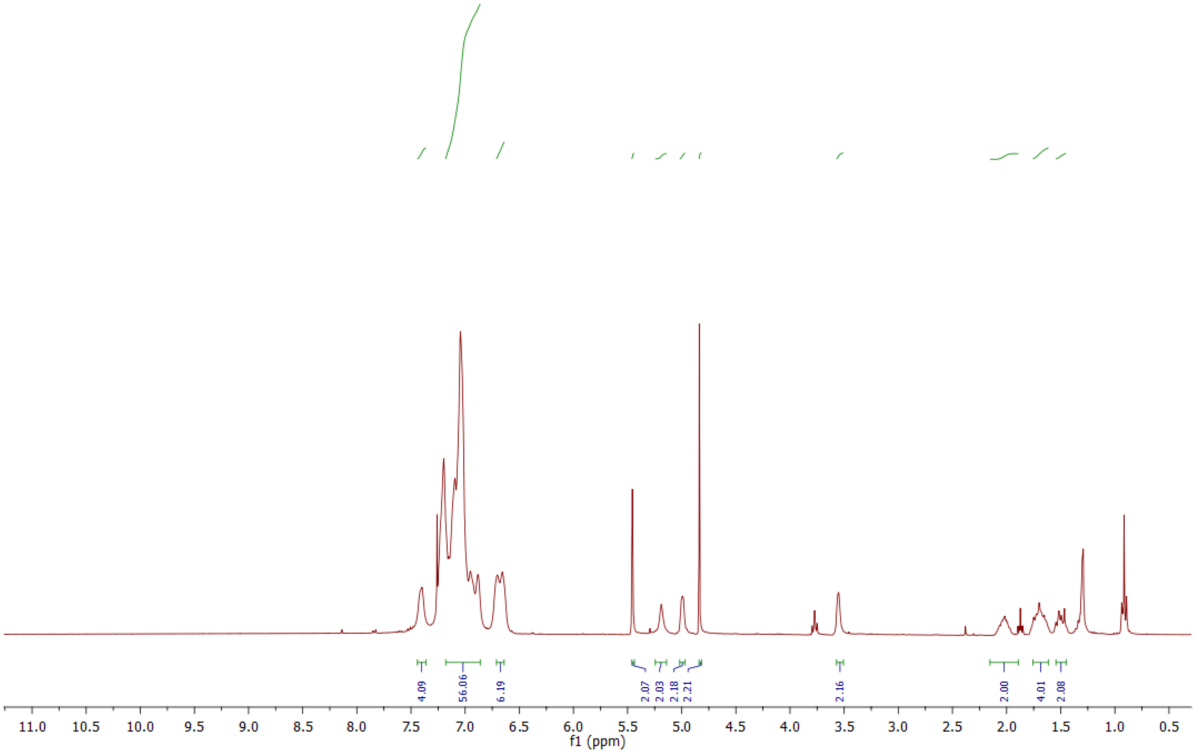

Figure S7. ^{1}H NMR (400 MHz, $CDCl_3$) of [RhCl(cod)(IPr*^{Ph2})]

[RhCl(cod)(IPr*Ph2)]

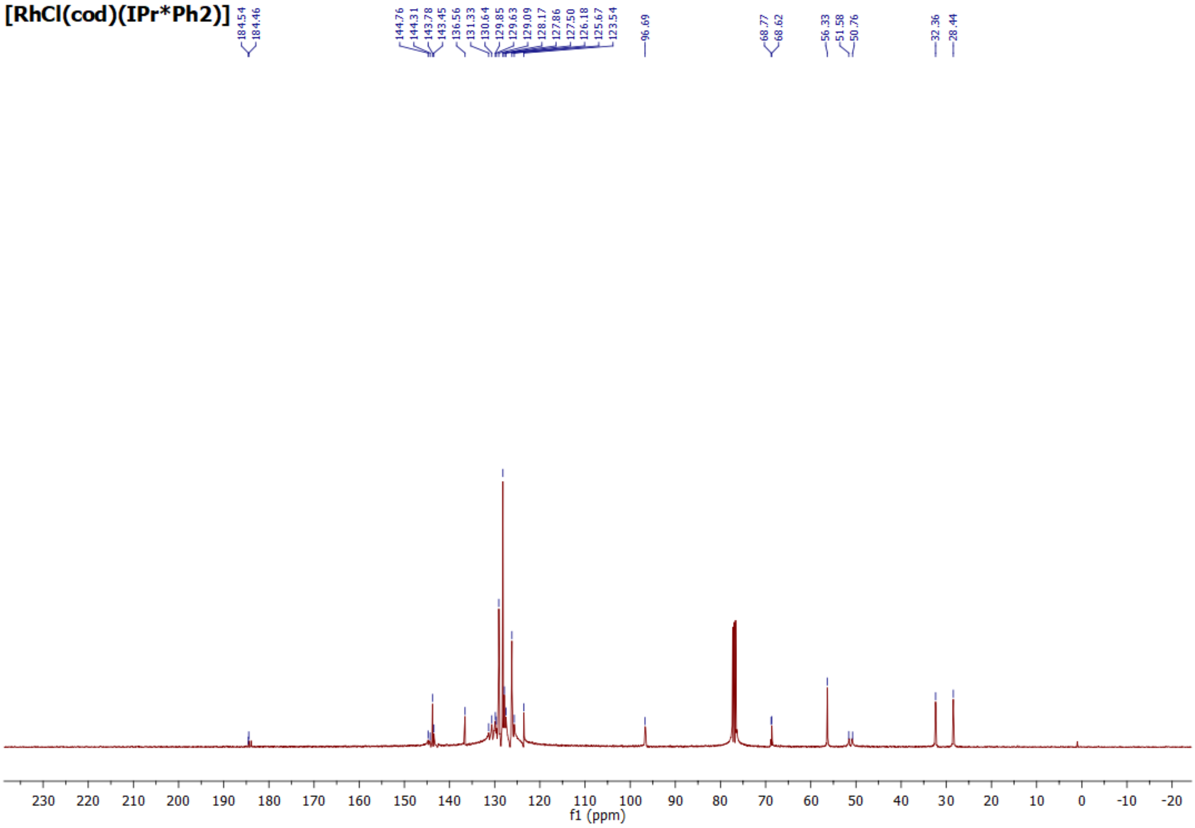

Figure S8. ^{13}C NMR (101 MHz, $CDCl_3$) of of [RhCl(cod)(IPr*^{Ph2})]

12. NMR spectra of isolated products

12.1. NMR spectra of silylthioethers

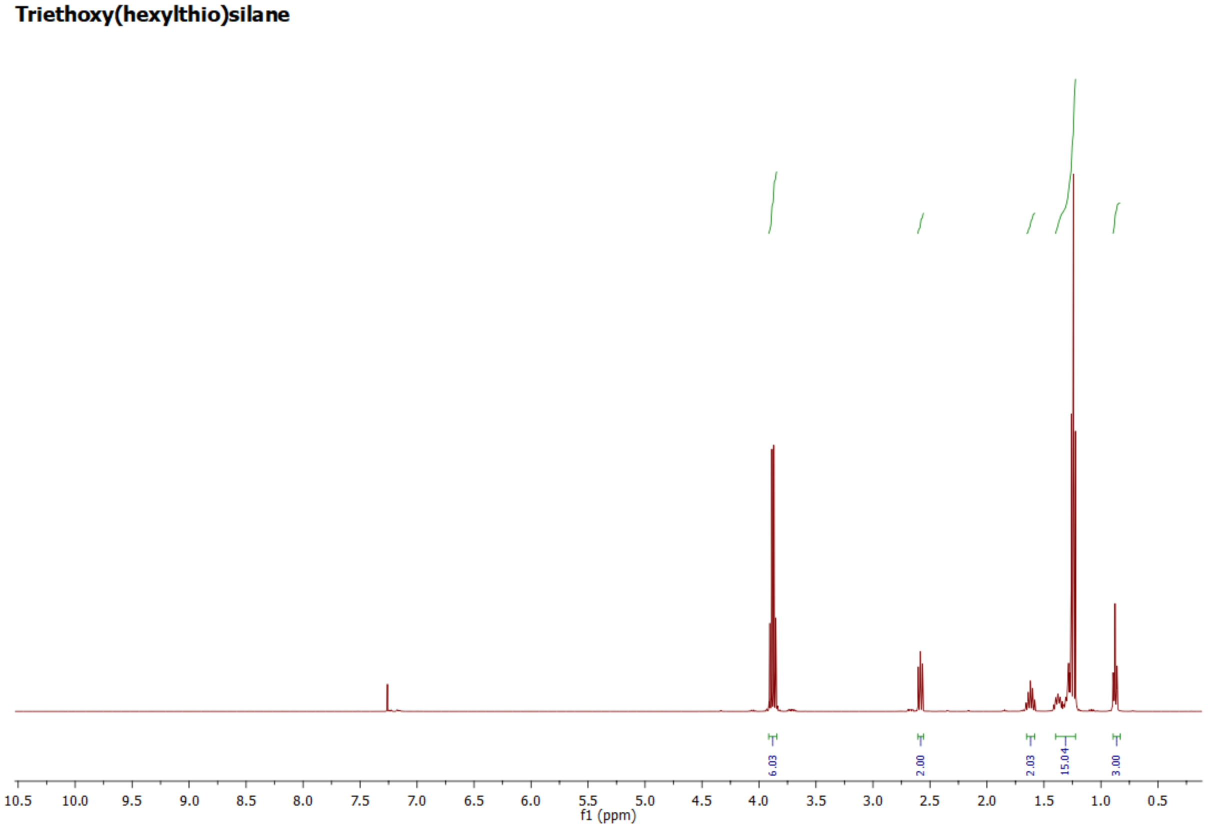

Figure S9. ^{1}H NMR (400 MHz, $CDCl_3$) of triethoxy(hexylthio)silane (**3 a-a**)

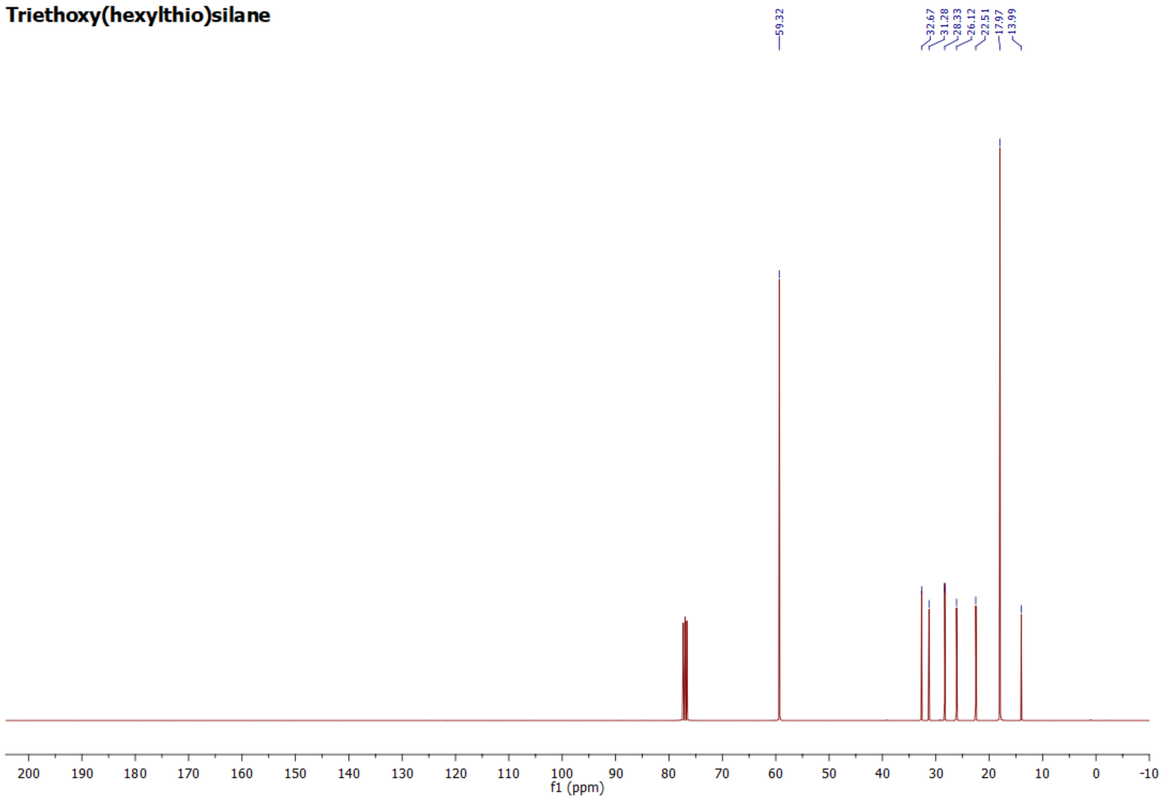

Figure S10. ^{13}C NMR (100 MHz, $CDCl_3$) of triethoxy(hexylthio)silane (**3 a-a**)

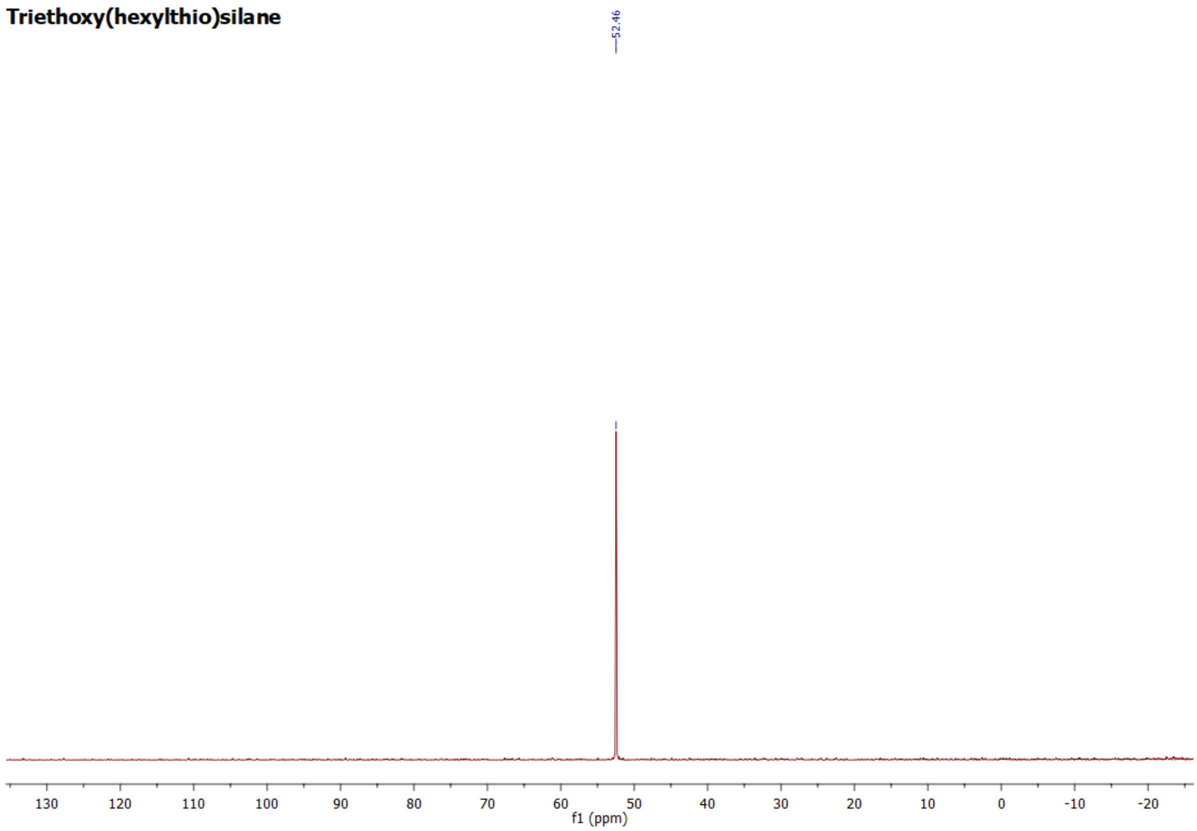

Figure S11. ^{29}Si NMR (79 MHz, $CDCl_3$) of triethoxy(hexylthio)silane (**3 a-a**)

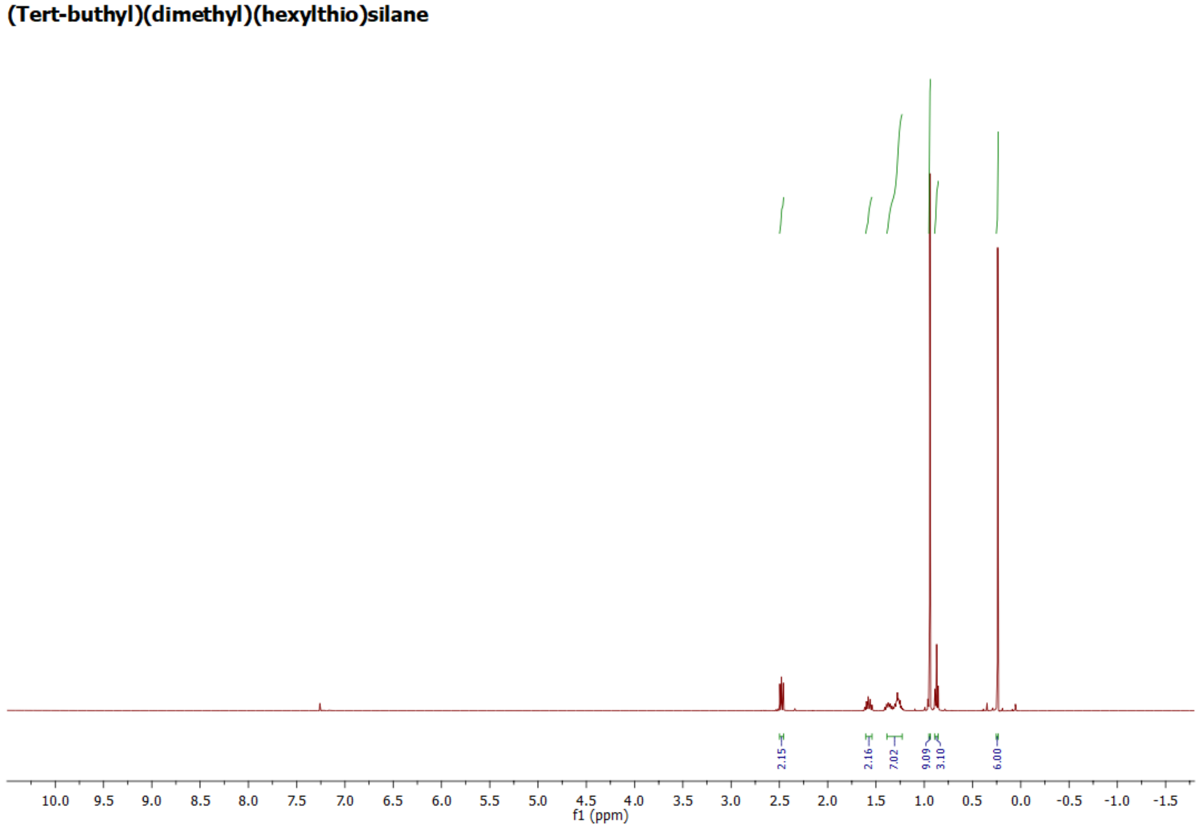

Figure S12. ^{1}H NMR (400 MHz, $CDCl_3$) of (*tert*-buthyl)(dimethyl)(hexylthio)silane (**3 b-a**)

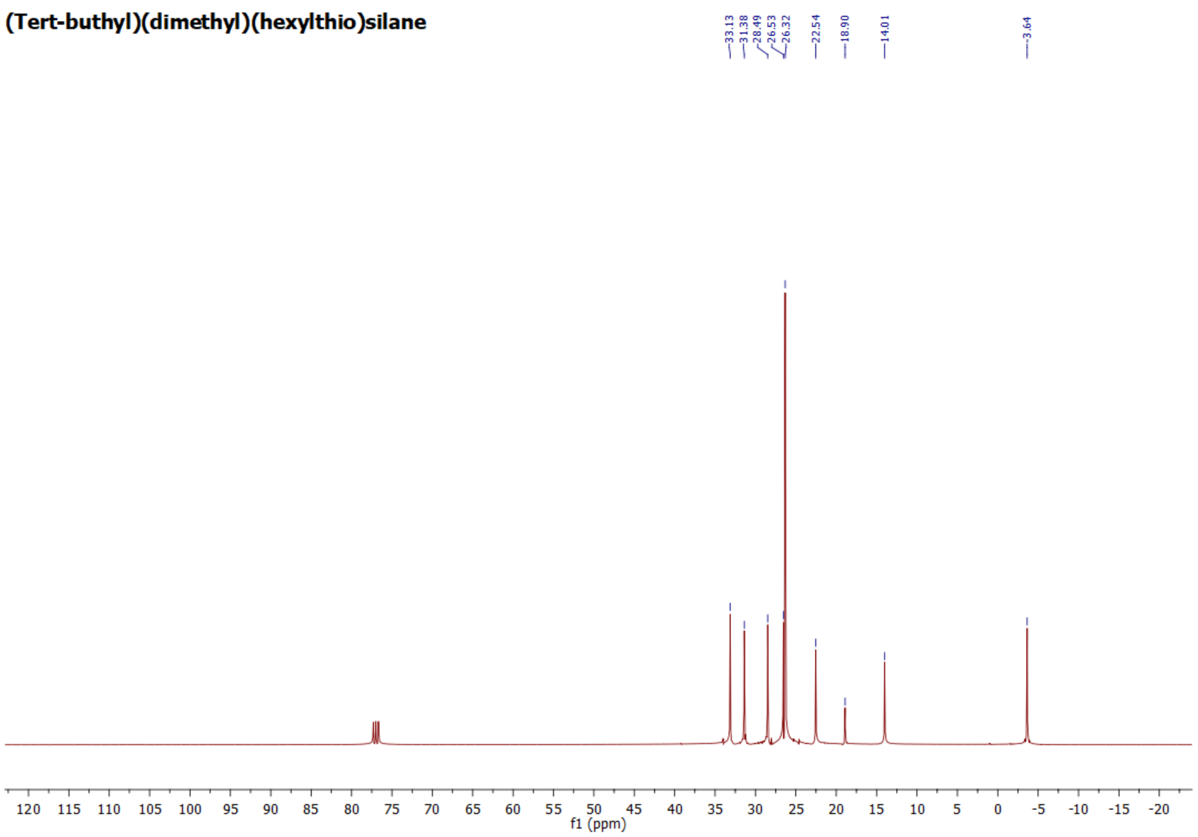

Figure S13. ^{13}C NMR (100 MHz, $CDCl_3$) of (*tert*-buthyl)(dimethyl)(hexylthio)silane (**3 b-a**)

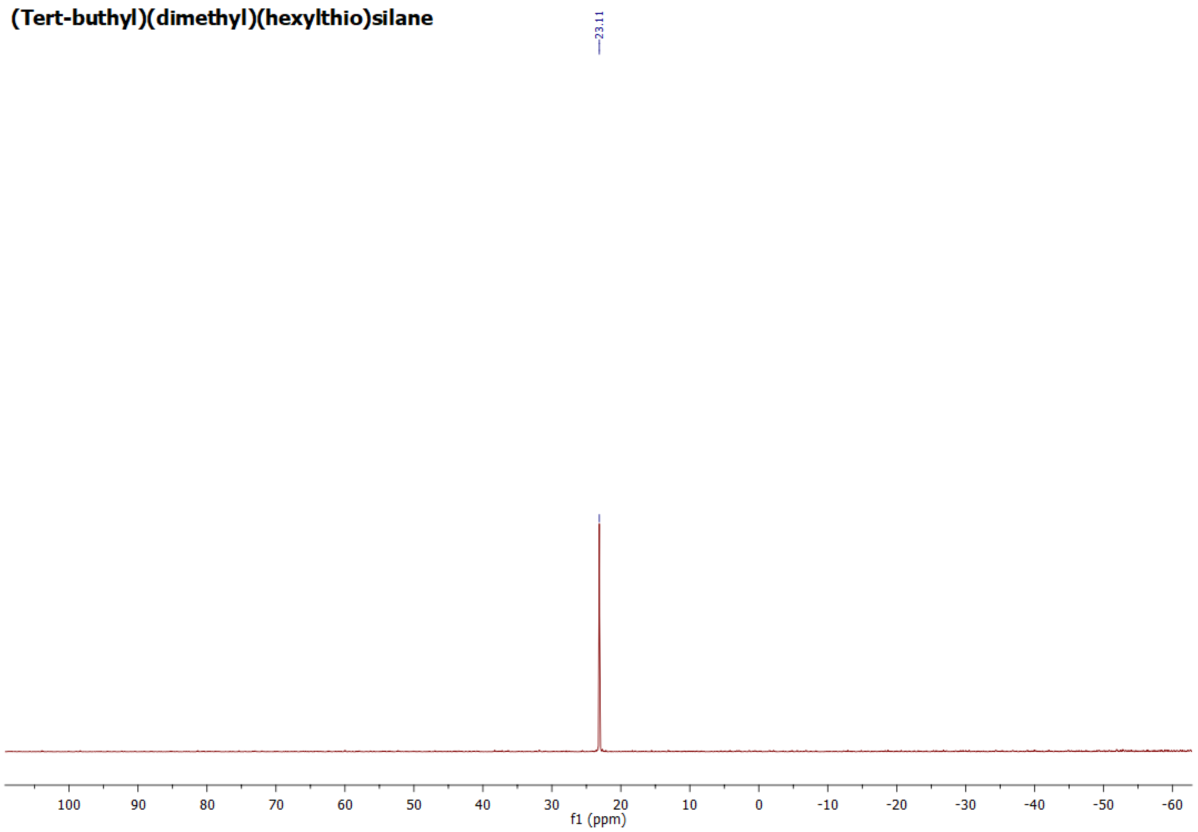

Figure S14. ^{29}Si NMR (79 MHz, $CDCl_3$) of (*tert*-buthyl)(dimethyl)(hexylthio)silane (**3 b-a**)

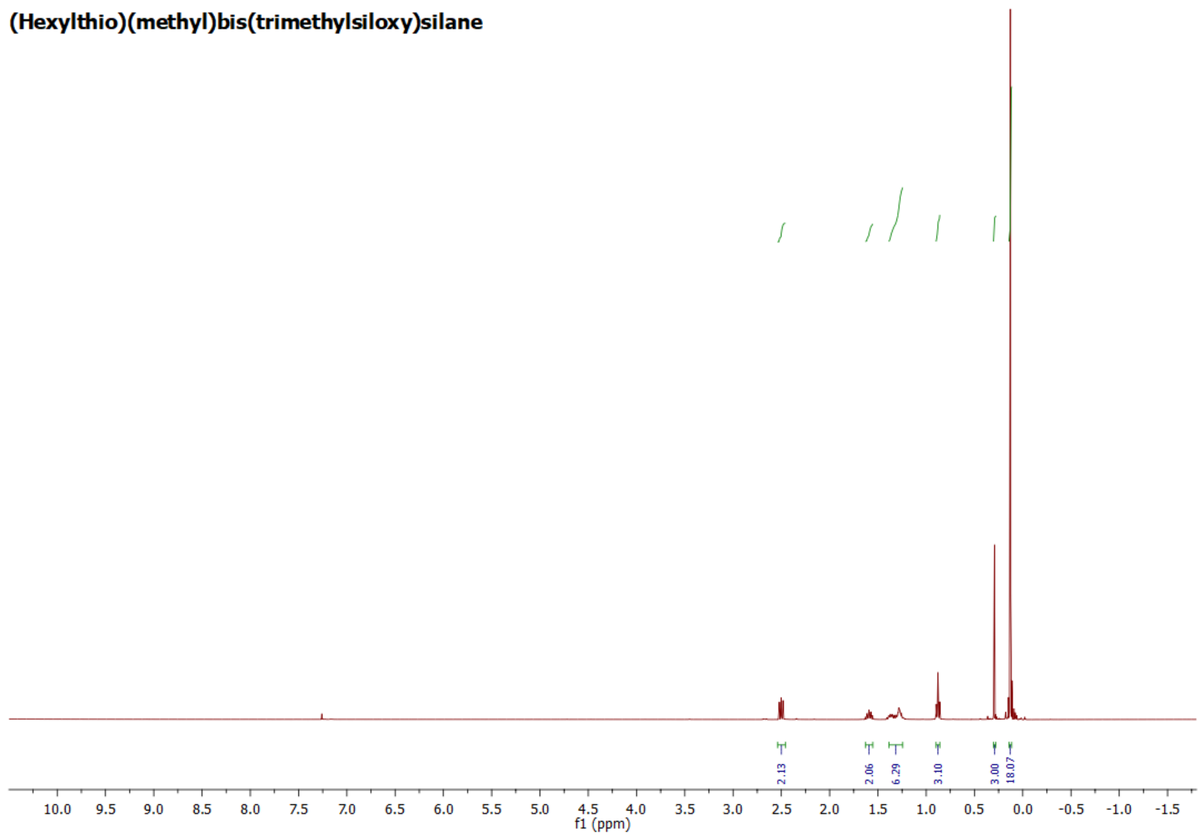

Figure S15. ^{1}H NMR (400 MHz, $CDCl_3$) of (hexylthio)(methyl)bis(trimethylsiloxy)silane (**3 e-a**)

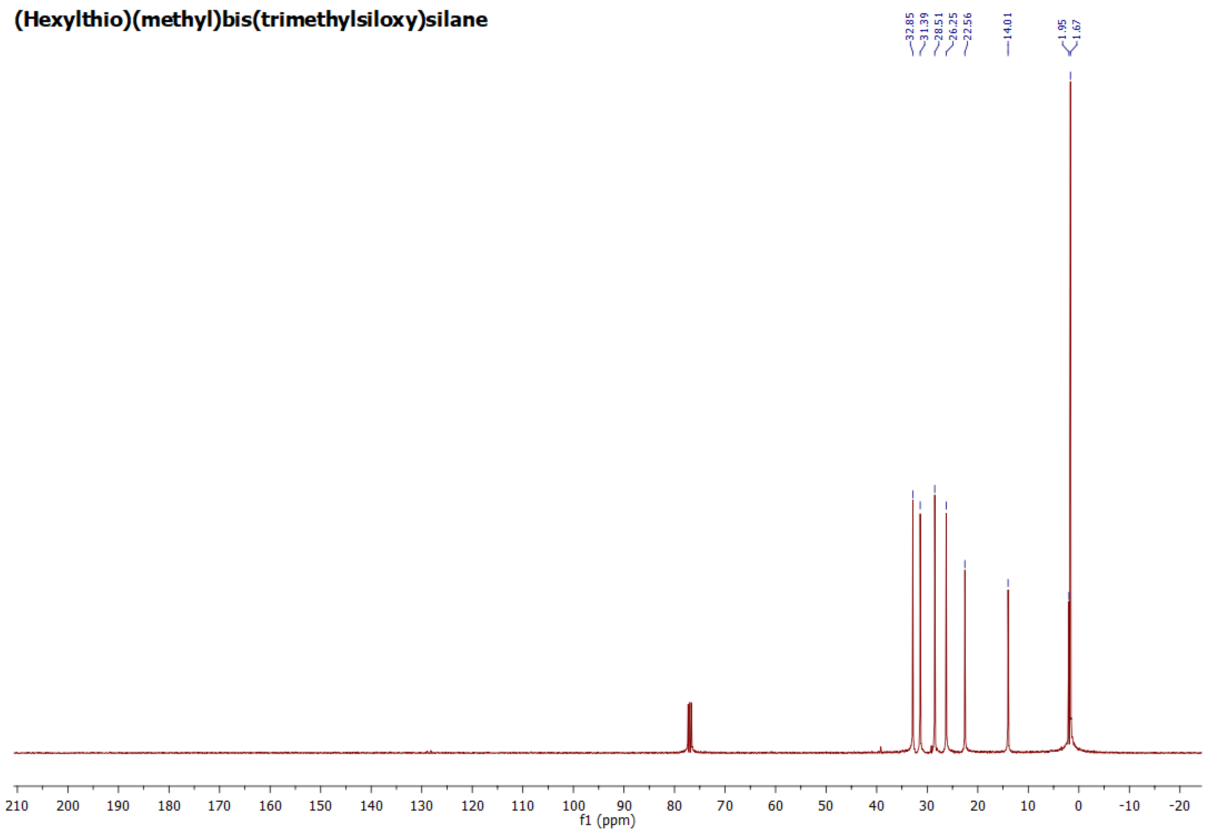

Figure S16. ^{13}C NMR (100 MHz, $CDCl_3$) of (hexylthio)(methyl)bis(trimethylsiloxy)silane (**3 e-a**)

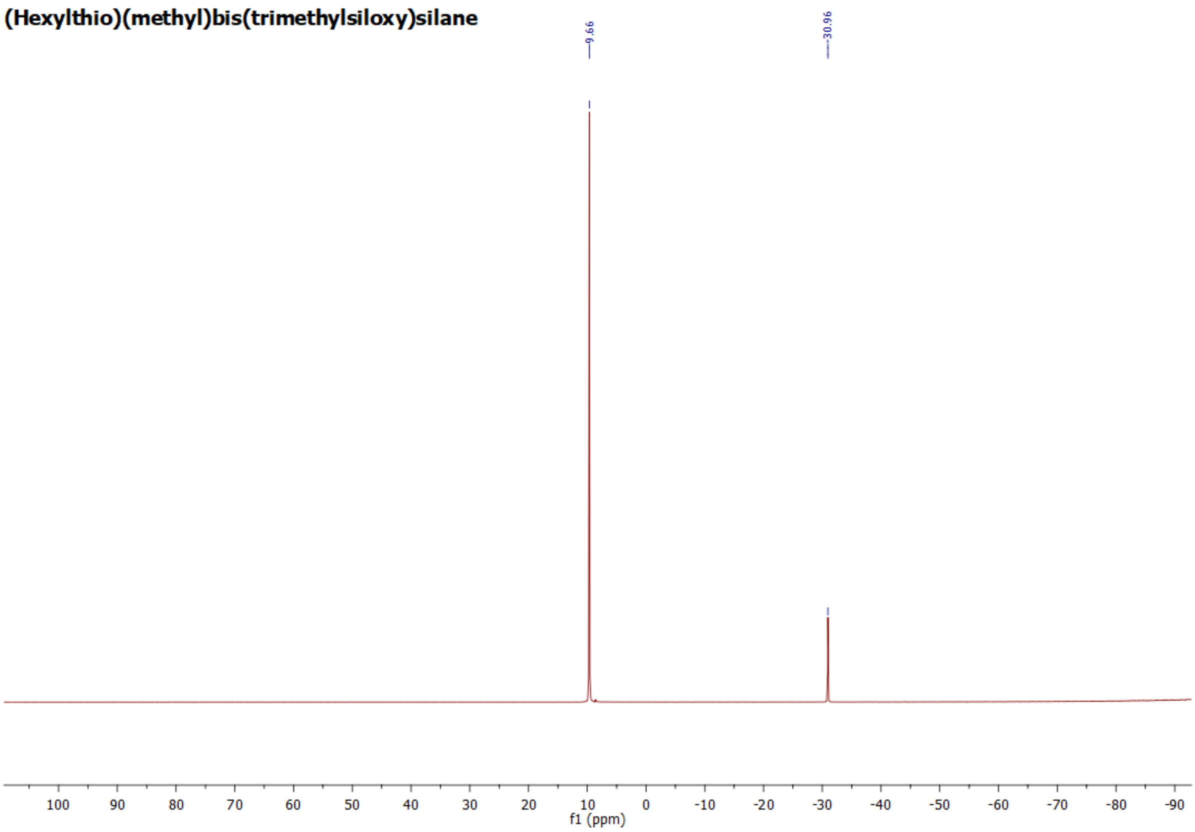

Figure S17. ^{29}Si NMR (79 MHz, $CDCl_3$) of (hexylthio)(methyl)bis(trimethylsiloxy)silane (**3 e-a**)

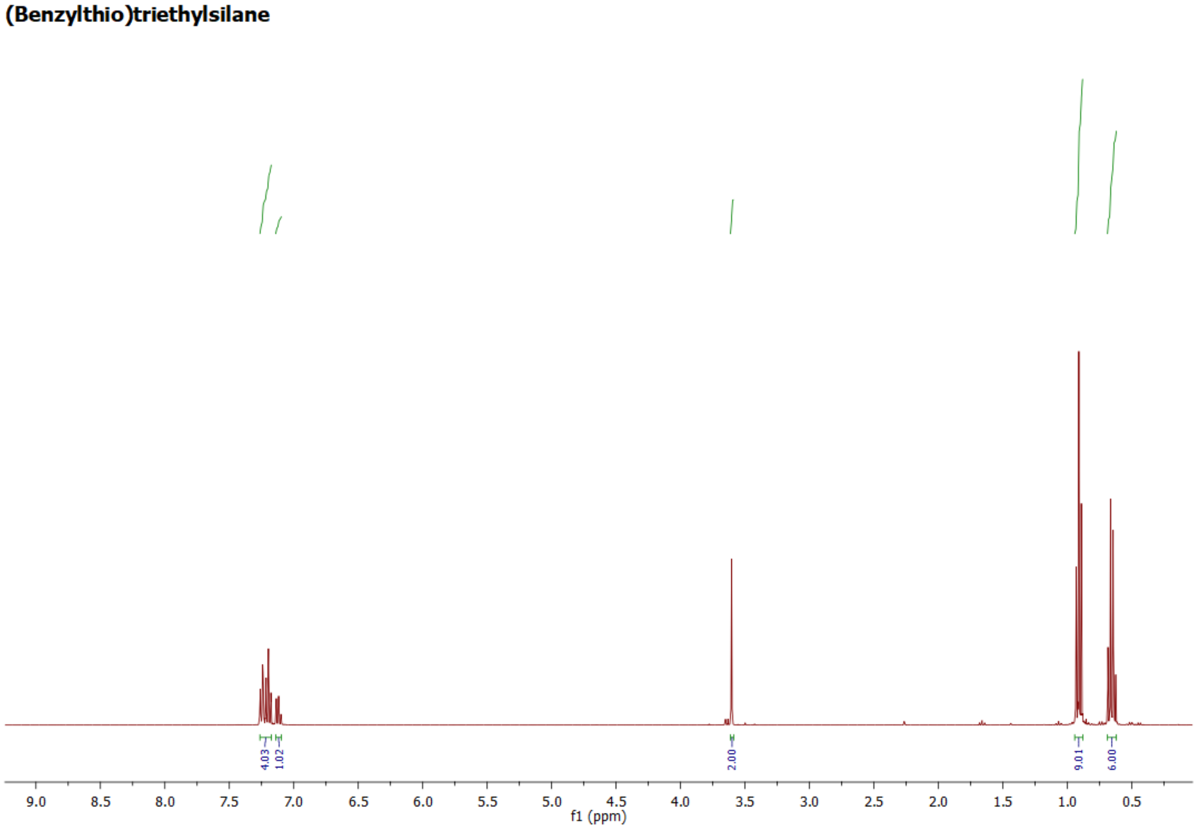

Figure S18. ^{1}H NMR (400 MHz, $CDCl_3$) of (benzylthio)triethylsilane (**3 c-d**)

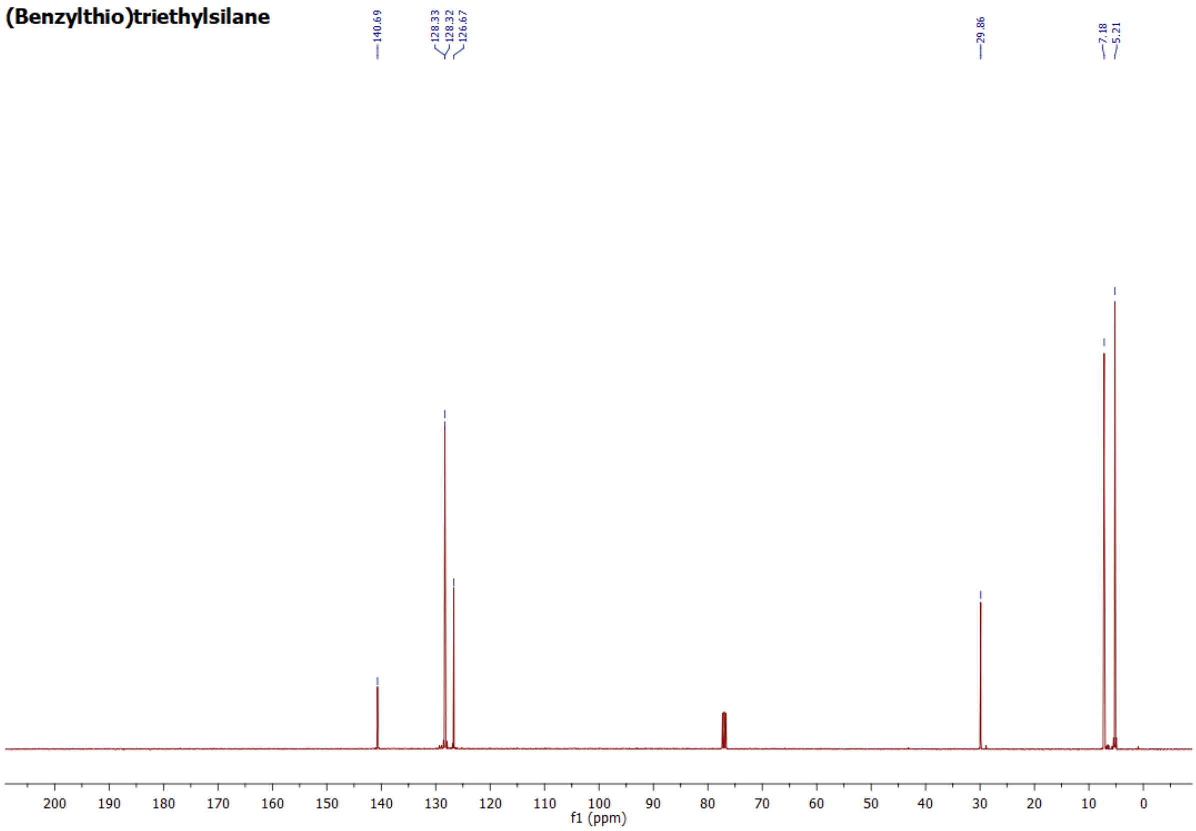

Figure S19. ^{13}C NMR (100 MHz, $CDCl_3$) of (benzylthio)triethylsilane (**3 c-d**)

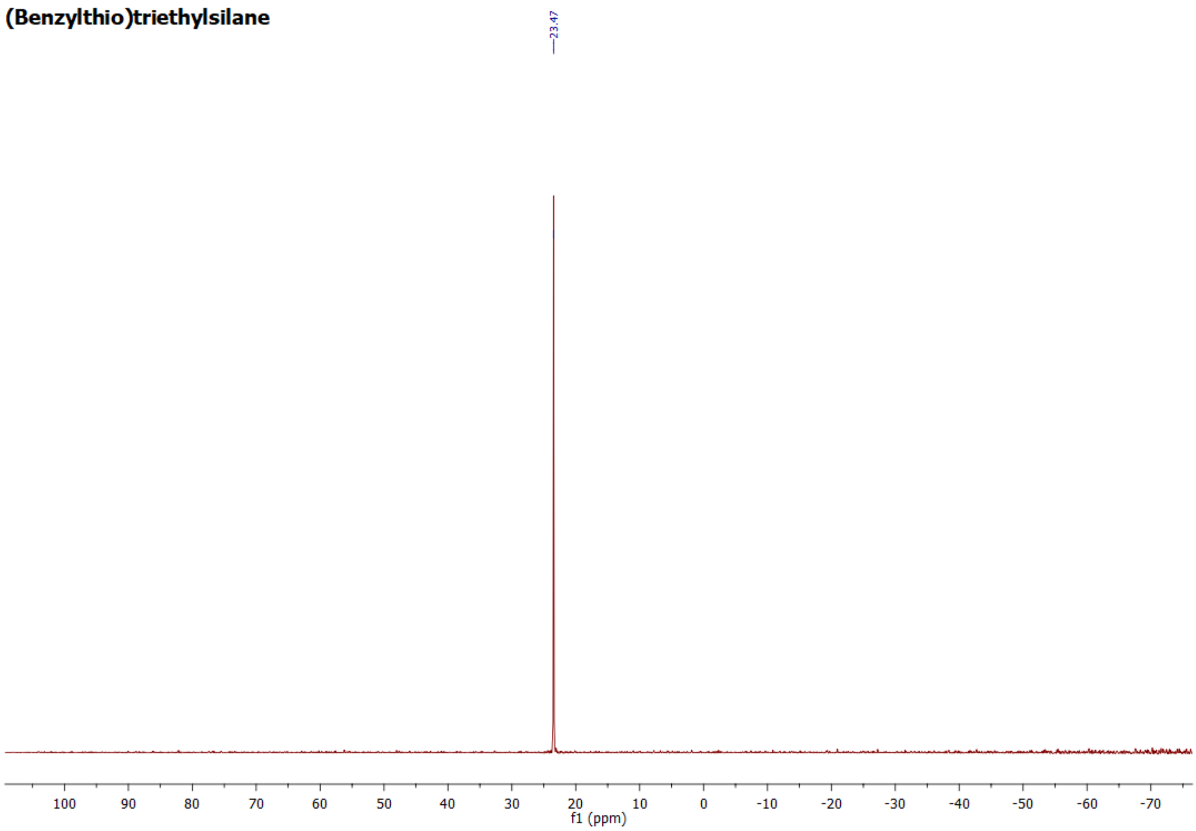

Figure S20. ^{29}Si NMR (79 MHz, $CDCl_3$) of (benzylthio)triethylsilane (**3 c-d**)

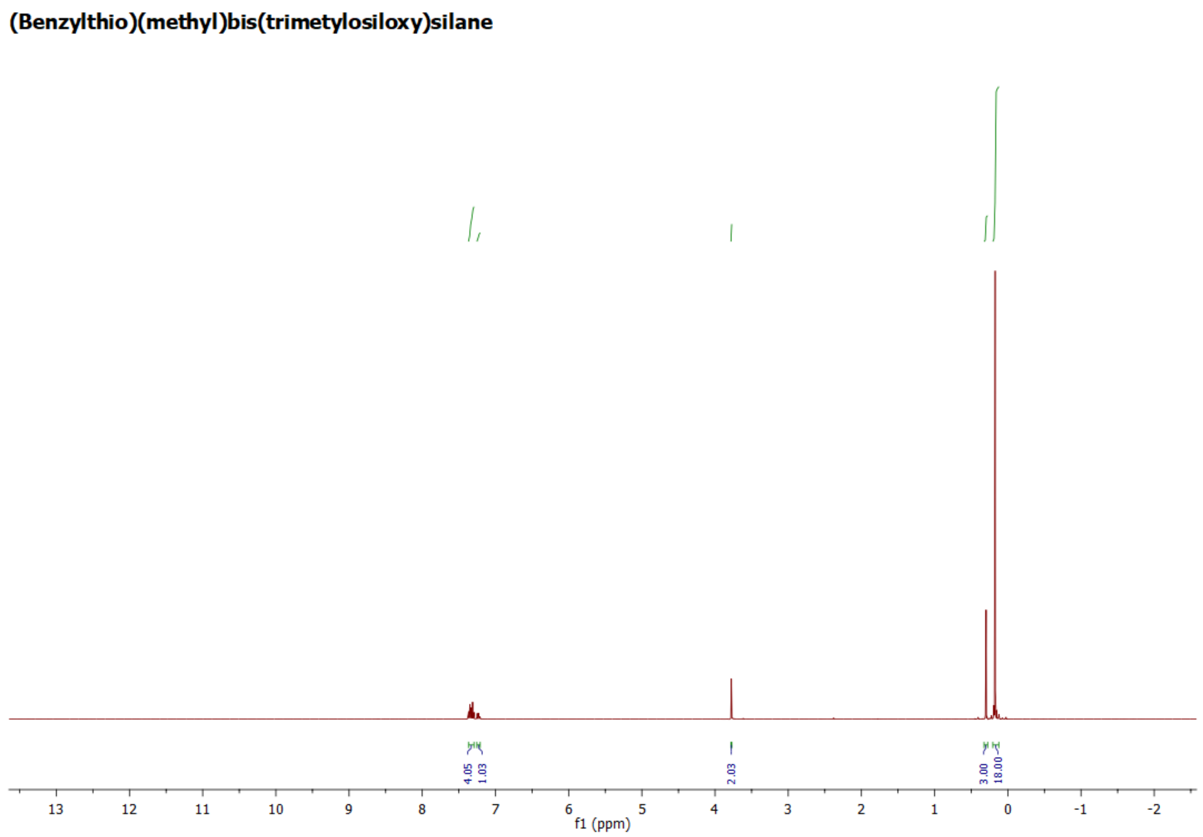

Figure S21. ^{1}H NMR (400 MHz, $CDCl_3$) of (benzylthio)(methyl)bis(trimetylosiloxy)silane (**3 e-d**)

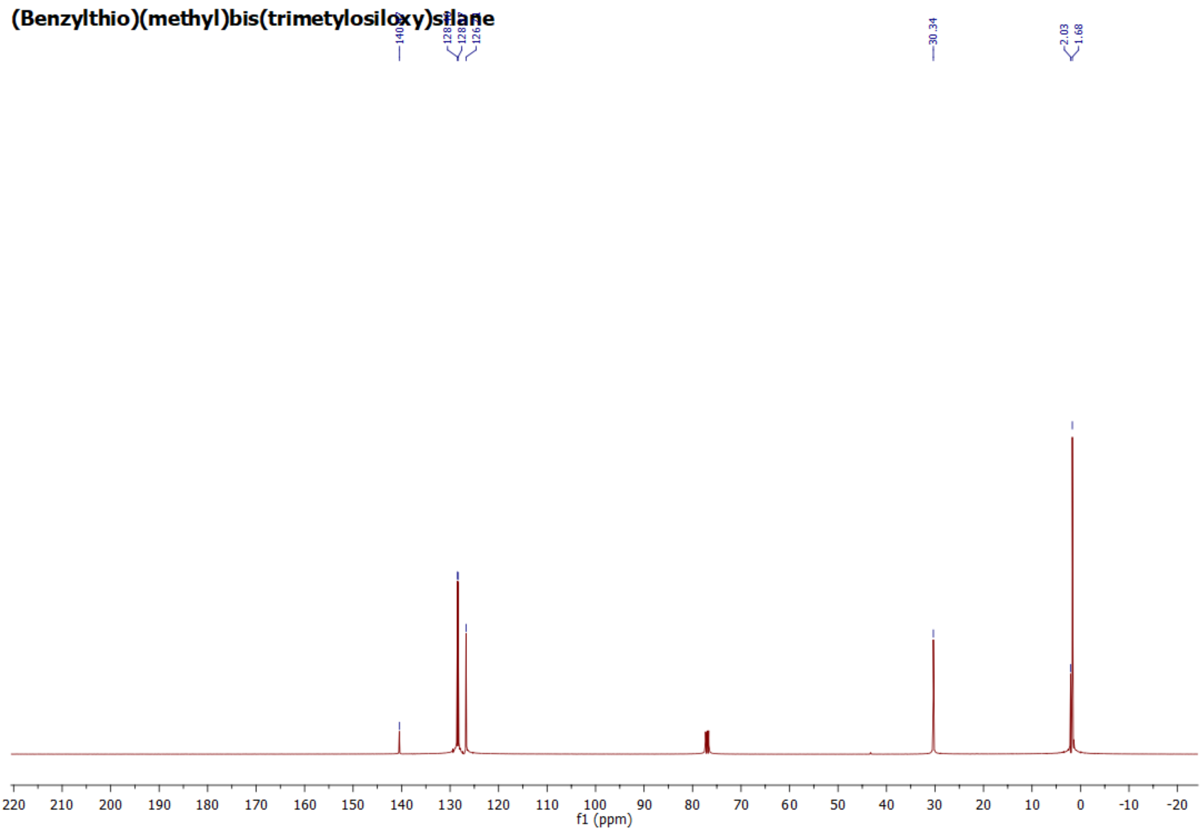

Figure S22. ^{13}C NMR (75 MHz, $CDCl_3$) of (benzylthio)(methyl)bis(trimetylosiloxy)silane (**3 e-d**)

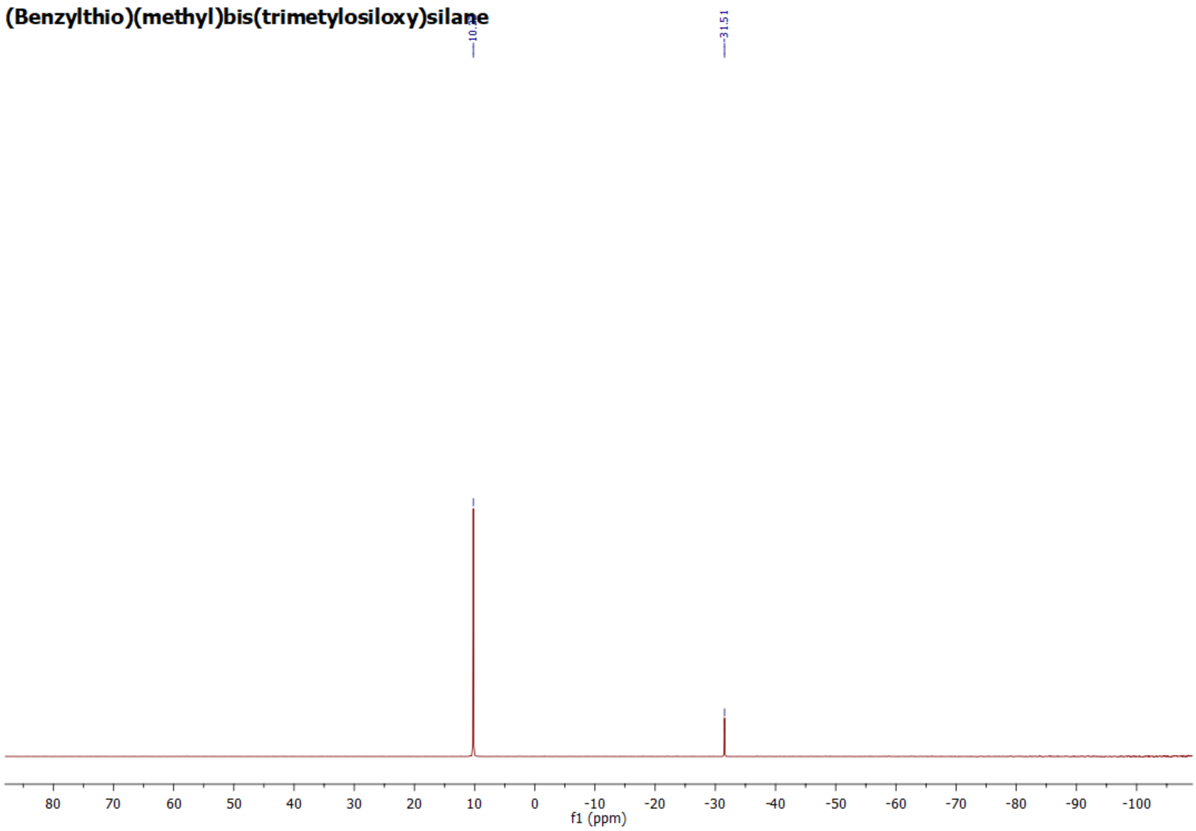

Figure S23. ^{29}Si NMR (79 MHz, $CDCl_3$) of (benzylthio)(methyl)bis(trimetylosiloxy)silane (**3 e-d**)

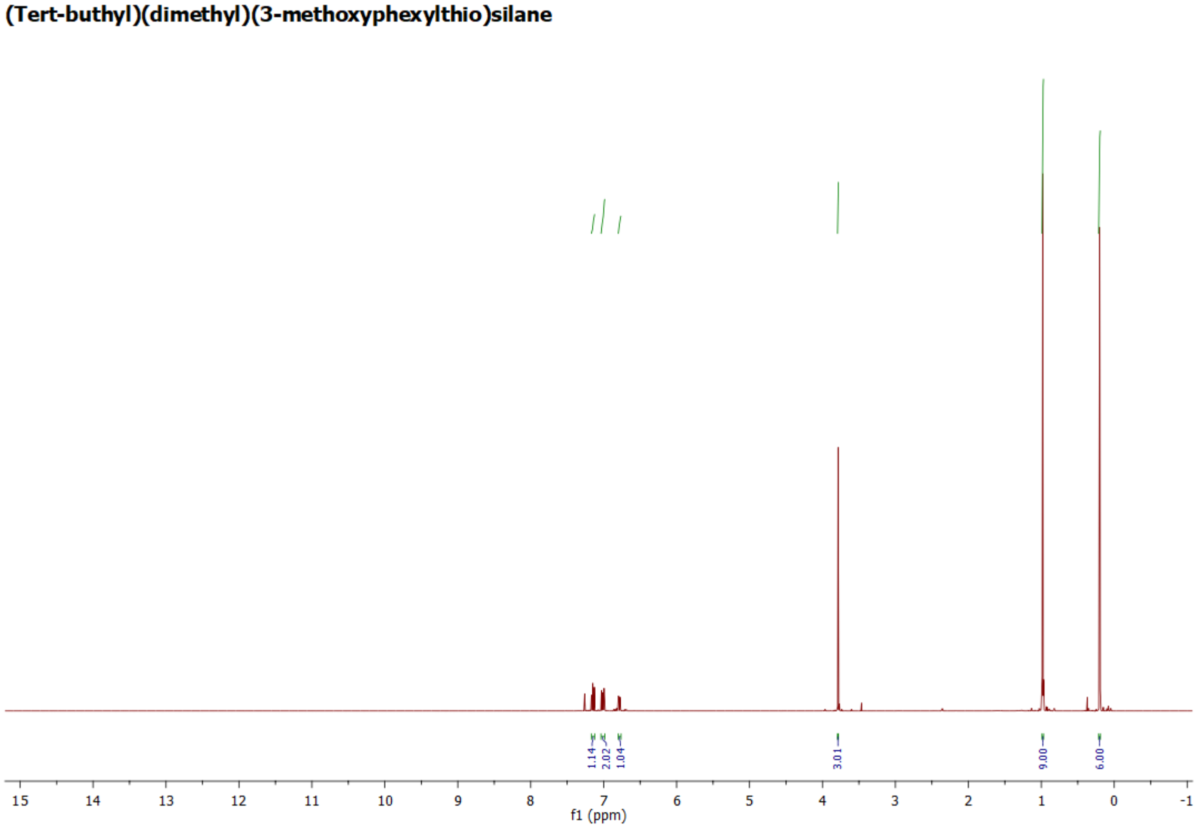

Figure S24. ^{1}H NMR (400 MHz, $CDCl_3$) of (*tert*-buthyl)(dimethyl)(3-methoxyphexylthio)silane (**3 b-e**)

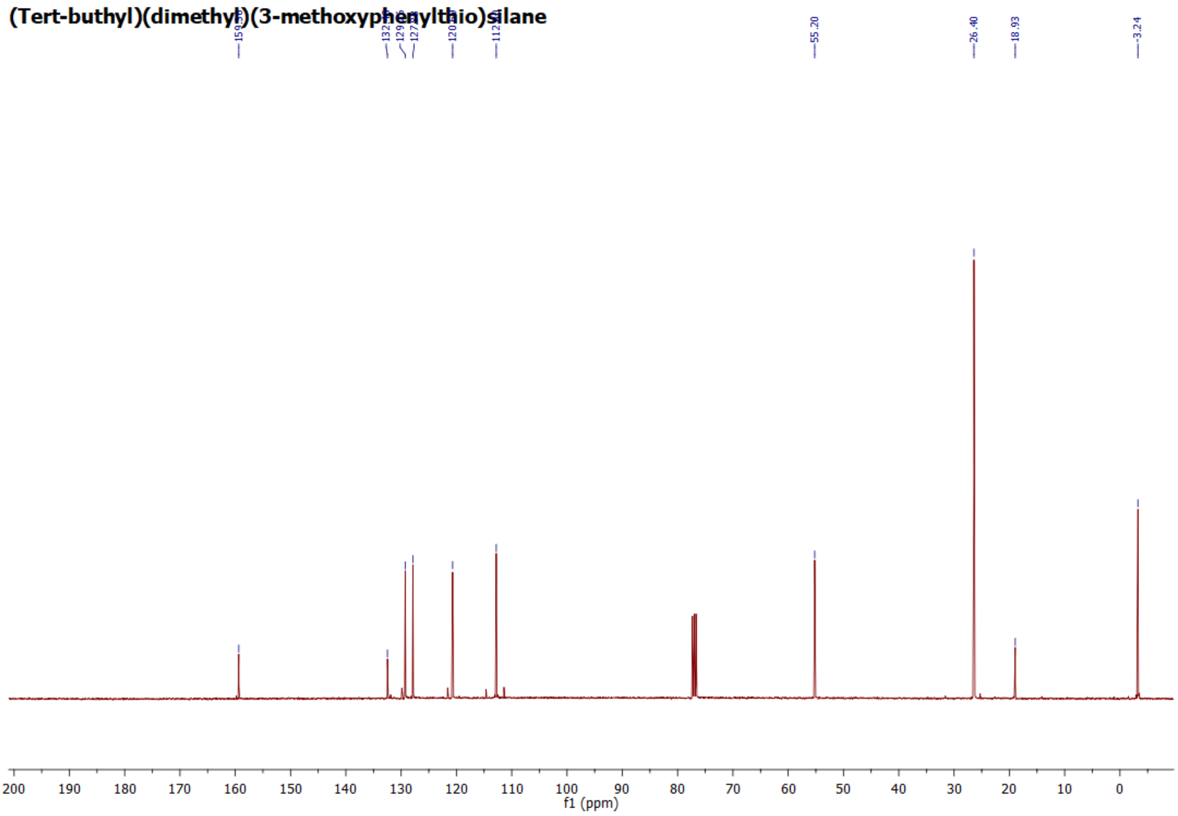

Figure S25. ^{13}C NMR (100 MHz, $CDCl_3$) of (*tert*-buthyl)(dimethyl)(3-methoxyphexylthio)silane (**3 b-e**)

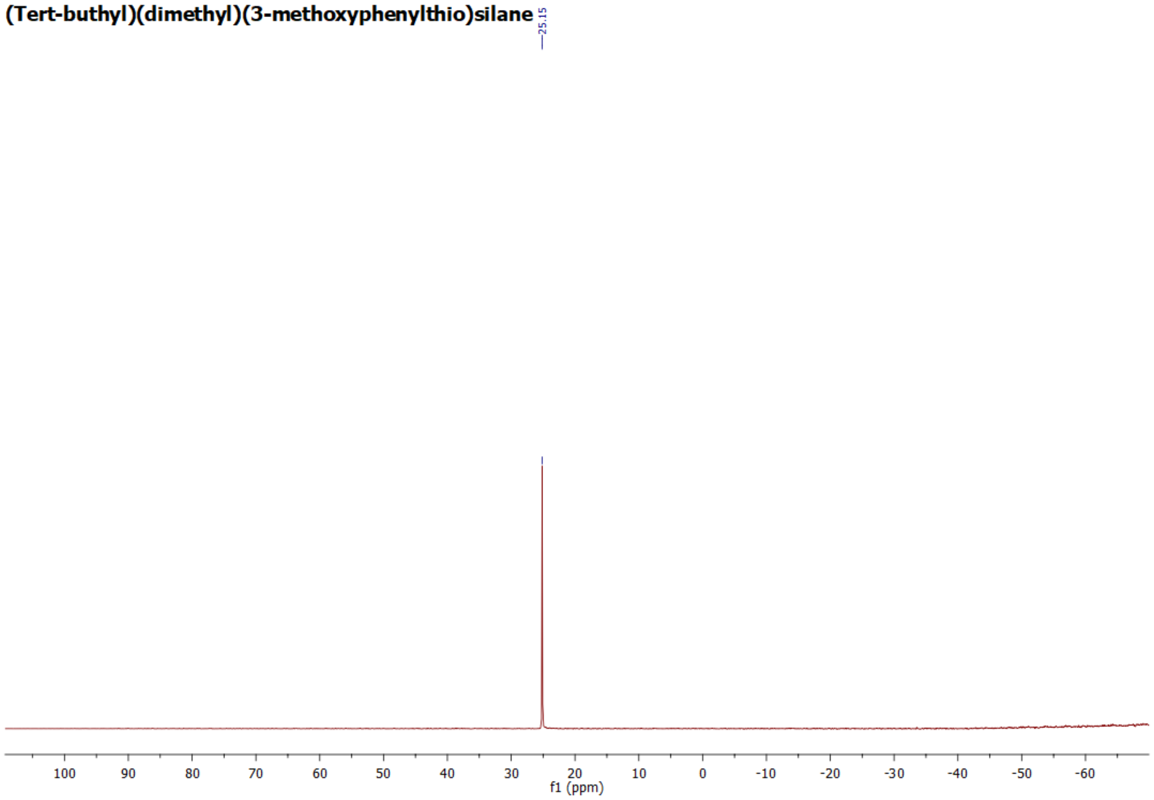

Figure S26. ^{29}Si NMR (79 MHz, $CDCl_3$) of (*tert*-buthyl)(dimethyl)(3-methoxyphexylthio)silane (**3 b-e**)

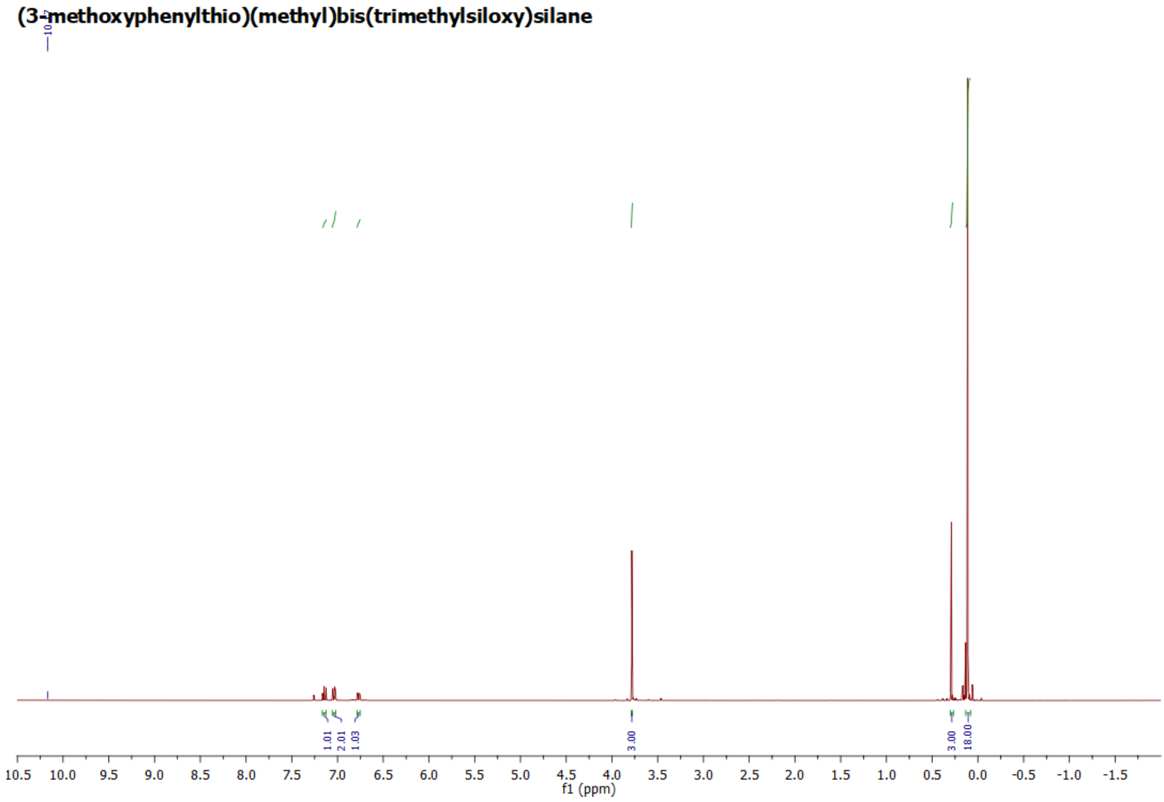

Figure S27. ^{1}H NMR (400 MHz, $CDCl_3$) of (3-methoxyphenylthio)(methyl)bis(trimethylsiloxy)silane (**3 e-e**)

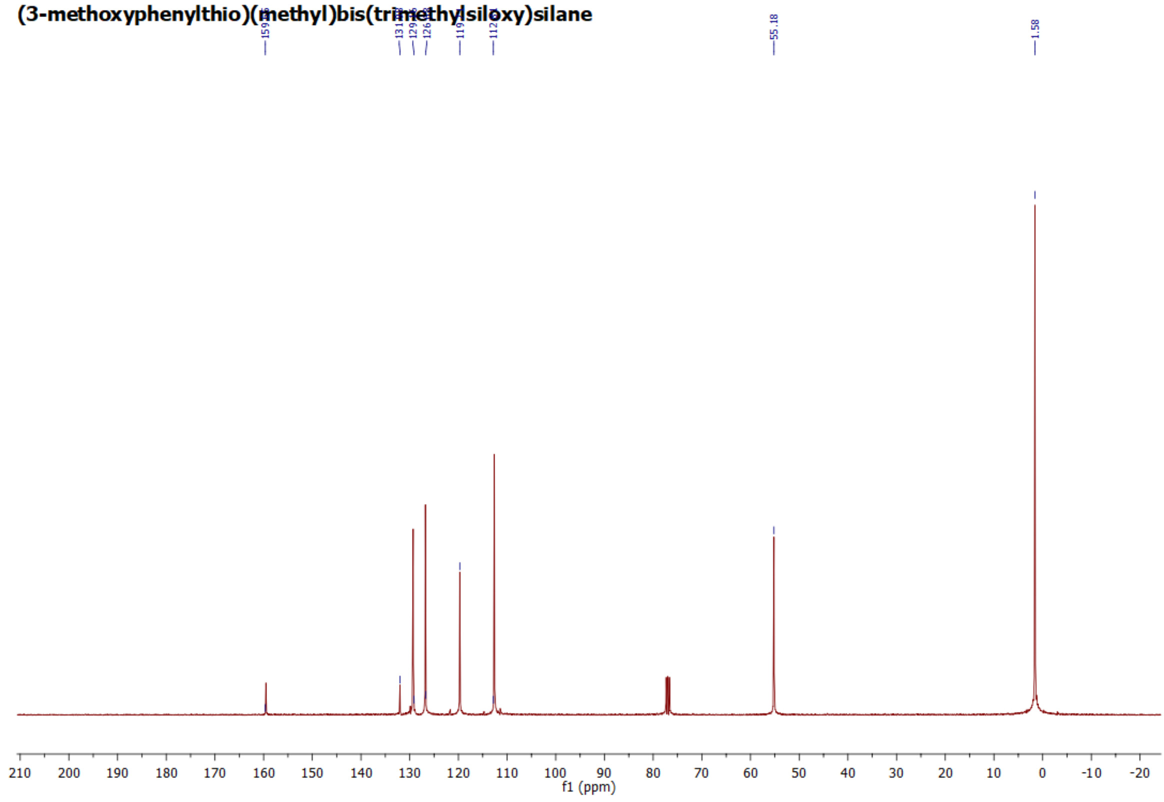

Figure S28. ^{13}C NMR (100 MHz, $CDCl_3$) of (3-methoxyphenylthio)(methyl)bis(trimethylsiloxy)silane (**3 e-e**)

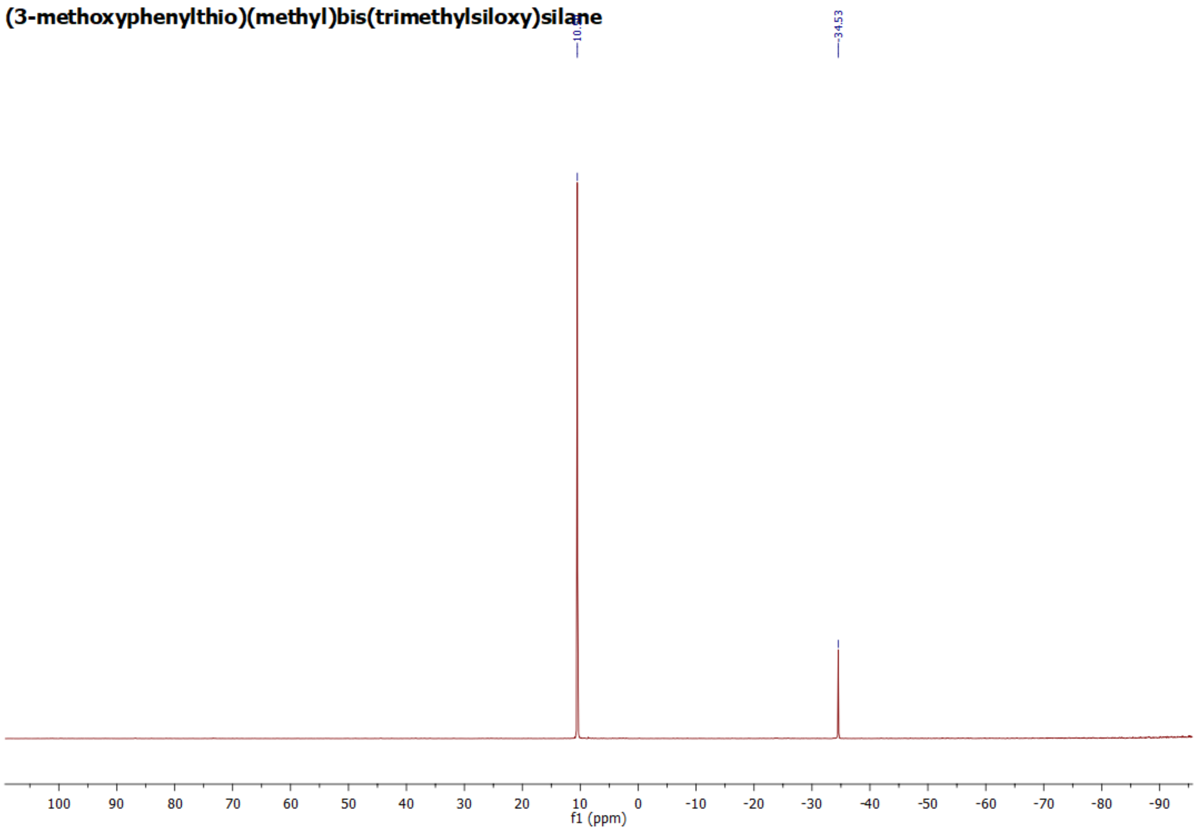

Figure S29. ^{29}Si NMR (79 MHz, $CDCl_3$) of (3-methoxyphenylthio)(methyl)bis(trimethylsiloxy)silane (**3 e-e**)

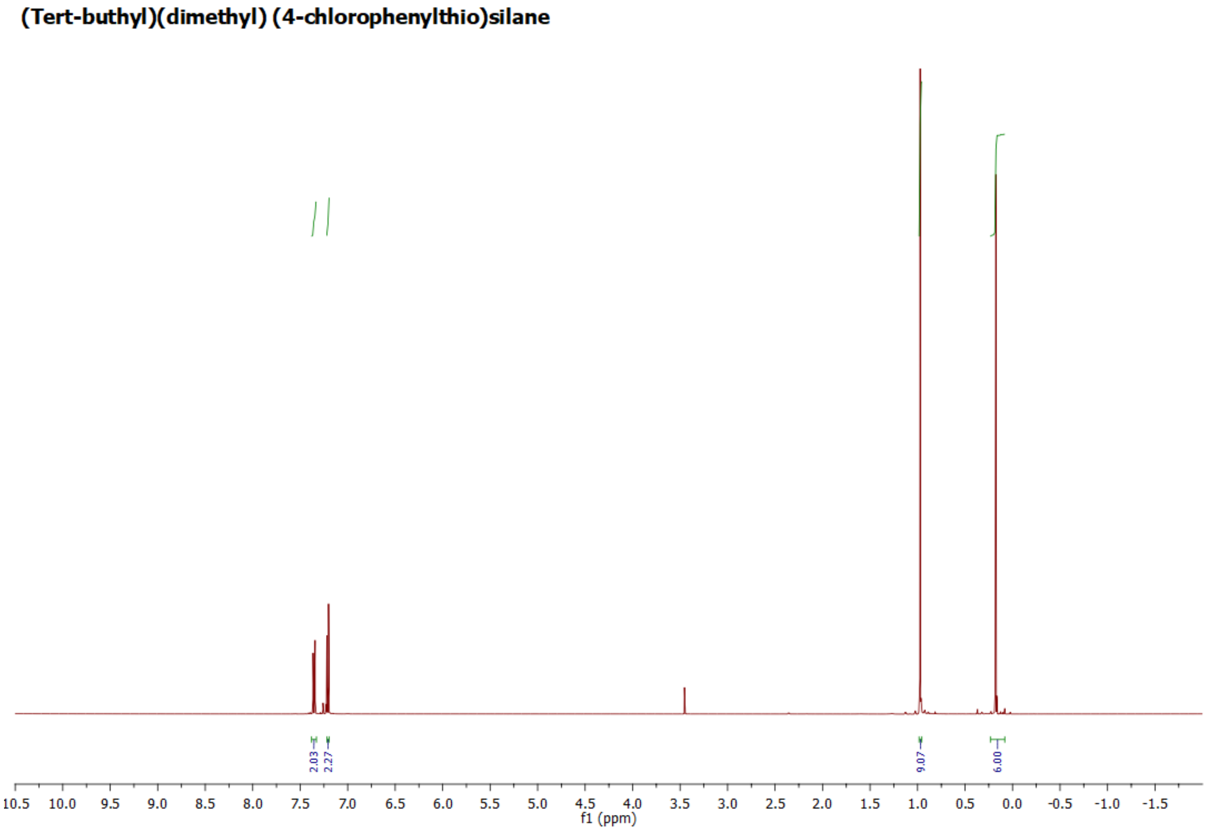

Figure S30. ^{1}H NMR (400 MHz, $CDCl_3$) of (*t*-buthyl)(dimethyl)(4-chlorophenylthio)silane (**3 b-f**)

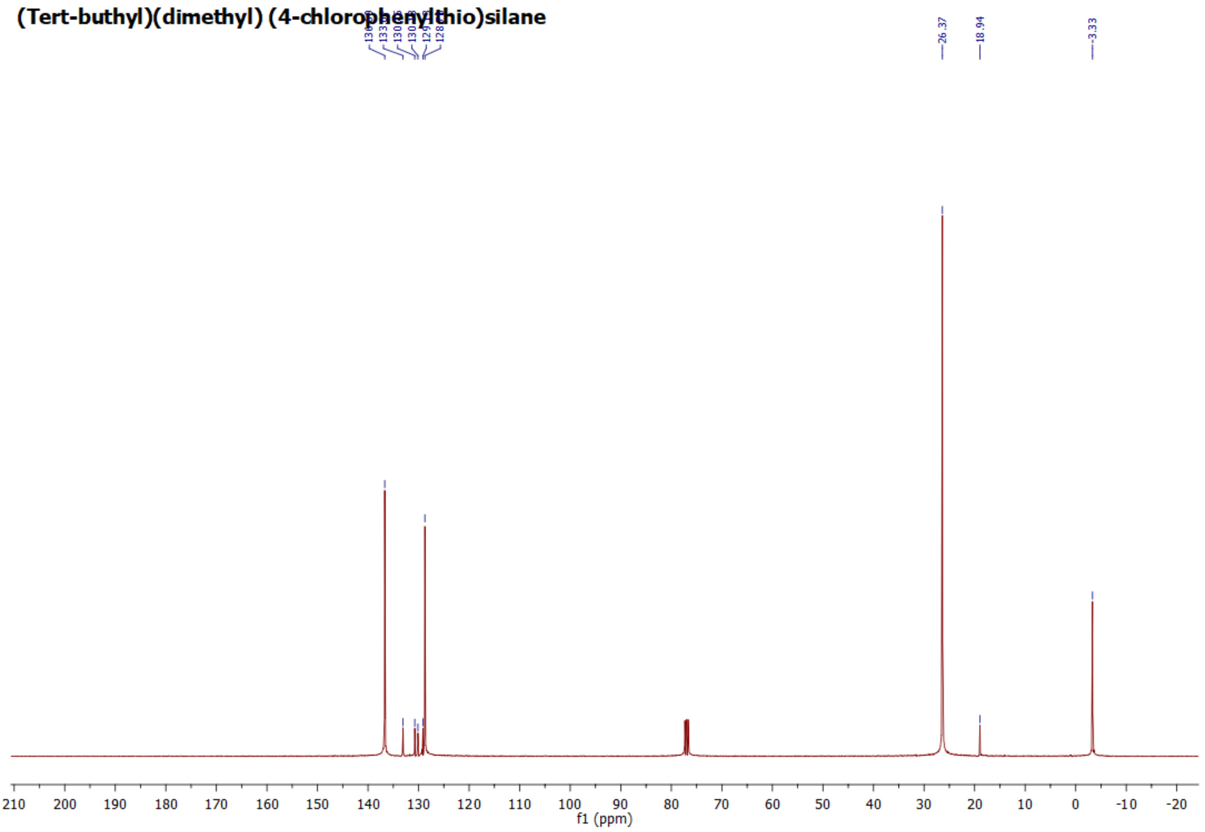

Figure S31. ^{13}C NMR (100 MHz, $CDCl_3$) of (*t*-buthyl)(dimethyl)(4-chlorophenylthio)silane (**3b-f**)

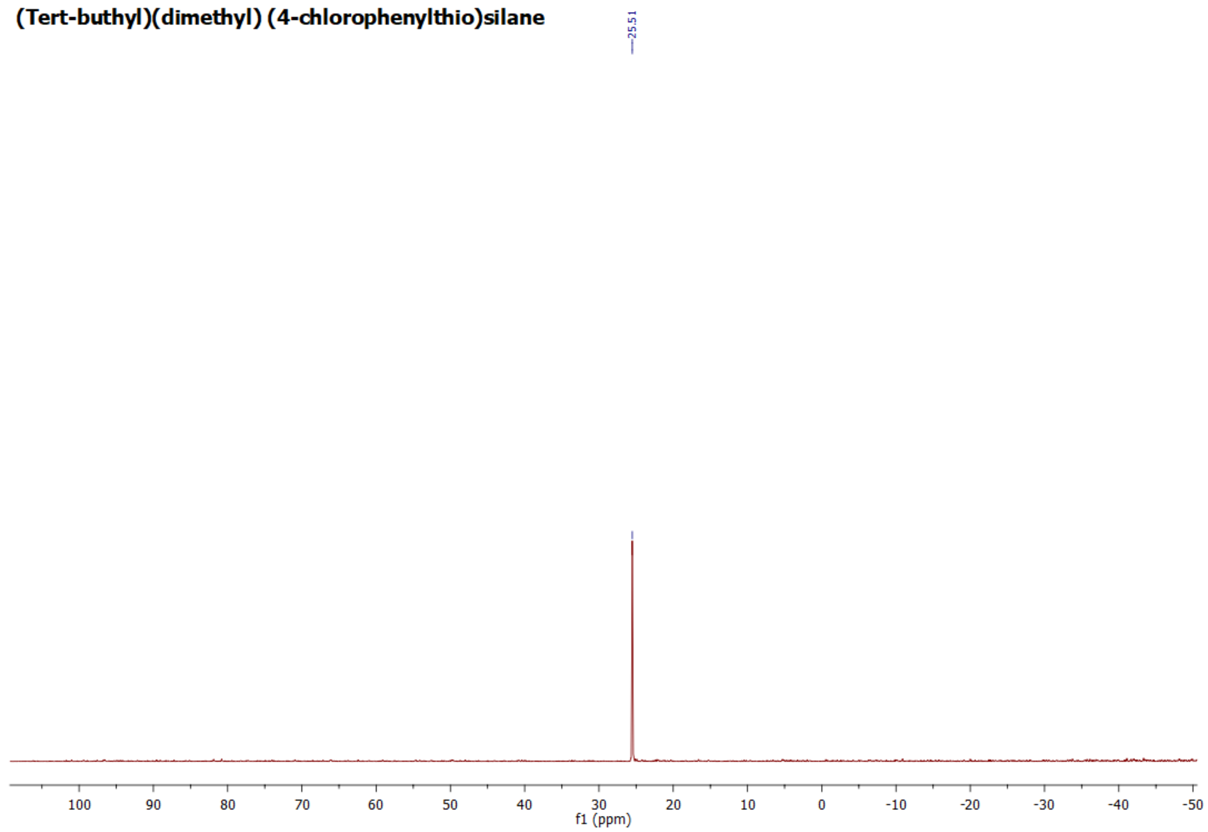

Figure S32. ^{29}Si NMR (79 MHz, $CDCl_3$) of (*t*-buthyl)(dimethyl)(4-chlorophenylthio)silane (**3 b-f**)

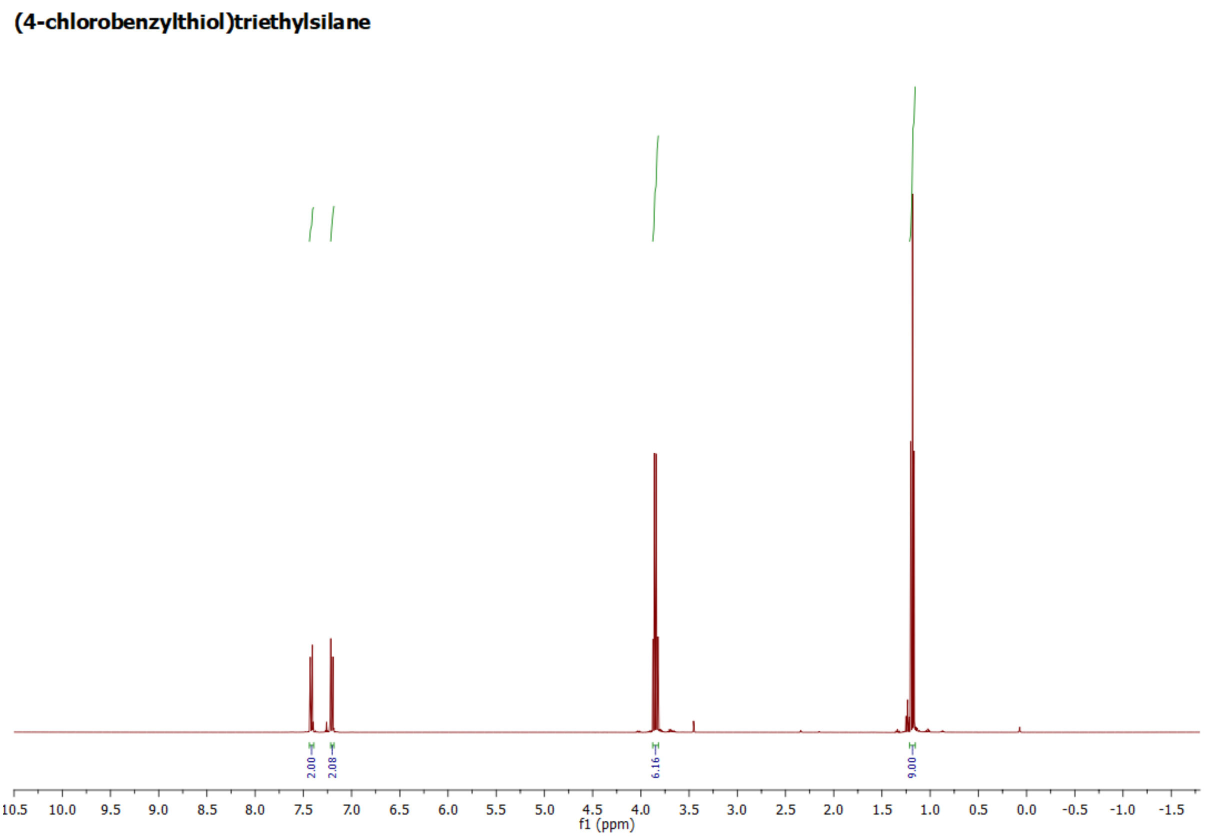

Figure S33. ^{1}H NMR (400 MHz, $CDCl_3$) of (4-chlorobenzylthiol)triethylsilane (**3 c-f**)

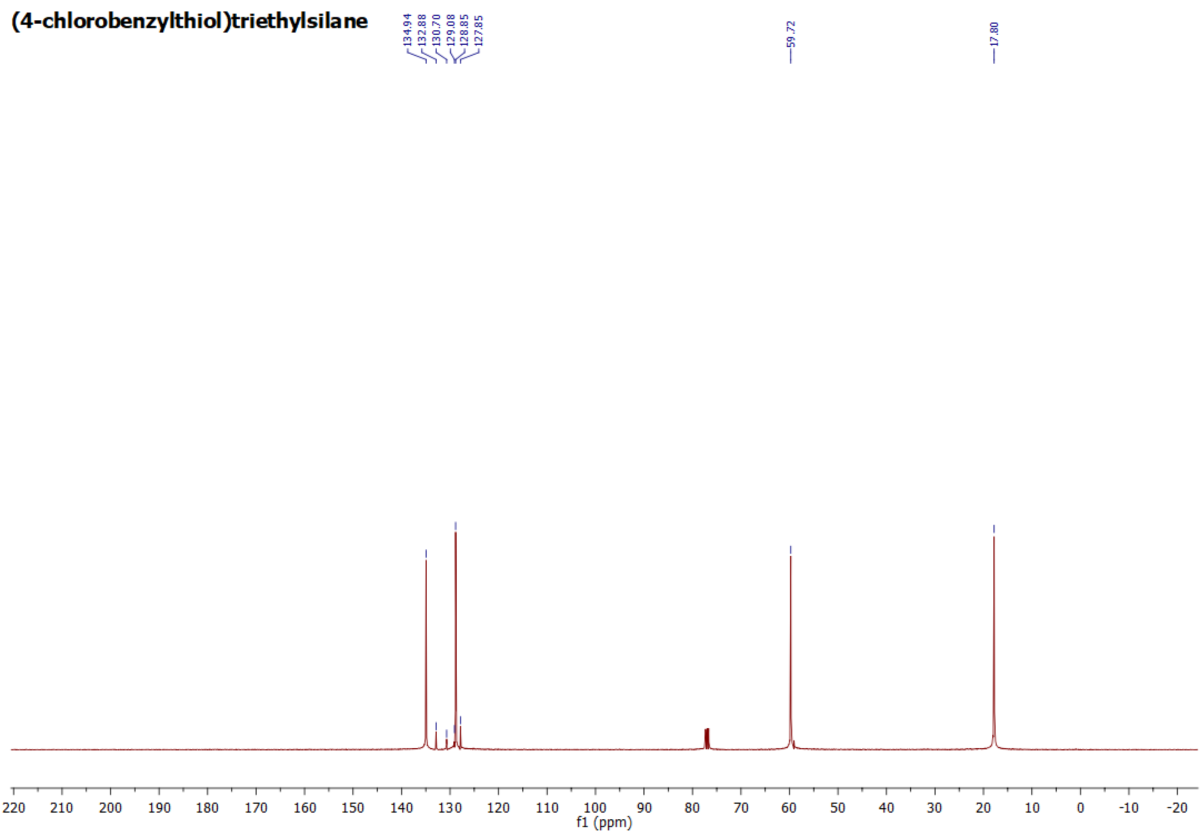

Figure S34. ^{13}C NMR (100 MHz, $CDCl_3$) of (4-chlorobenzylthiol)triethylsilane (**3 c-f**)

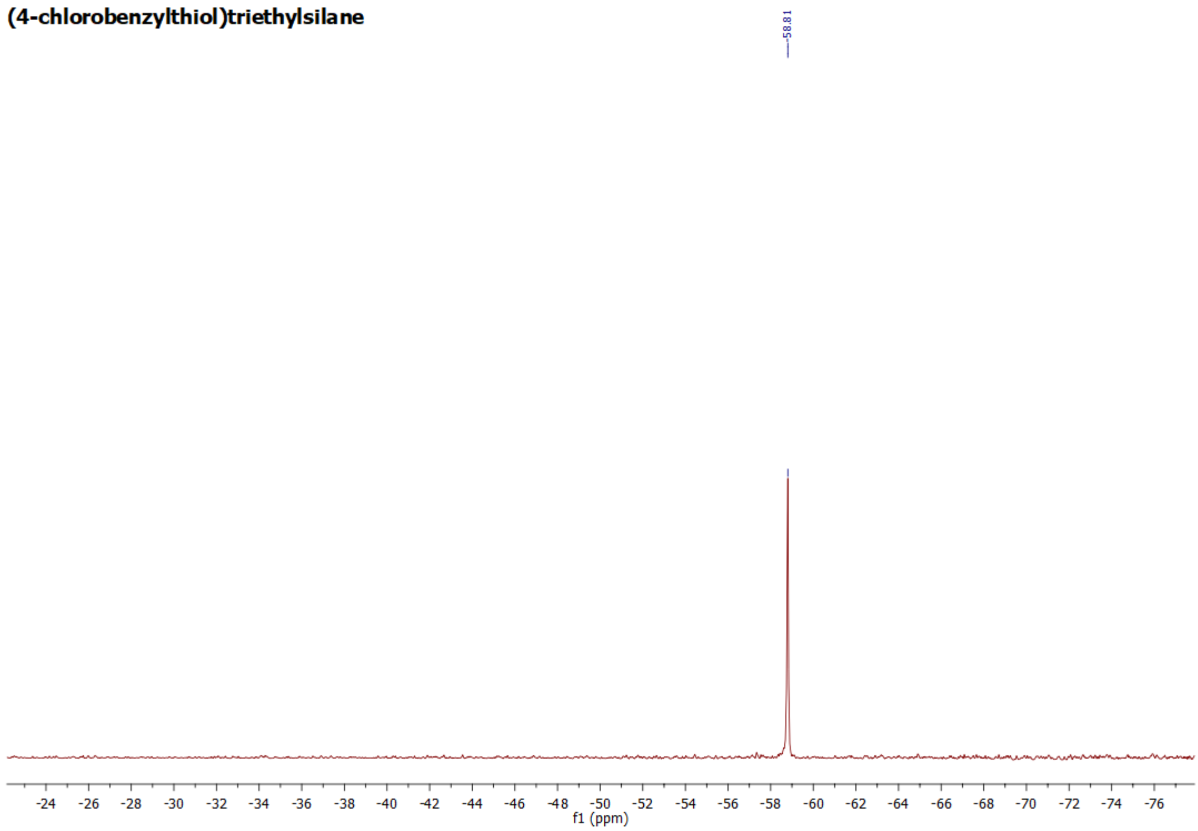

Figure S35. ^{29}Si NMR (79 MHz, $CDCl_3$) of (4-chlorobenzylthiol)triethylsilane (**3 c-f**)

12.2. NMR spectra of disulphides

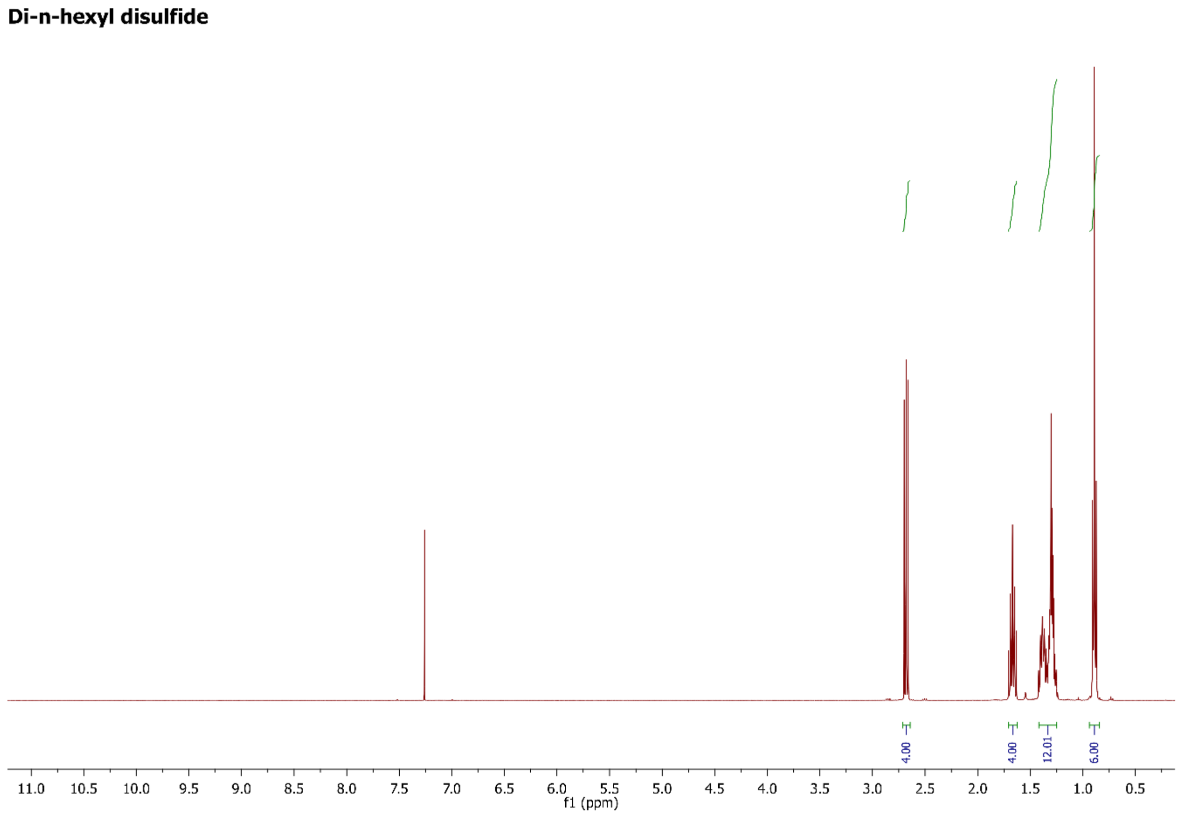

Figure S36. ^{1}H NMR (400 MHz, $CDCl_3$) of di-*n*-hexyl disulphide (**4a**)

Di-n-hexyl disulfide

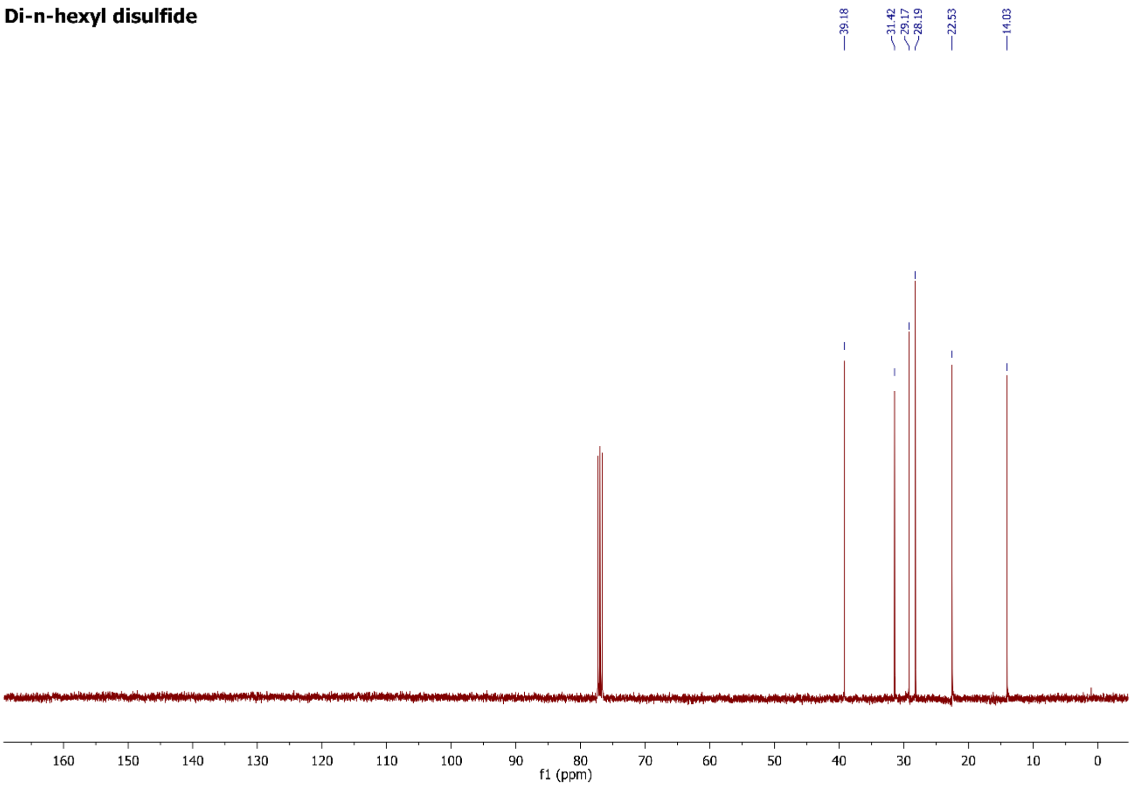

Figure S37. ^{13}C NMR (101 MHz, $CDCl_3$) of di-*n*-hexyl disulphide (**4a**)

Dipropyl disulfide

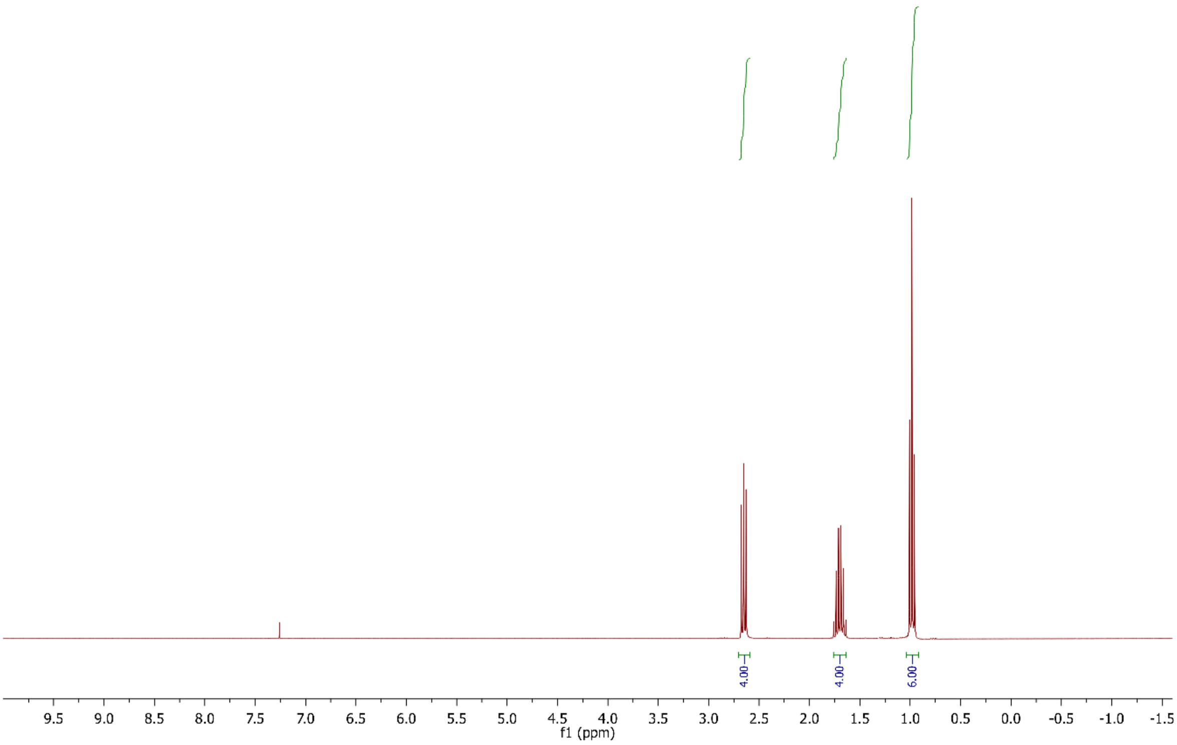

Figure S38. ^{1}H NMR (400 MHz, $CDCl_3$) of dipropyl disulphide (**4b**)

Dipropyl disulfide

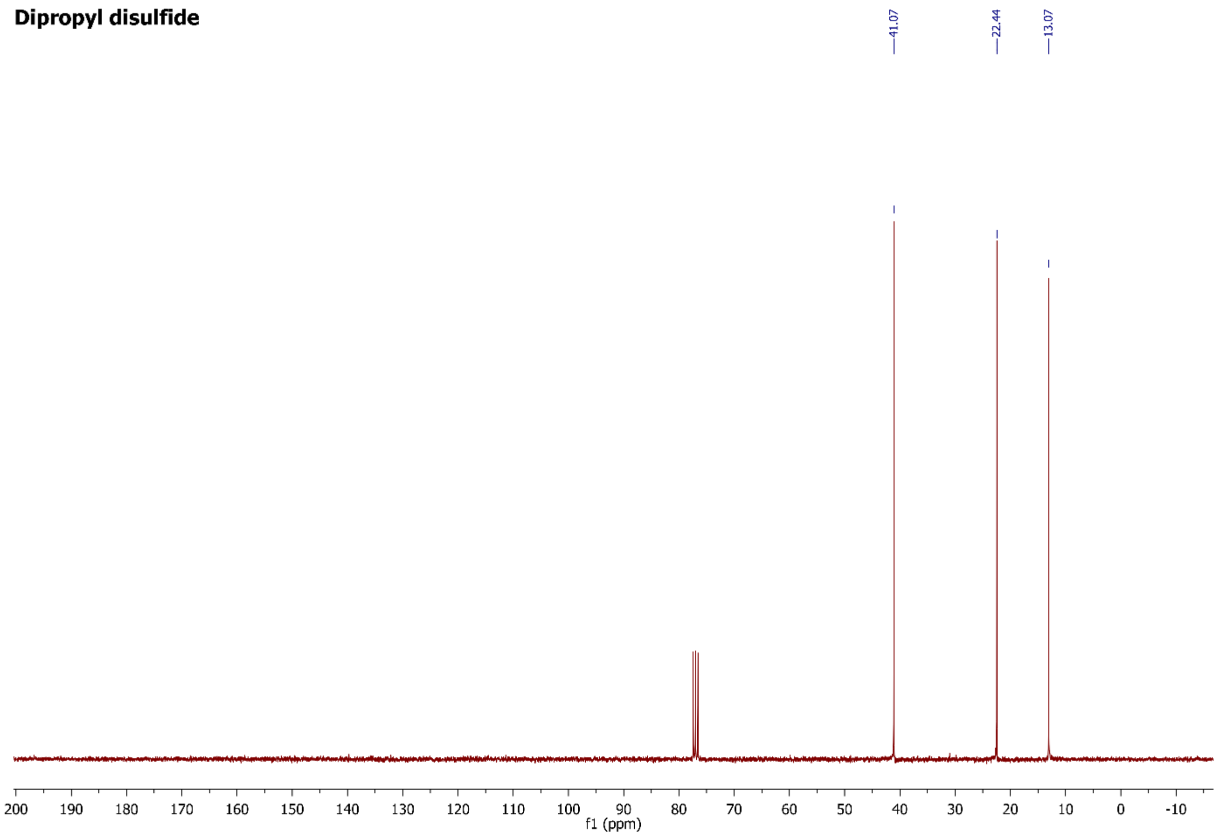

Figure S39. ^{13}C NMR (101 MHz, $CDCl_3$) of dipropyl disulphide (**4b**)

Dibenzyl disulfide

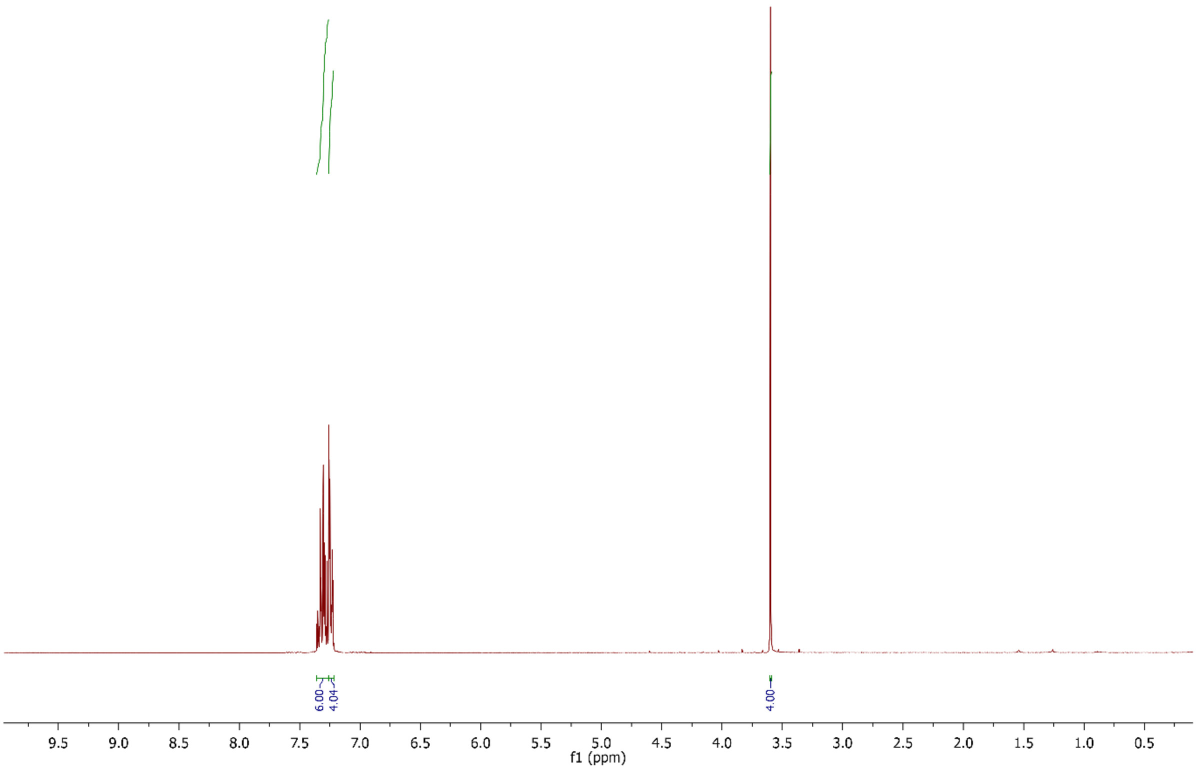

Figure S40. ^{1}H NMR (300 MHz, $CDCl_3$) of dibenzyl disulphide (**4d**)

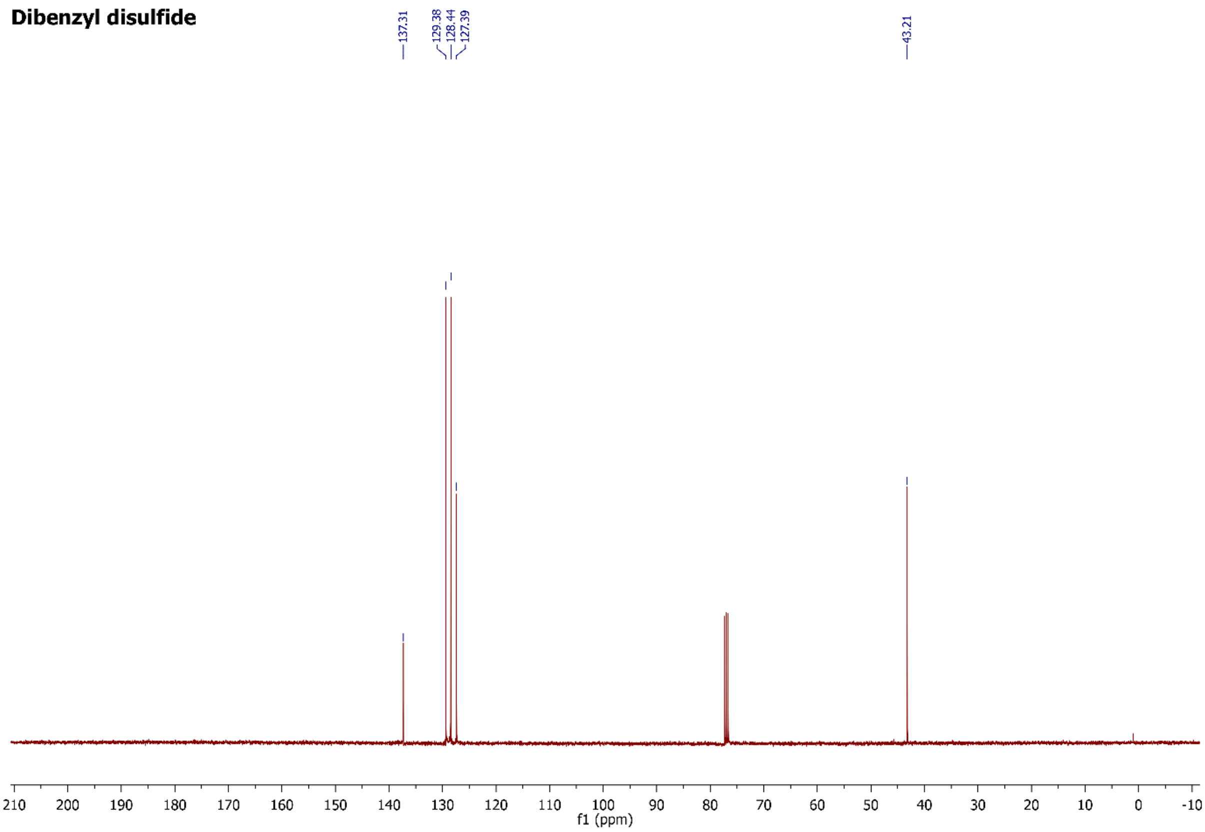

Figure S41. ^{13}C NMR (100 MHz, $CDCl_3$) of dibenzyl disulphide (**4d**)

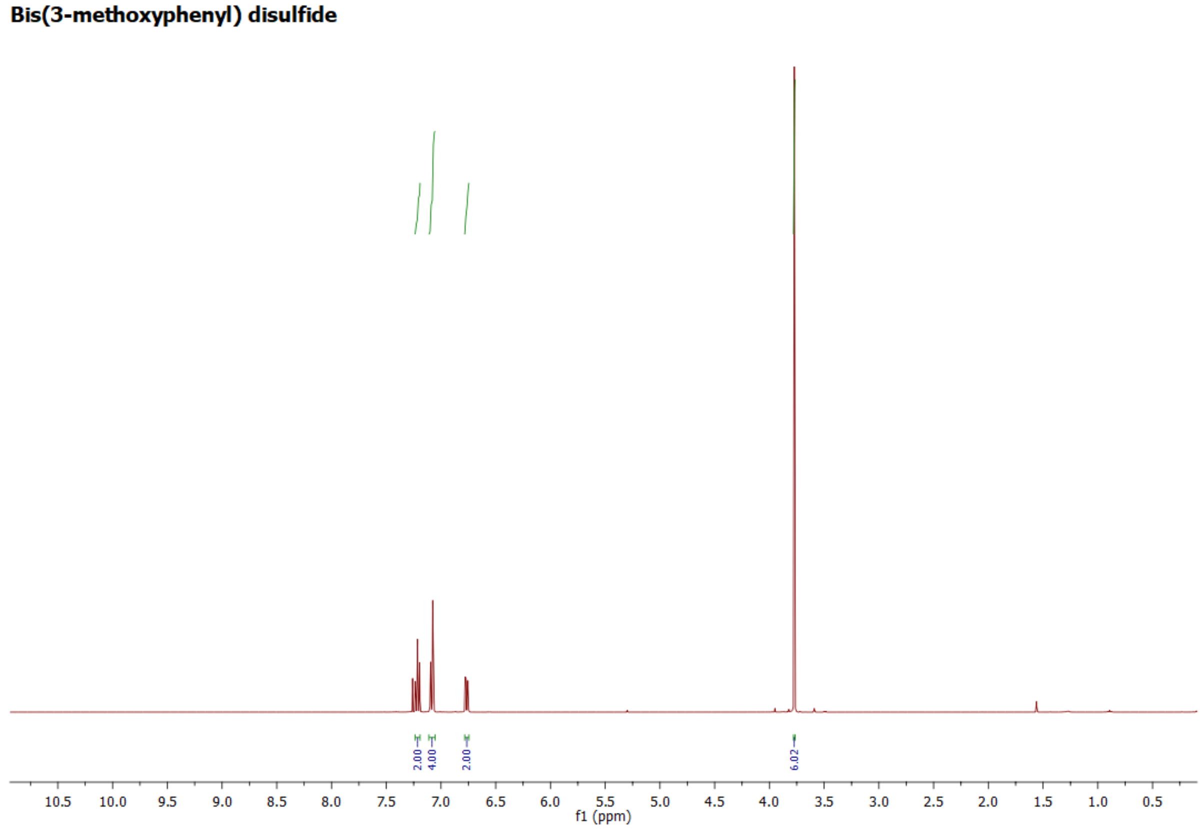

Figure S42. ^{1}H NMR (400 MHz, $CDCl_3$) of bis(3-methoxyphenyl) disulphide (**4e**)

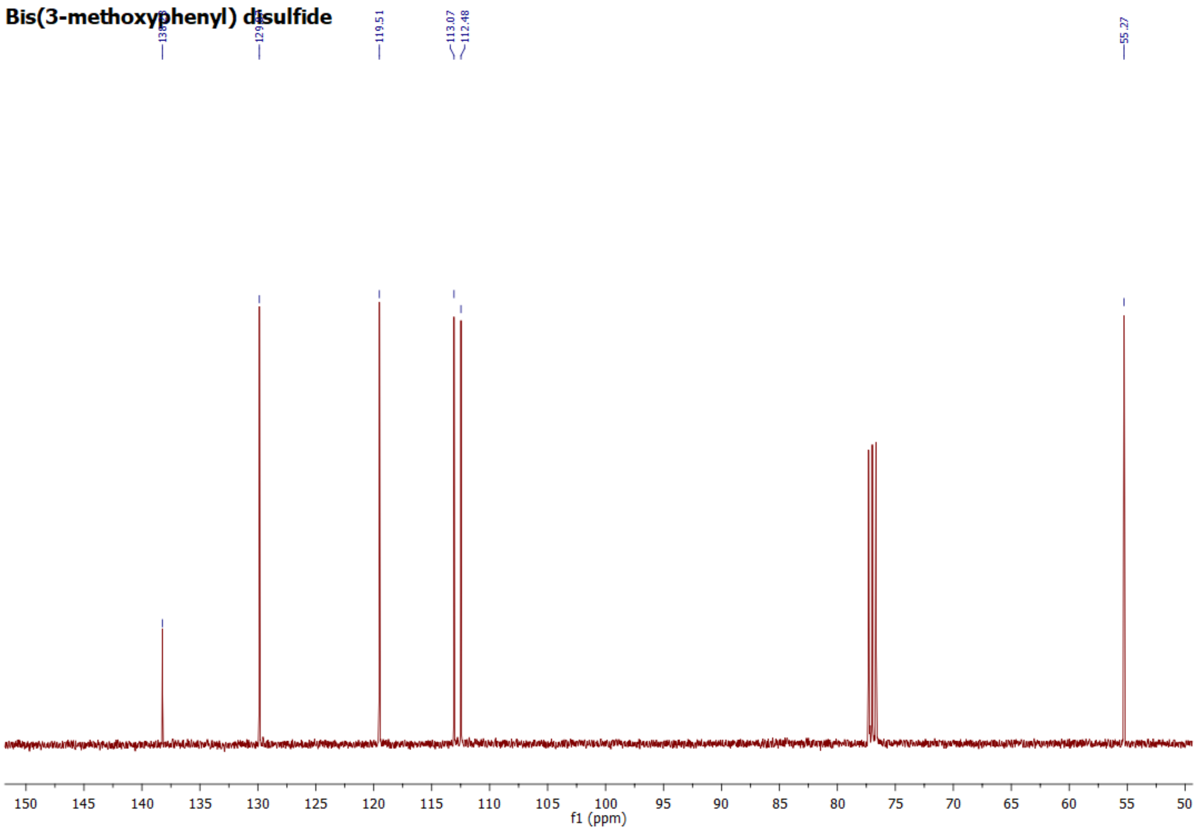

Figure S43. ^{13}C NMR (75 MHz, $CDCl_3$) of bis(3-methoxyphenyl) disulphide (**4e**)

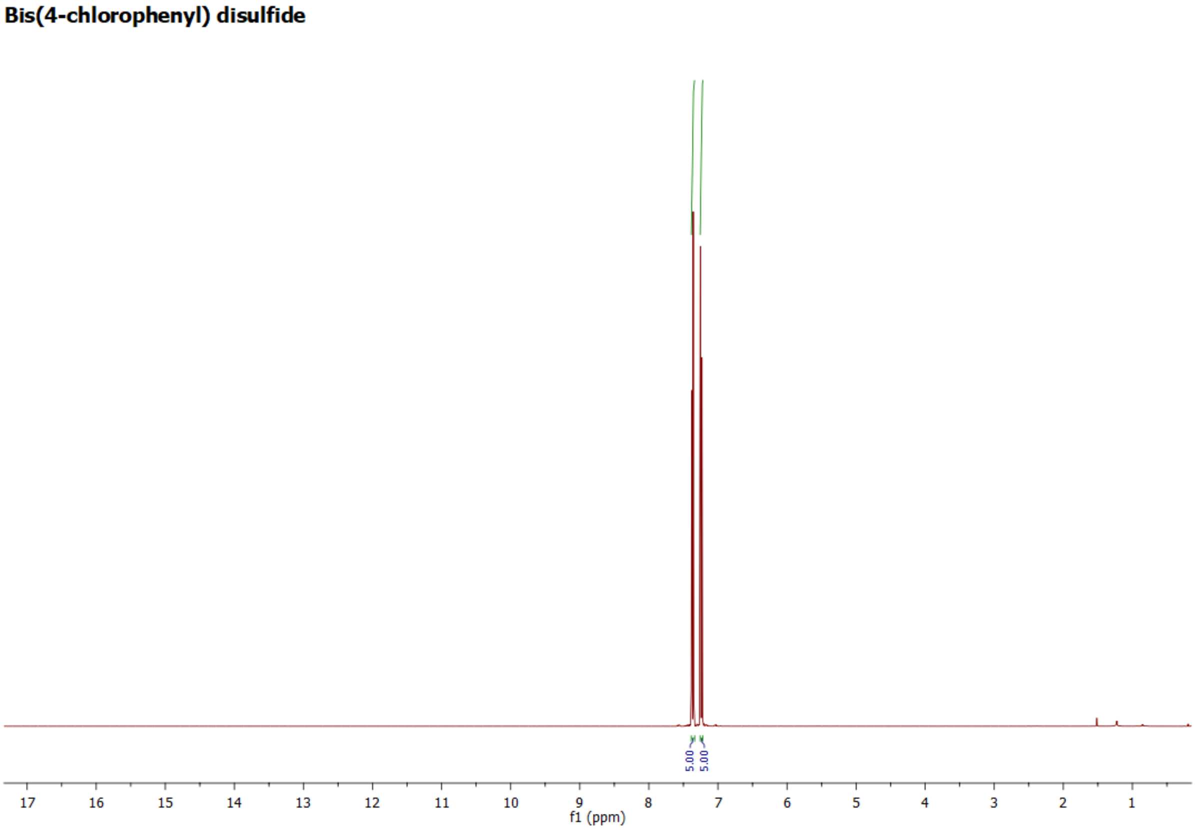

Figure S44. ^{1}H NMR (300 MHz, $CDCl_3$) of bis(4-chlorophenyl) disulphide (**4f**)

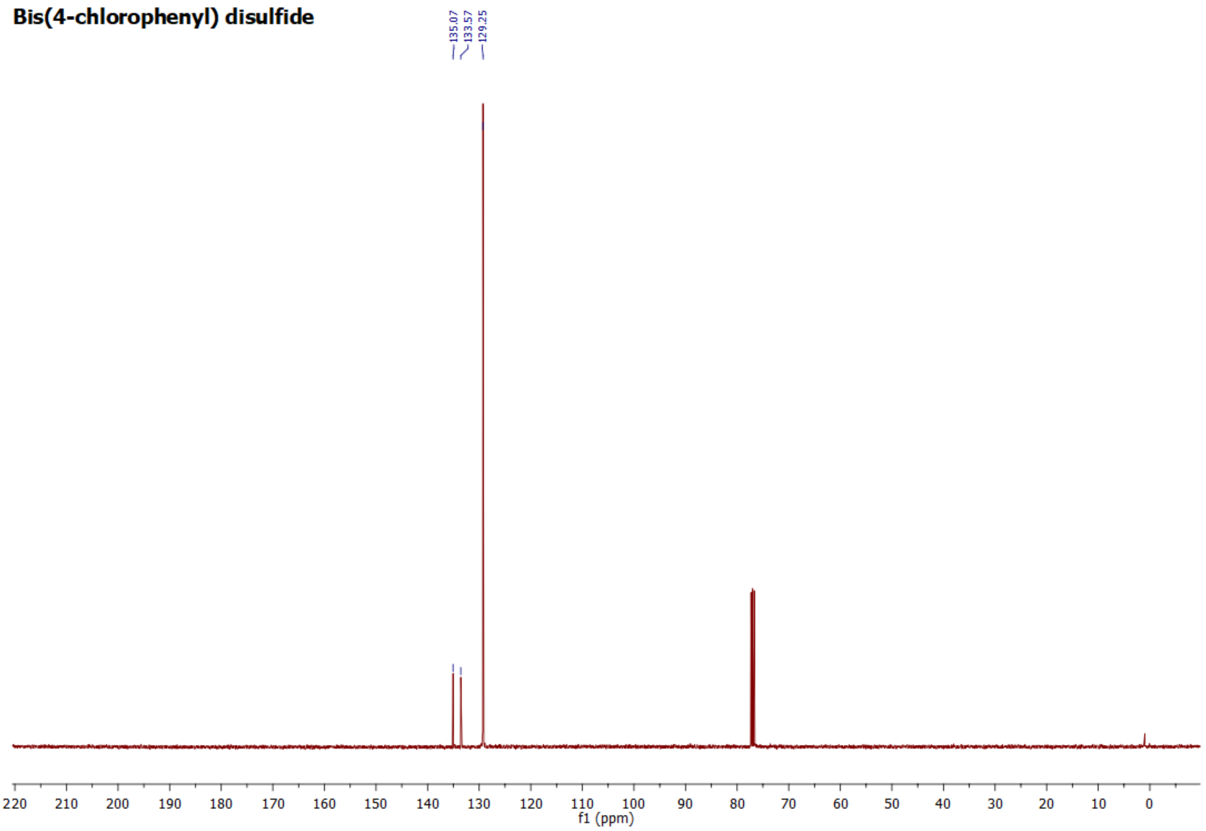

Figure S45. ^{13}C NMR (100 MHz, $CDCl_3$) of bis(4-chlorophenyl) disulphide (**4f**)

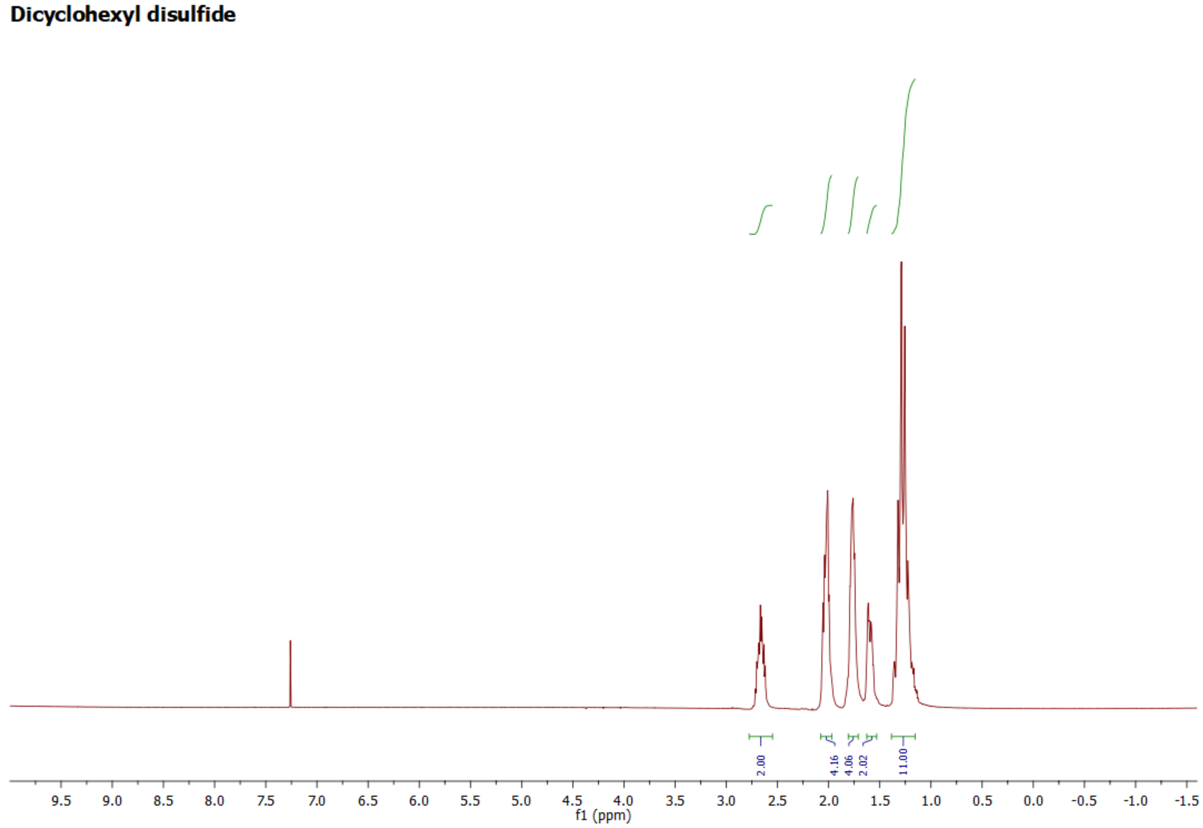

Figure S46. ^{1}H NMR (300 MHz, $CDCl_3$) of dicyclohexyl disulphide (**4h**)

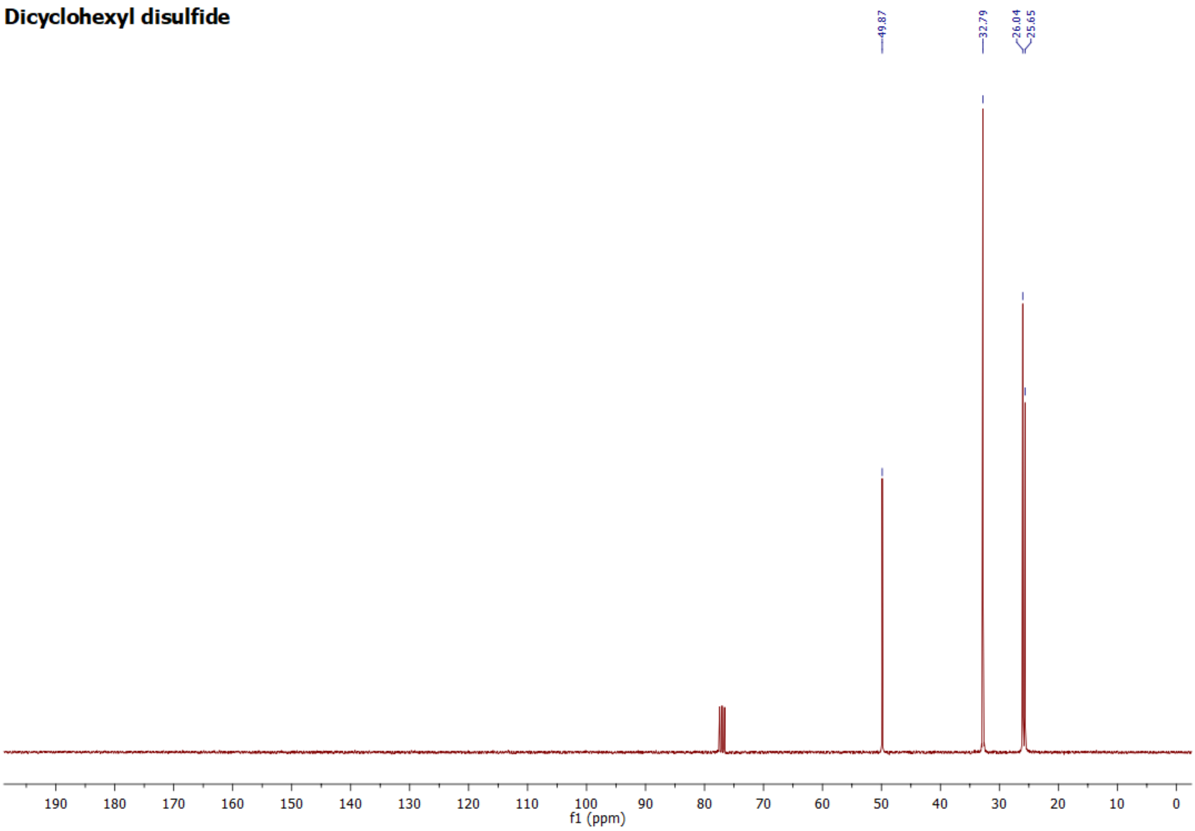

Figure S47. ^{13}C NMR (75 MHz, $CDCl_3$) of dicyclohexyl disulphide (**4h**)

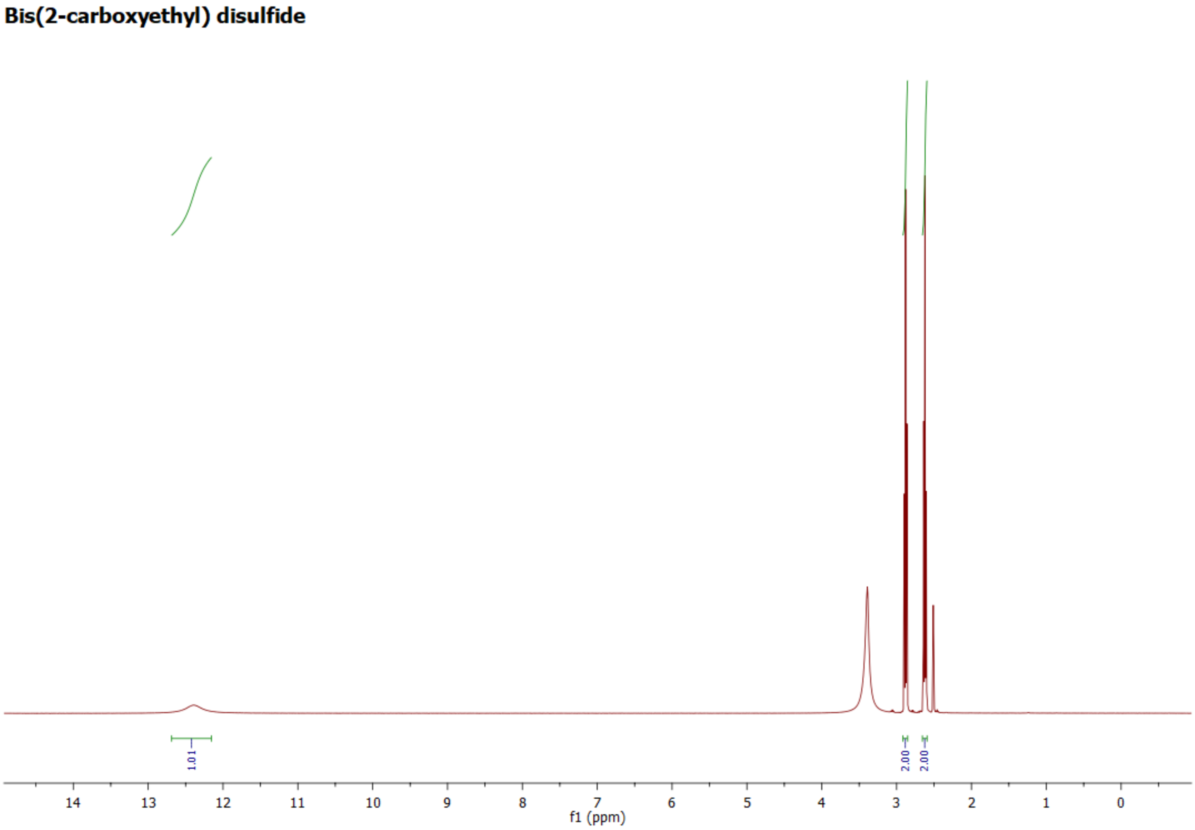

Figure S48. ^{1}H NMR (300 MHz, $CDCl_3$) of bis(2-carboxyethyl) disulphide (**4i**)

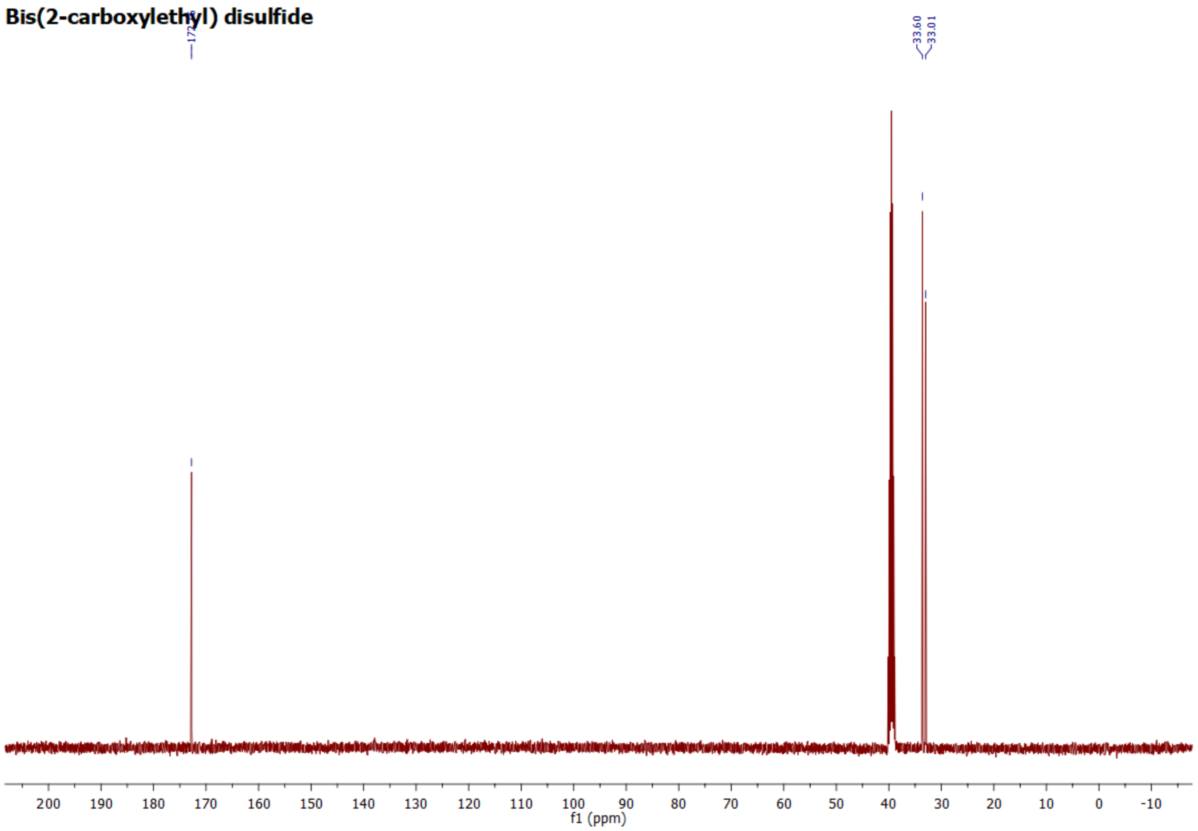

Figure S49. ^{13}C NMR (100 MHz, $CDCl_3$) of bis(2-carboxyethyl) disulphide (**4i**)

14. References

[1] M. Hans, J. Lorkowski, A. Demonceau, L. Delaude, *Beilstein J. Org. Chem.,* 2015, **11**, 2318.
